# Long-read metagenomic sequencing negates inferred loss of cytosine methylation in Myxosporea (Cnidaria: Myxozoa)

**DOI:** 10.1093/gigascience/giaf014

**Published:** 2025-03-13

**Authors:** Antonio Starcevic, Rayline T A Figueredo, Juliana Naldoni, Lincoln L Corrêa, Beth Okamura, Edson A Adriano, Paul F Long

**Affiliations:** Laboratory for Bioinformatics, Department of Biochemical Engineering, Faculty of Food Technology and Biotechnology, University of Zagreb, Zagreb HR-10000, Croatia; Department of Animal Biology, Institute of Biology, University of Campinas, Campinas, 13083-970, SP, Brazil; Department of Pathology, University of Cambridge, Cambridge CB2 1QP, United Kingdom; Institute of Water Sciences and Technology, Federal University of Western Pará (UFOPA), Santarém, 68040-255, PA, Brazil; Life Sciences, Natural History Museum, London I SW7 5BD, United Kingdom; Universidade Federal de São Paulo, Instituto de Ciências Ambientais, Químicas e Farmacêuticas, Diadema, 09972-270, SP, Brazil; Institute of Pharmaceutical Science, King’s College London, London SE1 9NH, United Kingdom; Faculdade de Ciências Farmacêuticas, Universidade de São Paulo, 05508-000 São Paulo, SP, Brazil

**Keywords:** cnidarians, DNA methylation, long-read sequencing, bioinformatics

## Abstract

Oxford-Nanopore PromethION sequencing is a PCR-free method that retains epigenetic markers and provides direct quantitative information about DNA methylation. Using this long-read sequencing technology, we successfully assembled 5 myxozoan genomes free from discernible host DNA contamination, surpassing previous studies in both quality and completeness. Genome assembly revealed DNA methylation patterns within myxozoan genomes, particularly in GC-rich regions within gene bodies. The findings not only refute the notion of myxozoans lacking DNA methylation capability but also offer a new perspective on gene regulation in these parasites. The high-quality genome assemblies lay a solid foundation for future research on myxozoans, including new strategies to control these commercially significant fish pathogens.

## Graphical Abstract

**Graphical Abstract:**
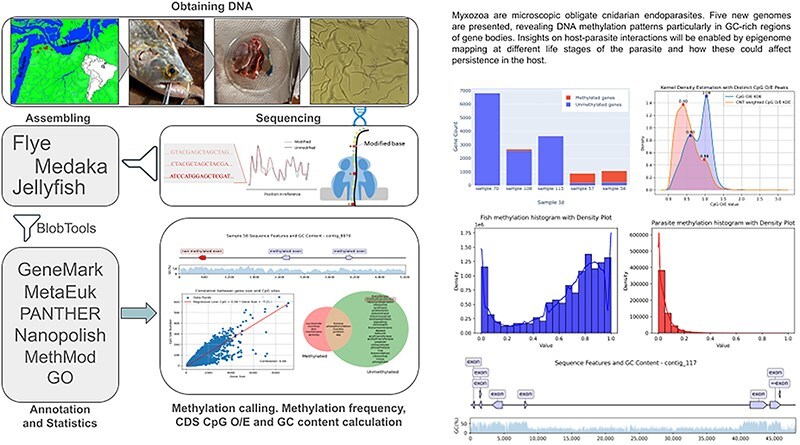

## Introduction

Epigenetic processes enable cells to control gene activity without altering DNA sequences [1]. Cytosine methylation is an epigenetic mechanism widely found in eukaryotes and involves DNA methyltransferases (DNMTs) that transfer a methyl group from S-adenosylmethionine to the C5 position of cytosine of genomic DNA [2]. Such cytosine methylations are subsequently recognized by methyl-CpG binding domain proteins (MeCP2 and MBD1–4), leading to transcriptional silencing and the subsequent generation of phenotypic variation [3–6]. Together, DNMTs and MBDs, when complexed with other proteins, comprise the core metazoan DNA methylation system found in both vertebrates and invertebrates [7]. The regulation of gene expression achieved by cytosine methylation is linked with many key processes in animals, including gametogenesis, embryonic development, cellular differentiation, X-chromosome inactivation, and transposon repression [8]. Most investigations of cytosine methylation have been conducted on free-living animals, but some have focused on endoparasites, where they have been linked with persistence in hosts. For example, cytosine methylation in the cestode, *Taenia solium*, is associated with key parasitism-related genes (secretory proteins), leading to the suggestion that targeting DNA methylation processes may offer a therapeutic strategy [9].

Cytosine methylation contributes to key biological processes and is viewed as highly conserved, occurring in viruses, prokaryotes, and eukaryotes [5, 10, 11]. Nevertheless, cytosine methylation is absent in the nematode *Caenorhabditis elegans* [12]. There is also evidence for loss in all dipterans and some hymenopterans [13] and possibly in the helminth *Schistosoma mansoni* [14, 15]. Knowledge of distinct epigenetic processes will expand our understanding of gene regulation and provide more nuanced views on the relevance of model organisms, such as *C. elegans* and *Drosophila melanogaster*. Alternative mechanisms of DNA modification, such as adenine methylation [16], may act as a substitute in invertebrates (*C. elegans* [12], *D. melanogaster* [17]), in mammals (mouse embryonic stem cells [18]), and in plants (*Arabidopsis thaliana* [19]). However, it is not always clear what replacement mechanisms may be operating for organisms that appear to have lost cytosine methylation [11].

Another epigenetic mechanism widely found in eukaryotes, including parasites, involves modification of histone proteins [20]. Control of histone gene expression ensures that a fine balance is maintained between histone abundance, correct packaging of DNA into chromosomes, and regulation of DNA transcription [21]. Differential expression of histones has been linked with endoparasitism, for example, in eukaryotic microbial organisms such as *Plasmodium falciparum* [22], *Leishmania infantum* [23], *Trypanosoma cruzi* [24], and the myxozoan *Myxobolus bejeranoi* [25]. Transcriptomic analyses of *M. bejeranoi* infecting cultured tilapia hosts also provided evidence for regulation of gene silencing by microRNA, which is a widely recognized epigenetic mechanism associated with host–parasite interactions [26, 27].

Histone deacetylation and CpG methylation are interconnected in several ways, including acting in concert to effect one of the more important epigenetic processes—gene silencing [28]. When histone deacetylases remove acetyl groups from histone proteins, it leads to a more condensed chromatin structure, making DNA less accessible for transcription factors and RNA polymerase. Methylation of CpG islands in promoter regions or within genes themselves (a process called gene body methylation) can repress gene transcription. Moreover, methylated CpG sites can recruit proteins like methyl-CpG binding proteins [29]. These proteins can, in turn, recruit histone deacetylases to further compact chromatin and inhibit gene expression. These collective processes can create a reinforcing loop with histone deacetylation and DNA methylation working together to maintain gene silencing. Certain multiprotein complexes, such as the MeCP1 complex, contain both DNA methyltransferases (which add methyl groups to CpG sites) and histone deacetylases [30], which indicates a direct physical interaction that promotes these 2 epigenetic mechanisms [31]. The exact outcome of this interaction depends on the specific context, genomic location, and interacting proteins.

Recently, Kyger et al. [10] proposed that most myxozoans lack cytosine methylation based on analyses of 2 distant species of myxosporeans—the clade comprising most myxozoan species [32, 33]. They first searched for methylation-associated proteins in transcriptomic and genomic data from 29 cnidarians, including 8 myxosporean species. They then conducted whole-genome bisulfate sequencing (WGBS) of 2 distantly related myxosporeans to determine which cytosine residues were methylated. Neither approach provided evidence for cytosine methylation in myxosporeans. However, they detected methyl-associated proteins in many free-living cnidarians, and the cytosine methylation-associated protein, MDB1/2/3, was identified in a limited EST library of the malacosporean myxozoan, *Buddenbrockia plumatellae*. This collective evidence led Kyger et al. [10] to conclude that myxosporeans are one of the few confirmed instances of animals that have secondarily lost cytosine methylation capability. They proposed that cytosine methylation has been retained, or is in the process of being lost, in the taxon-poor sister clade—the Malacosporea.

Although the WGBS method used by Kyger et al. [10] is a powerful approach, various associated processes can lead to false-negative results. If the bisulfite conversion reaction is incomplete, some unmethylated cytosines may not be converted to uracil, leading to false-negative results [34]. DNA fragmentation or degradation can also result in the loss of methylated cytosines. Furthermore, PCR amplification can introduce biases in the representation of methylated and unmethylated DNA sequences. Critically, the presence of host DNA greatly challenges characterizing endoparasites using untargeted next-generation sequencing (NGS) approaches. In particular, untargeted DNA sequencing of all genomes within a sample produces highly complex datasets with millions of short reads representing different genome fragments [35]. In this genomic mixture, host DNA may potentially overwhelm endoparasite signals during assembly. Thus, if only a limited fraction of the DNA derives from endoparasites, assemblies based on multiple samples and/or host DNA sequence filtering may be required to obtain reasonable coverage of endoparasite genomes. Finally, DNA methylation patterns can also be variable between cell types (i.e., host vs. endosymbiont) or different endoparasite unicellular stages (i.e., plasmodia vs. spores in the case of myxosporeans), potentially leading to false-negative results if the methylated regions are not well represented in the sample [36]. To address these issues, it is important to sequence multiple independent samples or to use alternative sequencing methods.

Myxozoa comprise a bizarre radiation of extremely morphologically simplified parasitic cnidarians whose extensive molecular divergence long precluded their phylogenetic placement [37, 38]. However, it is now clear that they evolved within Cnidaria [39–41] and comprise some 20% of all described cnidarian species in the present day [33]. Furthermore, the speciose and diverse myxosporeans are highly derived and demonstrate a unique convergence to eukaryotic microparasitic lifestyles by developing exclusively as plasmodia and various invasive unicellular stages [37, 42]. This evolutionary trajectory may ultimately have been facilitated by the diploblast nature of cnidarians [43]. The discovery of vermiform plasmodia in gallbladders of Amazonian fishes, in turn, demonstrates convergence to metazoan worm-like forms achieved at the unicellular level by myxosporeans [44]. It is of considerable interest, therefore, to confirm whether proposed loss of cytosine methylation may be associated with the rapid evolution and the unique morphological trajectories involving miniaturization displayed by myxozoans. However, as outlined above, further confirmation of this loss is required in view of potential complications associated with bisulfite sequencing using the small read-length generating Illumina HiSeq platform adopted by Kyger et al. [10]. Oxford-Nanopore PromethION sequencing (ONT) is a PCR-free method that retains epigenetic markers and provides direct quantitative information about DNA methylation [45]. The aim of this study was to use ONT as an alternative method to ascertain loss of cytosine methylation in myxosporeans and to generate the first long sequence reads for myxozoan genomes.

## Methods

### Fish sampling

Sampling and access to genetic heritage were authorized by the Brazilian Ministry of the Environment (SISBIO authorization #67616–2 and SisGen #A656D). The study was approved by the Ethics Committee on Animal Use of the Federal University of São Paulo-UNIFESP (CEUA #6549290920). Fish were caught during January 2022 using gill nets in waters where the Tapajós and Amazon Rivers merge, close to the city of Santarém, Pará State, Brazil (2°23′49.79″S 54°43′53.33″W). The fish were transported live to a makeshift field laboratory, close to the sampling sites, and euthanized prior to dissection. Our aim was to sample opportunistically for myxozoans in Amazonian fish gallbladders, which have been commonly reported to be infected by these cnidarian parasites [44]. After the dissection of each fish, the gallbladder was removed, and a sterile scalpel blade was used to open the gallbladder, pouring the bile directly into a sterile 1.5-mL Eppendorf tube. With a sterile micro-pipette, a drop of the bile fluid was immediately placed on a microscope slide and examined using a light microscope (Carl Zeiss model Primo Star) at 20× or 40× to ascertain infection. When infection was confirmed, the bile fluid content of the Eppendorf tube was immediately preserved in DNA/RNA Shield reagent (Zymo Research Corp) to give 50% (v/v) in a final sample volume of 900 µL. Pictures of parasites in bile were obtained using a digital camera (Sony CyberShot) coupled to the light microscope objective. Observations of plasmodia and, especially, of diagnostic myxospores with polar capsules enabled microscopic identification of myxozoan infections.

### DNA isolation and sequencing

**Figure 1:**
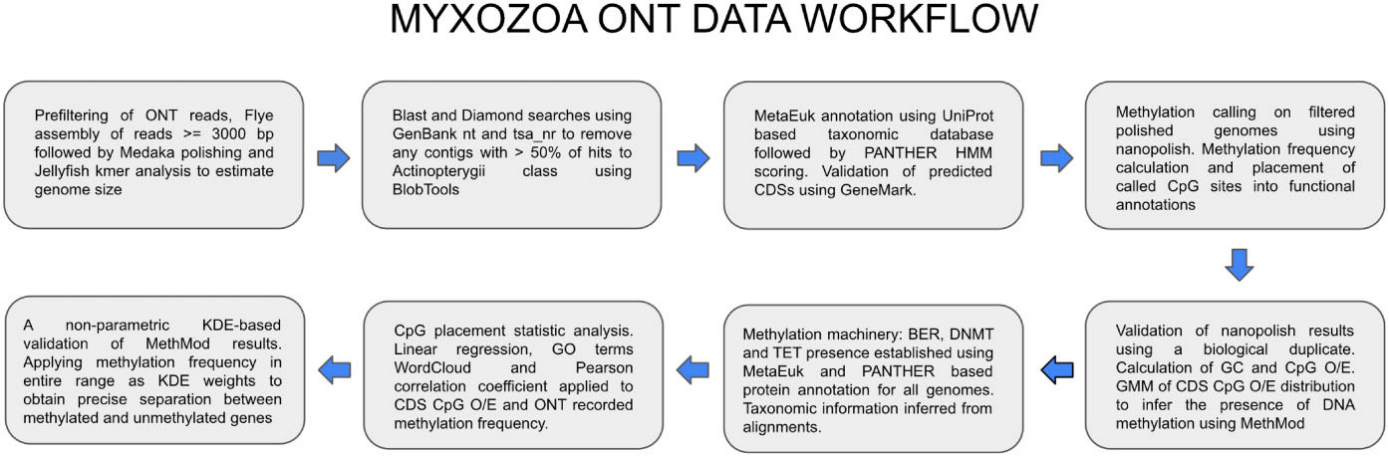
Overview of the bioinformatics pipeline. Steps used to assemble myxozoan genomes from Oxford Nanopore long sequence reads and to detect the exact location of methylated bases (5-methylcytosine in a CpG context) within each assembled genome.

Metagenomic DNA was extracted from each sample using the Quick-DNA Fecal/Soil Microbe DNA Miniprep Kit (Zymo Research) in accordance with the manufacturer’s instructions. The entire sample was added to a ZR BashingBead Lysis Tube and the first step of the extraction proceeded by bead bashing (Vortex-Genie 2; USA Scientific) at 3,200 rpm for 2 minutes, followed by a rest on ice for 1 minute, repeating these steps 3 times. The concentration of DNA was measured using a NanoDrop 2000 spectrophotometer (Thermo Scientific). Metagenomic libraries were generated using ∼200 ng DNA and sequenced at the Novogene Sequencing Centre. Crucially, the standard workflow deviated from the sequencing center’s proprietary methodology in 3 respects. First, DNA was not fragmented or size selected. Second, the 1-dimensional libraries were not purified following end-polishing, nick repair, and ligation of sequencing adaptors. Third, the libraries were pooled and sequenced, irrespective of the final library concentration, using an entire ONT sequencing cell (PromethION FLO-PRO002; RRID:SCR_017987). The original nanopore signal was recorded in Fast5 format, and base calling was achieved using Guppy (RRID:SCR_022353) [46] to produce FASTQ format. Quality control of the raw reads was conducted using NanoPlot software (RRID:SCR_024128) [47] to remove adapter contamination and low-quality reads. Only reads with Q7 > 7 were used in subsequent assemblies. An overview of the entire ONT data analysis pipeline is given in Fig. 1 and is next described in detail below.

### Removal of fish host contamination and assembly of Myxozoa reads

The first step to remove fish host DNA from each metagenomic DNA sample was to map reads onto a set of 21 complete reference genomes representing fish from the order Characiformes and family Sciaenidae (Supplementary File 1). Minimap2 (RRID:SCR_018550) [48] was used since this software allows mapping of long and noisy DNA reads to reference genomes with the “map-ont” option. All the reads that mapped to the fish reference genomes were removed as contaminants, leaving datasets comprising Myxozoa-enriched reads. After this first round of read filtering, the remaining reads having average quality score >Q7 and with lengths >3,000 bp used as inputs for Flye (RRID:SCR_017016) [49]. Flye is a *de novo* assembler for single-genome and metagenome sequencing reads, such as those produced by the ONT sequencing [49]. It is important to note that Flye works using uncorrected reads as inputs and produces error-corrected contigs as outputs without the need to specify the expected genome size, which is very important since the expected genome sizes for Myxozoa are largely an unknown variable. The quality of the assembled contigs was further enhanced using Medaka [50], which has been specifically trained to correct draft sequence outputs from the Flye assembler in a process called polishing, which pushes the error correction close to that expected from an assembly if it were constructed using Illumina-generated reads. Custom code was written to improve parallelism for this Medaka consensus-generating step.

To eliminate any remaining host and other contaminating DNA that might have evaded the first-stage minimap2-based filtering process (and thus might subsequently become erroneously assembled into chimeric contigs), a second, more stringent filtering step was introduced that used BlobToolKit v4.2.0 (RRID:SCR_025882) [51]. This is a tool designed to aid genome assembly quality control, contaminant detection, and filtering. This second-stage filtering process involved (i) pairwise BLAST [52] comparisons between the Medaka polished assembled contigs for each Myxozoa sample against the entire GenBank Eukaryota nt (nt_euk) database and (ii) Diamond [53] BLASTx comparisons against the protein portion of the Transcriptome Shotgun Assembly Sequence Database (tsa_nr). The hits from these 2 BLAST analyses provided inputs that the BlobToolKit used, together with read coverage information, to obtain taxonomic assignments for the assembled DNA. Any contigs that provided the majority of hits to host-related taxa (Actinopterygii) were removed. The outputs, therefore, were Medaka-polished contigs that represented Myxozoa genomes clean of any detectable fish host DNA.

Lastly, the genome size for each of the samples was predicted by comparing each assembly total size (in bp) with the genome size estimated by *k*-mer count analysis using error-corrected ONT reads. This approach was taken because Myxozoa are highly variable in terms of genome size, and no true reference genomes exist. To estimate the genome sizes, all the Medaka polished contigs and all the error-corrected long reads were separately used for 21-mer and 25-mer frequency analyses. Jellyfish v2.1.3 (RRID:SCR_005491) [54] was used to count *k*-mer frequency, and a histogram of *k*-mer distributions was generated. Jellyfish could be used on ONT read data because read trimming and error correction steps were completed manually. Peak coverage was taken to be the average *k*-mer coverage, and genome size was estimated by the following formula: G_size_ = *k*-mer_count_/Peak_position_, where the *k*-mer_count_ and Peak_position_ are the total number and the average depth of 21-mer and 25-mer combinations, respectively. ONT read error correction was performed using Canu (RRID:SCR_015880) correction and trimming modules [55].

### Assigning taxonomic labels to reads

To confirm Myxozoa identification based upon microscopic morphology and to check that the final datasets were free from host and other contaminating DNA, a search for both 16S rDNA and 18S rDNA taxonomic markers in raw and corrected reads was undertaken using the SILVA Release 138.1 nonredundant Small Subunit rRNA Database [56]. Hits to the dataset were assigned taxonomic labels using the all-species taxonomic framework function [57]. To assign taxonomic labels at a species level, a manual examination of alignment hits between the query reads and the SILVA database was performed to extract sequences that corresponded to 18S rDNA hypervariable regions. These regions were analyzed by BLASTn alignment against unpublished Myxozoa 18S rDNA sequences generated by our group and also against the entire nr nucleotide database at NCBI [58] to further confirm removal of contaminating sequences. Phylogenetic analysis was conducted on 678 Myxozoa 18S rRNA sequences, with 673 obtained from the Silva database [56] and 5 extracted from our genomes. The analysis was based on multiple sequence alignment, where all insert columns have been removed, including any consensus column which had fewer than 95% of the sequences that are nongaps and had a posterior probability of less than 0.95. This data preparatory process, which assumes all aligned nucleotides are homologous, ensures that the resulting phylogeny is based on robust data. The alignment and variable region masking were performed using SSU-ALIGN [59], and the phylogenomic inference, including tree reconstruction, was accomplished using IQ-TREE (RRID:SCR_017254) [60] installed on a Linux server. The built-in “ModelFinder” feature [61] has been used to determine the best-fit substitution model for 18S DNA multiple sequence alignment input. For the visualization of the resulting unrooted tree, iTol v6 (RRID:SCR_018174) was used [62].

### Assessment of genome assembly completeness and quality

The completeness and quality of each individual genome assembly was assessed separately and then compared against all 8 Myxozoa genomes currently available (Supplementary File 2) using QUAST (RRID:SCR_001228) [63] and BUSCO analyses (RRID:SCR_015008) [64]. The QUAST analyses provided technical metrics with which to compare the relative completeness of genomes, for example, numbers and sizes of contigs, G+C content, numbers of base mismatches, and overall quality in terms of N50, N90, L50, and L90 values. Genome quality was also assessed by measuring anticipated gene content using BUSCO analysis with a 255 single-copy ortholog dataset (Eukaryota Odb10) that is considered representative of genes that encode core biological functions in eukaryotes [65, 66]. To be included in the BUSCO set, genes had to be present as single-copy orthologs in at least 90% of the species within a major branch of the species phylogeny. Additionally, to ensure a diverse distribution across various taxa, the BUSCO dataset does not contain a single subclade where all the genes were missing. The BUSCO analyses were implemented using a docker container [67]. BUSCO analyses using the same Eukaryota Odb10 dataset provided standardized metrics that allowed comparative genome quality to be determined between the genomes assembled herein and those Myxozoa genomes already available in public databases.

### Methylation calling

DNA methylation signals were present in the fast5 files, which originated directly from the ONT sequencing cell (PromethION FLO-PRO002 [RRID:SCR_017987] with the SQK-LSK110 sequencing kit) and were also used for base-calling. We adopted Nanopolish v.0.14.0 (RRID:SCR_016157) [68], an analytic tool that uses a hidden Markov model to distinguish 5-mC from unmethylated cytosine directly from fast5 files, for 2 reasons. First, it uses an agnostic model approach to detect DNA methylation that does not require training data, which is a constraint when dealing with relatively unknown genomes. Second, the method has been shown to either outperform or provide equivalent results for methylation calling offered by other approaches [69] that are highly dependent on the training data. Since Nanopolish needs to access the signal-level data measured by the ONT sequencer, index files were created that linked long-read identifiers with the corresponding signal-level data in the fast5 files. Next, the base-called reads were aligned to each assembled genome contig, using the ONT specific “map-ont” option of minimap2. Nanopolish was then used to detect methylated bases (5-methylcytosine in CpG context) using the internal “call-methylation” function. The original Nanopolish output file contained much information, including identifiers, the position of the CG dinucleotide on genome contigs, and the log-likelihood ratio calculated by the model. A positive value in the log_lik_ratio column of the output files indicated support for methylation. By using a helper script called “calculate_methylation_frequency.py” accessible within the Nanopolish installation files, the methylation frequency for all detected CpG sites on each assembly contig could be calculated. These methylation frequency values range from 0 to 1, with zero indicating no support for methylation whatsoever and 1 indicating that all the sequence data covering a particular CpG site had the methylation signal. Each value placed between 0 and 1 boundaries represents the ratio of mapped sequence data giving a positive methylation signal for a particular CpG site.

### Gene finding, annotation, and genome comparisons

Because DNA material was isolated from endoparasites living within their hosts, MetaEuk v.6.a5d39d9 was used for gene discovery and annotation [70]. This toolset is designed specifically to handle large-scale datasets reflecting eukaryotic metagenomic contigs. It also integrates the rapid and sensitive homology search functionalities offered by MMseqs2 with a dynamic programming approach tailored for the retrieval of optimal exon sets [71]. For both gene annotation and taxonomic assignment, the latest UniProtKB database was used. The entire set of UniProtKB protein sequences was downloaded locally in FASTA format and converted to become our own customized baseline annotation database using the MMseqs2 “mmseqs” command. This procedure enabled us to augment the sequence database with additional taxonomic information using the sequence accession descriptions, thus linking our sequence data with taxonomic identity using an appropriate mapping file. The “easy-predict” MetaEuk workflow was used on all assembled genome contigs and scaffolds to predict protein coding sequences and respective proteins in a nonredundant manner. To complement the customized UniProtKB reference database, the entire current release of PANTHER HMM library (v.18) was also downloaded and installed locally for functional classification of proteins [72]. After obtaining gene predictions, the “taxtocontig” workflow in MetaEuk was used to assign taxonomic labels to the predicted proteins. The parameter “–majority” was used with this workflow, so that at least 50% of labeled predictions were used for positive taxonomic assignment. To integrate this workflow into our Myxozoa genomic pipeline (Fig. 1), several Python scripts were written for more efficient parallelization of search tasks, for PANTHER search output parsing, and for further downstream analysis. For example, the PANTHER HMM scoring tool was used to screen a hidden Markov model (HMM) library specifically for all protein families related to the nucleus-based Gene Ontology (GO) biological processes of base-excision repair (BER), DNMT, and DNA demethylation (ten-eleven translocation methylcytosine dioxygenase [TET]).

Word cloud analysis of GO terms linked to genes was used as a visualization strategy and aimed to enhance the interpretation of the vast and complex information the genome annotations yielded. Keywords and key phrases were extracted from the textual descriptions of gene functions using a state-of-the-art keyword extraction tool KeyBERT [73]. The extracted terms were next visualized using word cloud, which provides a powerful tool for representing the relative importance of terms through graphical representation [74].

### Determining cytosine methylation at CpG sites within and outside protein coding sequences (CDSs)

The “taxtocontig” and “–lca-mode” function of MetaEuk was used to predict the taxonomy of CDS and non-CDS sequences. The additional parameter “–majority” was also used with this workflow. All predictions were based on candidate sequence alignment to the UniProtKB database used for genome annotation. Alongside this pipeline, a set of Python scripts and programs was written utilizing BioPython (RRID:SCR_007173) [75] modules to parse the gene prediction output files and to link these predictions to other layers of information. The average G+C content of CDS and non-CDS sequences was then calculated based on predicted sequence boundaries within each genome assembly. Knowing the G+C content, the observed/expected CpG ratio (CpG O/E) for each CDS and non-CDS sequence in each genome could be calculated using the following equation:


\begin{eqnarray*}
CpG\ O/E\ = \ \frac{{CG\ x\ l}}{{C\ x\ G}}
\end{eqnarray*}

where “l” is the sequence length, “C” denotes the number of cytosine residues, and “G” is the number of guanine residues within a sequence. A Python implementation of a Gaussian mixture model (GMM) called “MethMod” [76] was used to predict the presence of DNA methylation based on observed differences between CpG rates in CDSs extracted from genome annotations [70]. The GMM had 2 components and calculated the Akaike information criterion (AIC) alongside the statistical mean of each component. The distance between the statistical mean of each component and the relative amount of data points in each component were also calculated (i.e., the percentage). To characterize the distributions of each Myxozoa genome CDS CpG O/E values, the distribution means, sample standard deviations, and the skewness were also calculated. To report only the reliable “MethMod” calculations, shuffled data were used as a control. The most informative “MethMod” value calculated is the distance between the component means, which serves as an indicator for DNA methylation. If the distance is greater than or equal to 0.25, it can be assumed that DNA methylation is present; otherwise, it is more likely that DNA methylation is absent [77].

### Statistics used for validation

To determine if the number of CpG sites within a CDS was correlated to the length of the CDS (in bp), the “Matplotlib” and “NumPy” modules in BioPython were used to calculate a Pearson correlation coefficient. The lengths and the observed number of CpGs for each CDS in the 3 genome assemblies were plotted, and a linear least squares regression was used to model the relationship between the number of CpG sites and CDS length. A kernel density estimation (KDE) was used to further validate the “MethMod” results and to justify the application of this method on our Myxozoa assemblies. It is important to notice that the GMM used in MethMod is a parametric probability density function represented as a weighted sum of Gaussian component densities. As such, it has to make an underlying assumption on the number of components. In the case of DNA methylation, 2 components are a natural choice accounting for methylated and unmethylated genes. However, in view of the rapid evolution that characterizes myxozoans, the nonuniform myxozoan genome assemblies, and the Kyger et al. [10] results indicating absence of methylation, an additional nonparametric method (KDE) was used to validate the parametric GMM test results.

KDE applies kernel smoothing for probability density estimation and represents a nonparametric method often used to estimate the probability density function of a random variable based on kernels as weights. Testing different kernels for KDE is important because the choice of kernel can have a significant impact on the estimated probability density function. Three were selected, and they were the “Epanechnikov,” “tophat,” and “Gaussian” kernels, with Gaussian providing the closest estimate. A code to generate KDE was written using “NumPy,” “Matplotlib,” “SciPy,” and “scikit-learn” modules of BioPython. The code resulted in bimodal distributions based upon the kernel density estimates, fitted to the ONT Nanopore predicted myxozoan CDS CpG O/E data. To determine the exact location of each methylated base (i.e., 5-methylcytosine in a CpG context) for each assembled Myxozoa genome, the fast5 datasets were processed using the same Nanopolish, Guppy, and minimap2 pipelines described previously. To demonstrate the inverse correlation between calculated CDS CpG O/E and experimentally recorded CpG methylation frequencies within the respective CDS gene bodies, a scatterplot was drawn. This scatterplot included a group of 1,000 CDSs characterized by highest calculated CpG O/E ratios and another group consisting of the same number of CDSs with the lowest CpG O/E ratios in the dataset. On the y-axis for each CDS group, a cumulative methylation frequency (expressed as an average) was calculated by summing all respective CpG site methylation frequencies and dividing this sum by the number of CpG sites for each of the selected CDSs. To smooth the methylation frequency plot and to make a more informative figure, a Savitzky–Golay filter was implemented from the “SciPy” module of BioPython. An elbow method was additionally employed to independently assess the optimal number of clusters (e.g., distributions, number of components) according to CDS CpG O/E values. To go even further and validate these expectations using experimental data obtained by ONT sequencing, the KDE analysis was repeated and ONT Nanopolish-derived methylation frequencies were introduced, which were calculated for each CpG site within CDSs in its full range (floating point value from 0–1 range) as function weights. Thus, in this weighted KDE approach, an entire methylation frequency floating point scale from 0 to 1 was utilized, allowing us to pinpoint CpG O/E values that are best at separating methylated versus nonmethylated CDSs. Since CpG O/E values can be calculated solely based on genomic sequences, this is a very useful method for proving the existence of methylation in genes and to provide the accurate boundaries separating methylated from nonmethylated genes within genomes. All figures were drawn using “matplotlib” within the Anaconda environment (RRID:SCR_025572) [78] and using JupyterLab (RRID:SCR_023339) [79].

## Results

### Identification of Myxozoa

We detected myxozoan infections in 102 of 189 fish gallbladders (54%). The DNA of myxozoans collected from the bile of 5 fish specimens was isolated in sufficient concentration and purity required for ONT sequencing. Just over 9 terabytes of sequencing data were generated. To assign taxonomic labels to the Myxozoa, both raw and corrected reads were used as inputs for identification of 16S and 18S rDNA sequences by comparison to SILVA Release 138.1. Table 1 summarizes the material studied (FASTA sequences are provided in Supplementary File 3). Light microscopy at low magnification without oil (typically 20× or 40×) was also used to identify myxozoan infections and to tentatively identify the myxozoans to the genus level based on morphology (Table 1). Photographs of the Myxozoa and host fish are provided in Supplementary File 4 for 4 of the 5 bile samples listed in Table 1. Closer inspection of the *Ceratomyxa* spp. isolated from fish samples 57 and 58 (*Plagioscion squamosissimus*) revealed cell morphologies reflecting different stages of development (Supplementary File 4, Fig. S1). These ranged from immature to mature plasmodia, the latter containing numerous spores. Advanced sporogenesis stages were predominant. The identification of plasmodia and myxospores was associated with hits to 18S sequences of myxozoans in the genus *Ceratomyxa* in the SILVA database. The inferred identification of Myxozoa found in the bile from fish sample 70 (*Curimata inornata*) was inconsistent between the molecular and microscopic data. Molecular data indicated a closest database match to *Ceratomyxa*. However, the morphologies of parasite stages in the bile (many immature plasmodia, some mature plasmodia with myxospores, and some free myxospores) were clearly those of *Ellipsomyxa* (Supplementary File 4, Fig. S2). Two morphological types of Myxozoa were observed in the bile collected from sample 115 (*Hemiodus unimaculatus*). These were mainly *Ceratomyxa* plasmodia at different levels of sporogenesis and free myxospores but also some stages consistent with the morphology of immature *Ellipsomyxa* plasmodia without myxospores (Supplementary File 4, Fig. S3). It would appear that *Ellipsomyxa* DNA was sequenced from sample 115 as only sequences homologous to *Ellipsomyxa* were matched in the SILVA database. Sequences homologous to infection by *Ellipsomyxa* and *Myxidium* were retrieved in the SILVA database for sample 108 (*Rhaphiodon vulpinus*; a photo of the fish is given in Supplementary File 4, Fig. S4). Photographic evidence was not obtained for Myxozoa in this sample, but *Ceratomyxa* sp. was identified by microscopic examination and recorded in the field notebook.

**Table 1:** Putative identification of Myxozoa. Identification was made using sequencing read comparison against an 18S rDNA database (SILVA) and visual analysis of morphological features using light microscopy.

Specimen	Host	Identification based on best hit to the SILVA database	Identification based on microscopic morphology
57	*PIagioscion squamosissimus*	*Ceratomyxa*	*Ceratomyxa*
58	*PIagioscion squamosissimus*	*Ceratomyxa*	*Ceratomyxa*
70	*Curimata inornata*	*Ceratomyxa*	*Ellipsomyxa*
108	*Rhaphiodon vulpinus*	*Ellipsomyxa* and *Myxidium*	*Ceratomyxa*
115	*Hemiodus unimaculatus*	*Ellipsomyxa*	*Ellipsomyxa and Ceratomyxa*

**Figure 2:**
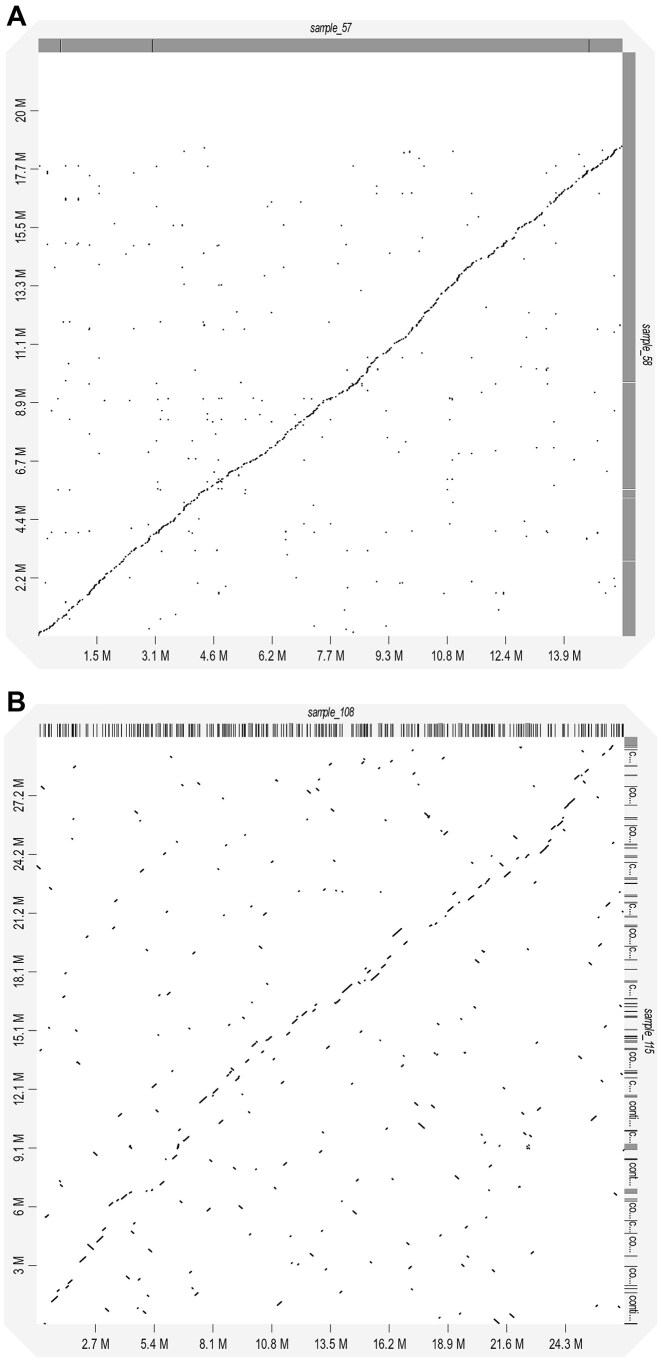
Whole-genome dot-plot comparisons of (A) samples 57 and 58 and (B) samples 108 and 115. Linearity implied a common ancestry with breaks in the plot indicative of genome fragmentation or deletions. More fragmented diagonal with a higher density of lines off the main diagonal suggests that samples 108 and 115 (B) are less related in comparison with samples 57 and 58 (A), which we believe to represent the same species.

To confirm species identification, the identified 18S FASTA sequences (Supplementary File 3) were analyzed manually for hypervariable regions (Supplementary File 5). Subsequent phylogenetic analysis revealed no overlap of 18S sequences with those of existing myxozoan sequences deposited in the SILVA database (Supplementary File 6A provides a phylogenetic tree where our samples are labeled in red. The phylogeny is also represented in Newick format in Supplementary File 6B). Very recently published Canu built-in methods that handle the typically high read error rates linked with nanopore sequencing were employed for error correction and read trimming to provide more reliable 18S rRNA gene analysis [80]. However, no convincing homology alignments could be made with known myxozoan species. In addition, a pairwise assessment of genome synteny was performed using ONT-generated polished assemblies to determine if any of the samples shared identity. Using standalone D-Genies [81] to display the synteny outputs, it was possible to globally align samples 57 to 58 (Fig. 2A) and samples 108 to 115 (Fig. 2B). Such synteny further strengthened the previous 18S rDNA-based taxonomic assignments (Table 1), which, together with the annotation results reported below, strongly support the identity of myxozoans in samples 57 and 58. These were likely the same *Ceratomyxa* species isolated from different specimens of the same fish species. The synteny result also supported the identity of myxozoans in samples 108 and 115, these being *Ellipsomyxa* isolated from 2 different fish species. However, later comparisons between the functional annotations of genes would suggest a clear taxonomic difference between samples 108 and 115. In the absence of photographic evidence, sample 108 was considered a myxosporean species and is referred to as Myxosporea sp.

### Myxozoa assembly comparisons

*Ceratomyxa* sp. sample 57 and *Ceratomyxa* sp. sample 58 both contained large numbers of myxospores (Supplementary File 4, Fig. S1) but only provided partial genome assemblies and were discounted from further genomic comparisons. ONT read assembly provided 3 high-quality draft Myxozoa genomes: for *Ellipsomyxa* sp. sample 70, Myxosporea sp. sample 108, and *Ellipsomyxa* sp. sample 115. Overall, this produced satisfactory results despite the initial imbalance of reads ranging from 10 to 1 in favor of host DNA in *Ellipsomyxa* sp. sample 115 and up to 4 to 1 in favor of host DNA in *Ceratomyxa* sp. sample 58. The only sample that had relatively more parasite reads compared to the host was *Ellipsomyxa* sp. sample 70, which revealed an approximately 2 to 1 ratio in favor of parasite DNA reads. These calculations were based on ratios between excluded reads (i.e., ones that mapped onto reference fish genomes) and reads that mapped onto contamination pruned assemblies. The 8 Myxozoa genome assemblies currently available in public databases were all assembled from reads generated using various Illumina sequencing platforms (Supplementary File 2). QUAST analysis clearly demonstrated that the 3 genomes generated from samples *Ellipsomyxa* sp. sample 70, Myxosporea sp. sample 108, and *Ellipsomyxa* sp. sample 115 had superior overall quality to the 8 published genomes in terms of best N50, N90, L50, and L90 values and were also completely free of mismatches (Supplementary File 7). The estimated genome sizes were in the 20 to 30 Mbp range. These estimated genome sizes were further confirmed by Jellyfish *k*-mer analysis, which revealed total genome lengths for *Ellipsomyxa* sp. sample 70, Myxosporea sp. sample 108, and sample 115 as 24.26, 26.97, and 30.22 Mbp, respectively. Our estimated total genome sizes are comparable to the 31.2 Mbp genome size estimated for *Kudoa iwatai* but far smaller than the predicted genome sizes for the other 7 previously sequenced myxozoans, which range from 61.44 to 234.48 Mbp (Supplementary File 7).

**Figure 3:**
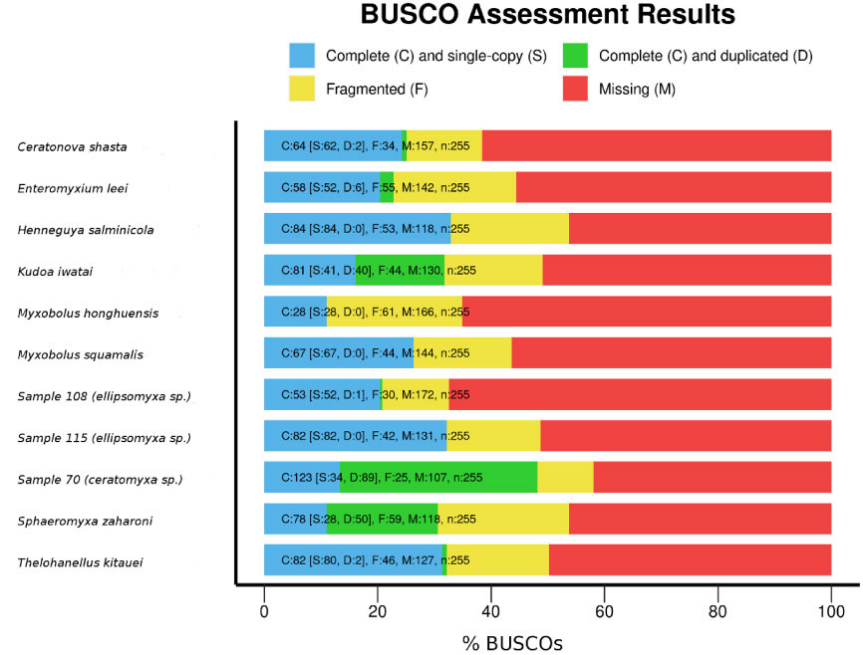
BUSCO assessment of genome assembly and annotation completeness for the 11 myxozoan genomes presently available. Despite a low level of shared BUSCOs overall, there was a small consensus of core proteins shared between the genomes.

The QUAST comparison only provided a technical assessment of the genome assemblies, with no regard to functional aspects or genome completeness. To compare the ONT assembly more thoroughly to the previously generated Myxozoa assemblies that were all generated by Illumina platforms, a BUSCO-based assessment was performed (Fig. 3). This assessment included the entire dataset of 11 Myxozoa genome assemblies. There were 163 complete and unique BUSCOs identified from a total of 255 eukaryotic genes that comprised the BUSCO Eukaryota Odb10 dataset (63.9%). A reduced set of BUSCO genes is expected given the observed trend in parasite genome reduction. However, there were only 6 (2.4%) BUSCOs shared by all the genomes assessed. The highest-quality genome assembly belonged to *Ellipsomyxa* sp. sample 70, which scored a total of 123 complete and duplicated BUSCOs. This represented 75.5% of the overall 163 BUSCOs identified. All other assemblies shared a majority of identified complete BUSCOs with this assembled sample 70 genome (ranging from a minimum of 68% shared with *Myxobolus honghuensis* to 90% shared with Myxosporea sp. sample 108 and with *Enteromyxium leei*). When the initial ONT fastq reads of *Ellipsomyxa* sp. sample 70 were mapped onto the final polished version of the assembly (Supplementary File 8A), the median genome coverage was 1,424. Using both complete and duplicated single-copy genes (BUSCOs) to assess the expected coverage more precisely, an overall sufficient level of coverage has been confirmed, and a relatively high level of gene duplication indicated by BUSCO analysis has further been emphasized when average BUSCO gene coverage has been calculated (Supplementary File 8B). Regarding the expected genome coverage, a histogram accompanied by probability density function that summarizes distribution of this genome assembly coverage has revealed 2 distinct peaks that completely correlate with BUSCO gene coverage, indicating large-scale genome duplications (Supplementary File 8C). It was therefore reasonable to consider *Ellipsomyxa* sp. sample 70 as the best-quality myxozoan genome assembly reported to date. The overall low number of shared BUSCOs between *Ellipsomyxa* sp. sample 70 and the other assembled genomes could be attributed to incompleteness, excessive fragmentation, contamination (including coinfections of myxozoan species, a scenario that may partly explain our identification incongruities and could contribute to variation in qualities of assemblies), or a combination of these factors (Supplementary File 7). Even though a high-level eukaryote BUSCO set was used, a notable proportion of BUSCOs detected were duplicates, which was also reflected at the level of total genome coverage (Supplementary File 8). The evolutionary significance of apparent nonrandom, ordered, and regular gene duplication could have significant implications beyond the scope of this article but warrants future experimental investigation (Supplementary File 8). These ranged from 0% to 72% of all identified complete BUSCOs in the assembled genomes (Fig. 3). This result is striking given the ubiquitous underlying basis of the BUSCO set (Eukaryota Odb10) and that the genomes are reduced in size (Supplementary File 7). The unusually low level of BUSCOs shared among these myxozoan taxa also suggests that a small consensus of core proteins may be essential for endoparasites with reduced genomes. This was explored further when the genomes were annotated and is described below.

### Gene annotation and genome analysis

**Figure 4:**
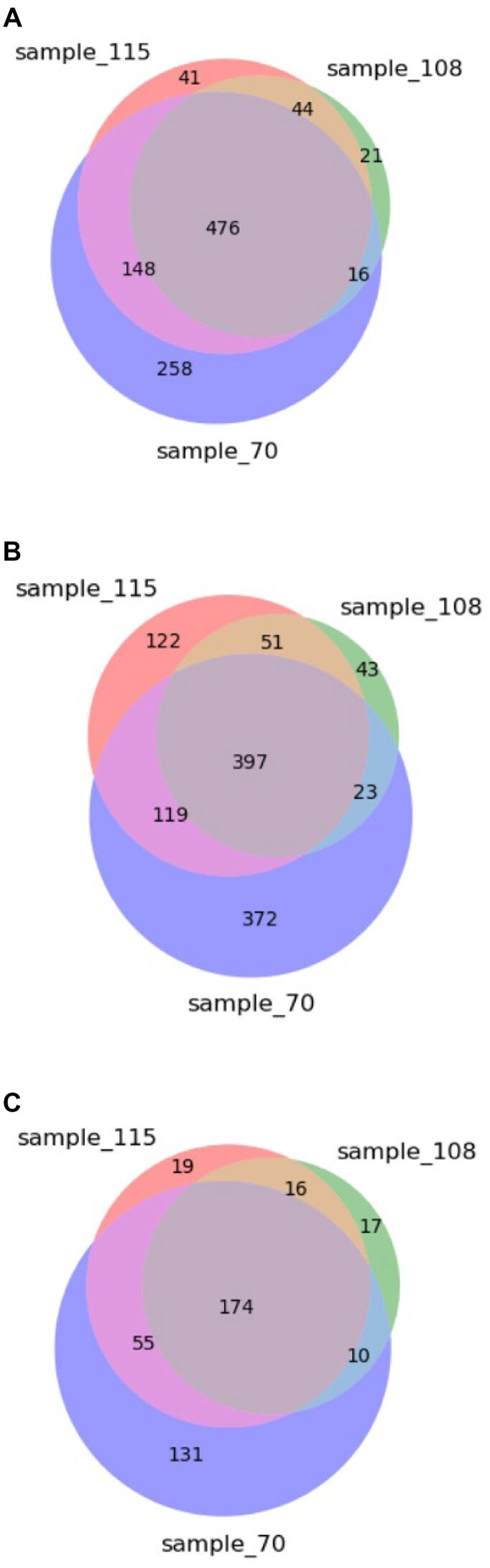
Comparison of the number of annotated genes shared between samples 70, 108, and 115. Euler–Venn diagrams display the number of unique genes annotated by GO terms for (A) molecular function, (B) biological process, and (C) cellular component.

**Figure 5:**
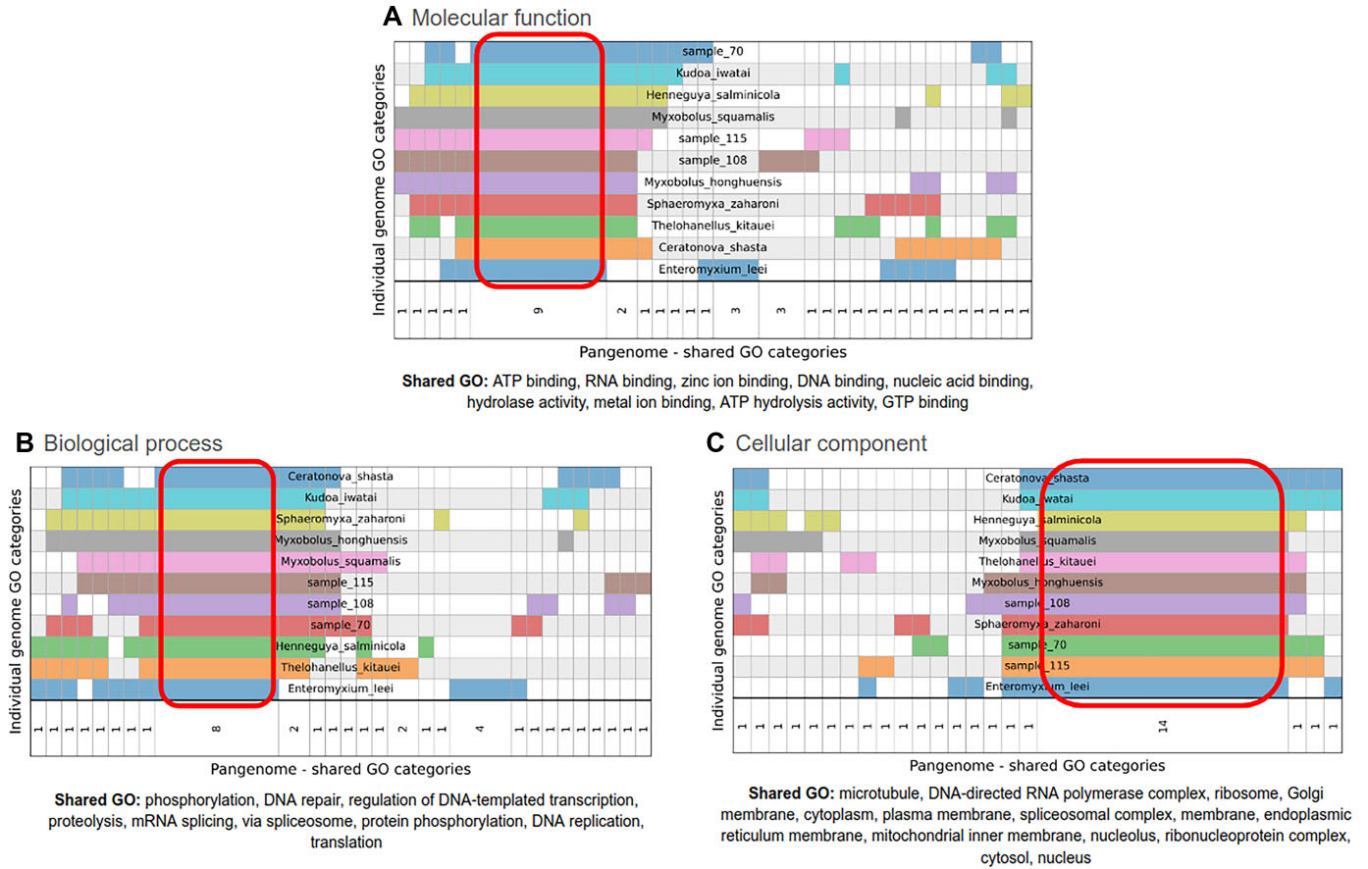
Pangenome annotation comparison between the 8 Myxozoa genomes publicly available and the 3 assemblies produced in this study. This pangenonne comparison shows that on the top level of GO annotations, Myxozoa do possess a conserved core gene repertoire (demarcated within the red lines).

Comparisons of the gene annotations for ONT sequenced *Ellipsomyxa* sp. sample 70, Myxosporea sp. sample 108, and *Ellipsomyxa* sp. sample 115 are shown in Fig. 4. The Euler–Venn diagrams reveal that most genes are common to all 3 genomes, irrespective of the level of GO annotation used (molecular function, biological process, or cellular component). Although the level of unique functions, processes, or components shared between Myxosporea sp. sample 108 and *Ellipsomyxa* sp. sample 115 was rather small (between 4% and 10%), the genome annotations were not completely identical. This further supported variation in 18S rDNA sequences, genome synteny, microscopic morphology, and BUSCO analyses that suggested Myxosporea sp. sample 108 and *Ellipsomyxa* sp. sample 115 were different taxa. A genome analysis revealed a core gene repertoire that contained 39,140 gene variants (76.83% of all genes in the top 20 GO terms) falling within molecular function GO process, 8,339 genes annotated with a biological function (43.80% of all genes in the top 20 GO terms), and 23,186 genes assigned to a cellular component (92.60% of all genes in the top 20 GO terms). Given the large number of genes that share predicted coding functions among all 11 myxozoan genomes, we used word cloud analysis to decipher what protein functions were overrepresented within the core gene repertoire (Supplementary File 9). Key words and key terms could then be more easily distinguished from the word clouds (see Methods), and these are shown in Table 2. In summary, when *Ellipsomyxa* sp. sample 70 was used as a reference genome, both BUSCO (Fig. 3) and genome analysis (Fig. 5, Table 2) concurred that the same 20 most abundant GO terms were common to *Ellipsomyxa* sp. sample 70 and all the other 10 assemblies (i.e., Myxosporea sp. sample 108 and *Ellipsomyxa* sp. sample 115, as well as the 8 previously published myxozoan genomes). These data support that myxozoans possess a conserved core function gene repertoire, perhaps essential for endoparasites with reduced genomes, which was not immediately evident when relying solely on gene homology.

**Table 2:** Predicted protein functions by GO process encoded by a core repertoire of genes shared among 11 myxozoan genomes. Use of Named Entity Recognition (NER) in terms of keyword recognition and GO term analysis visualized using word cloud methods enabled initial identification of gene categories with further analyses as described in the Methods section. The terms being shared are not necessarily the most abundant GO categories in each individual genome annotation and word cloud depictions.

GO Process	Shared GO terms
Molecular Function	GTP binding, nucleic acid binding, RNA binding, metal ion binding, hydrolase activity, ATP binding, DNA binding, ATP hydrolysis activity, zinc ion binding
Biological Process	DNA repair, proteolysis, protein phosphorylation, mRNA splicing via spliceosome, regulation of DNA-templated transcription, DNA replication, translation, phosphorylation
Cellular Component	mitochondrial inner membrane, Golgi membrane, DNA-directed RNA polymerase complex, endoplasmic reticulum membrane, nucleolus, plasma membrane, ribosome, microtubule, nucleus, spliceosomal complex, ribonucleoprotein complex, cytoplasm, cytosol, membrane

### ONT nanopore methylation calling

**Figure 6:**
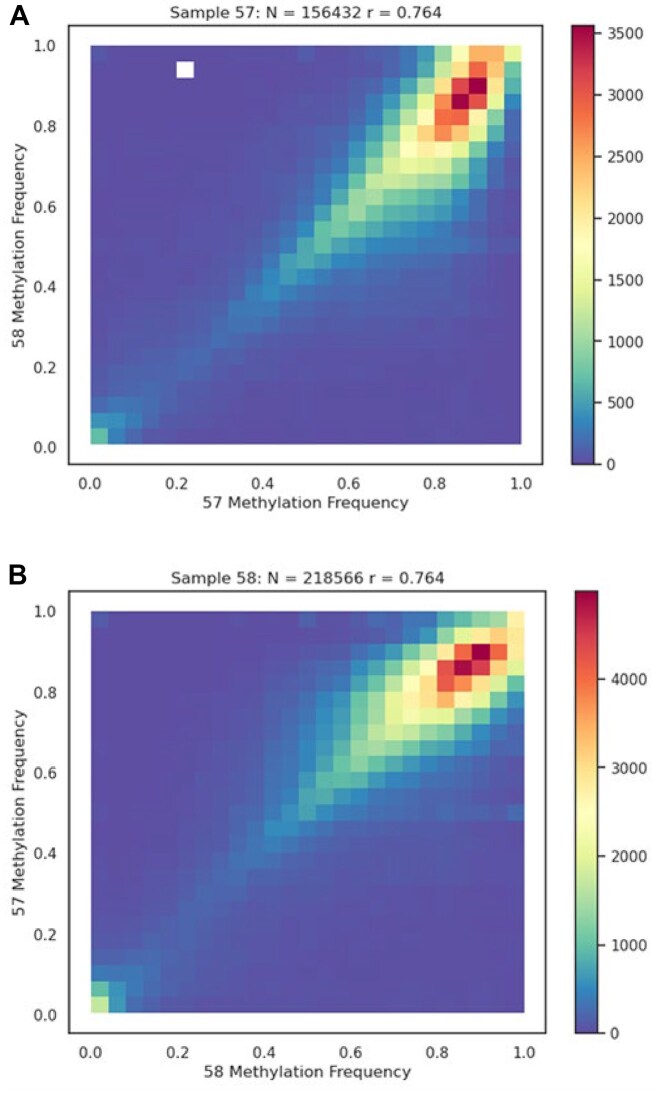
Bidirectional methylation calling comparing samples 57 and 58 to validate the ONT methylation calling. Heatmap (A) compares sample 57 as the signal-level data against sample 58 as the biological replicate; (B) is the reverse.

The sequencing reads for *Ceratomyxa* spp. isolated from fish samples 57 and 58 could only be assembled as partial genomes. However, these genomes displayed close to total synteny (Fig. 2A). This, along with similar gene annotation results, indicated the same *Ceratomyxa* species was present in the bile of individuals of the same fish species that were caught at the same time. These partial genomes can thus be considered independent biological replicates with which the Nanopolish DNA methylation calling can be assessed. The sample methylation cross-comparison (bidirectional methylation calling) of *Ceratomyxa* sp. sample 57 and *Ceratomyxa* sp. sample 58 in both directions validated the ONT methylation calling results obtained using Nanopolish for each sample independently. These analyses also provided further support for the same species of *Ceratomyxa* in samples 57 and 58. Fig. 6A is a heatmap for the results of such a cross-comparison analysis using *Ceratomyxa* sp. sample 57 signal-level data mapped against *Ceratomyxa* sp. sample 58 genome assembly data as the biological replicate. Fig. 6B is the reverse result whereby sample *Ceratomyxa* sp. sample 58 signal-level data were mapped against *Ceratomyxa* sp. sample 57 genome assembly data as the biological replicate. The heatmaps obtained by grouping comparable methylation frequencies across all CpG sites in the 2 sample assemblies indicated 2 things. First, the correlation was very strong (Pearson correlation coefficient as a measure of the linear relationship between 2 variables = 0.764 in both sample comparisons), validating the ONT methylation calling without the necessity of employing bisulfite sequencing. Second, most of the recorded methylation frequencies fell in the range between 0.8 and 1. This provides strong evidence that most of the assembled genome CpG sites were methylated and thus that most of the genes in these genomes were likely to be transcriptionally silenced at the time of sampling.

**Figure 7:**
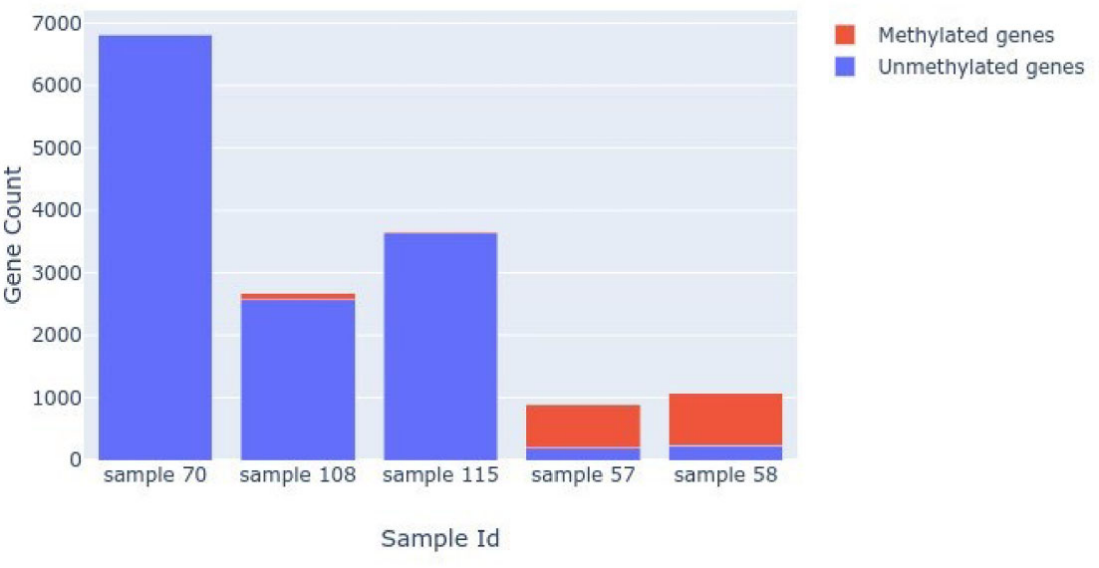
Methylated versus unmethylated gene frequencies for the 5 assemblies relative to genome size (according to number of genes). Most genes in samples 57 and 58 were methylated and hence transcriptionally inactive. In samples 70, 108, and 115, most genes were not methylated (i.e., the unmethylated frequencies fell below 0.3) and hence being expressed.

The cross-comparison additionally provided a procedure to determine the quality and accuracy of the sequence assemblies and to identify conserved regions or orthologous genes between the samples. The methylation frequencies and associated gene counts for assemblies (genome size estimates) of *Ceratomyxa* sp. sample 57, *Ceratomyxa* sp. sample 58, *Ellipsomyxa* sp. sample 70, Myxosporea sp. sample 108, and *Ellipsomyxa* sp. sample 115 are displayed as a bar chart (Fig. 7). The methylation frequencies for assemblies obtained from *Ellipsomyxa* sp. sample 70, Myxosporea sp. sample 108, and *Ellipsomyxa* sp. sample 115 are in striking contrast to those from samples *Ceratomyxa* sp. sample 57 and *Ceratomyxa* sp. sample 58. This surprising result indicates that most of the genes in the assemblies deriving from *Ellipsomyxa* sp. sample 70, Myxosporea sp. sample 108, and *Ellipsomyxa* sp. sample 115 were likely not methylated. Figure 7 also illustrates that the *Ellipsomyxa* sp. sample 70 assembly is arguably the cleanest and most accurate myxozoan genome yet described. This is supported by the genome assembly analyses (the QUAST technical assessment of the genome assemblies [Supplementary File 7] and BUSCO evaluation of genome completeness [Fig. 3]).

### Distribution of DNA cytosine methylation proteins

To rigorously assess the feasibility of CpG methylation capabilities of myxozoans, all 8 Myxozoa genomes currently available and the additional 5 ONT-based assemblies produced herein were all annotated using the same bioinformatics pipeline (Fig. 1). Table 3 summarizes the presence of the most relevant mechanisms supporting molecular methylation in these various myxozoan genomes identified by the results of this uniform annotation procedure. These include DNMT, TET, and BER enzymes. Five myxozoan genomes encoded all 3 key methylation components necessary for successful CpG methylation/demethylation and subsequent base excision repair (*M. honghuensis, Ceratonova shasta, Ellipsomyxa* sp. sample 70, *Ellipsomyxa* sp. sample 115, and *Henneguya salminicola*). The remaining 7 genomes were missing either TET or DNMT components. BER was the dominant methylation enzyme in all the genomes, and more than 1 copy was present in all genomes analyzed. BER was absent in the partial genome assemblies of *Ceratomyxa* sp. sample 57 and *Ceratomyxa* sp. sample 58, both of which were also characterized by missing genetic information. The most important enzyme class for inferring CpG methylation is DNMT. DNMT was the second most abundant methylation mechanism, being present in 8 genomes, including *Ceratomyxa* sp. sample 57, which provided strong evidence for extensive CpG methylation (Fig. 7). TET was the third most abundant methylation enzyme and had protein family representatives in over 50% of analyzed genomes.

**Table 3:**
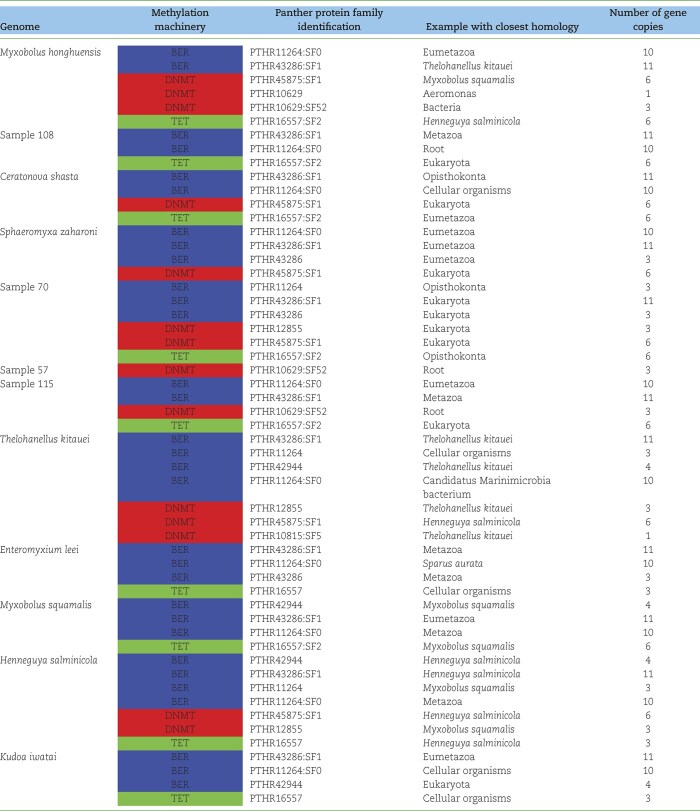
Distribution of GO biological processes of BER, DNMT, and TET across 13 myxozoan genome assemblies. The presence of any genes falling into these 3 categories is denoted with a different color: red (DNMT), blue (BER), and green (TET). Exact protein family members identified are labeled with Panther IDs in the third column, and taxonomic assignments of identified proteins based on homology are given in the fourth column. The fifth column provides the distribution of identified protein family members across 12 Myxozoa genome annotations (ranging from 1 = unique to a genome to 12 = appearing in all genomes).

### Distribution of CpG sites within and outside CDSs (protein coding sequences)

In general, there is a positive correlation between gene GC content and the number of CpG sites in a gene. This means that genes with higher GC content tend to have more CpG sites and vice versa. A calculation of G+C content of the genomes presented herein (Table 4) shows that myxozoan species have genomes ranging in G+C content as low as 16.71% in the case of *M. honghuensis* to 51.19% in *Ellipsomyxa* sp. sample 70. The myxozoan genome assemblies were divided into CDS and non-CDS sequences. Table 4 shows that consistently in all genomes analyzed, the CDS regions are characterized by increased GC content accompanied by more CpG sites when compared to non-CDS regions. For illustration purposes, we have made a visualization of this using 2 sample 70 contigs, where one can clearly observe higher levels of GC content in CDS regions of the genome (Supplementary File 10A). The ratio of observed/expected number of CpG sites serves as an indirect indicator of DNA methylation (this ratio is often used as a measure of CpG density where values <1 indicate a depletion of CpG dinucleotides commonly associated with active methylation and values >1 suggest an enrichment associated with genomic regions that are typically unmethylated). Based upon this assumption, Python-implemented GMM was conducted on all assembly CDS-calculated CpG O/E data. Component mean distances ≥0.25 were interpreted as indicative of DNA methylation in the modeling results [70]. This stringent threshold was selected to exclude false positives. The analysis indicated that 9 of 13 myxozoans are predicted to have methylation capabilities (Tables 3 and 4). There were 2 borderline cases, with component mean distances of 0.247 and 0.2316. *Ellipsomyxa* sp. sample 70, *Ellipsomyxa* sp. sample 115, and Myxosporea sp. sample 108 and *Thelohanellus kitauei* genomes have a value below 0.25 but still display slightly smaller average CpG O/E values in CDS genome sequences in comparison to non-CDS sequences, although the opposite was revealed for GC content. This indicated that even in these genomes associated with a very low degree of experimentally recorded methylation (e.g., *Ellipsomyxa* sp. sample 70; Fig. 7), there was depletion of CpG in coding sequences, and this could also be an indicator of methylation.

**Table 4:** Average G+C content of genome, CDS, and non-CDS regions together with CpG sites calculated in 13 Myxozoa genome assemblies. Final column contains the distance (d) between the component means obtained using the GMM of a CpG observed/expected value distribution. Values of d ≥ 0.25 can be used to infer the presence of DNA methylation (marked in red).

Species	genome GC	CDS GC	CDS CpG O/E	Non-CDS GC	Non-CDS CpG O/E	CpG O/E means distance (d)
Sample 58	42.17%	44.34%	0.36	42.01%	0.34	$\color{red}0.3116$
Sample 57	42.16%	44.54%	0.35	41.94%	0.39	$\color{red}0.3424$
Sample 70	39.39%	51.19%	1.04	35.86%	1.04	0.1359
*Enteromyxium leei*	33.51%	42.29%	0.58	30.92%	0.49	$\color{red}0.3298$
Sample 108	29.95%	32.20%	0.63	29.64%	0.70	0.247
Sample 115	29.95%	32.21%	0.65	29.44%	0.70	$\color{red}0.2535$
*Henneguya salminicola*	28.96%	33.25%	0.98	28.98%	1.07	$\color{red}0.2838$
*Sphaeromyxa zaharoni*	28.02%	35.09%	0.78	28.41%	0.91	0.2316
*Myxobolus squamalis*	27.30%	34.21%	0.84	28.40%	0.88	$\color{red}0.2939$
*Thelohanellus kitauei*	25.50%	35.06%	1.00	23.45%	1.34	0.2098
*Ceratonova shasta*	23.79%	31.08%	0.75	23.44%	0.87	$\color{red}0.3271$
*Kudoa iwatai*	23.64%	29.60%	0.54	23.08%	0.68	$\color{red}0.3073$
*Myxobolus honghuensis*	16.71%	31.49%	1.18	17.37%	1.68	$\color{red}0.4356$

**Figure 8:**
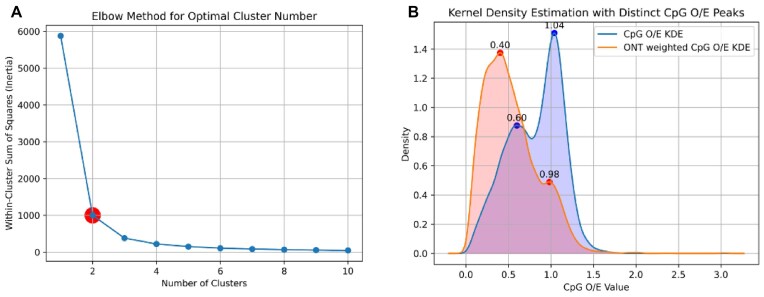
Kernel density estimation of methylation of CpG sites in CDS regions. (A) The elbow method determines the optimal number of clusters in the dataset. The inertia curve starts to flatten at a position indicating there are 2 clusters in this dataset. (B) Unweighted (blue) and methylation frequency weighted Gaussian KDE (orange) probability density function curves with distinct peaks denoting 2 clusters representing methylated and unmethylated portions of the genomes.

Our Python implementation of a nonparametric KDE method revealed a striking pattern of bimodal methylation distribution across the myxozoan CDSs, further confirming the existence of 2 distinct groups of CDSs with differential methylation patterns (Fig. 8A) and MethMod results already indicated. These results confirm the parametric MethMod results. This bimodality suggested the presence of 2 distinct populations of coding sequences based on CpG O/E values, potentially reflecting different methylation states and/or biological processes (Fig. 8B). The resulting probability density function curve (Fig. 8B, shown in blue) clearly indicates 2 distinct peaks corresponding to the underlying distributions of CDSs: 1 peak with an CpG O/E of 0.60 and another of 1.04. The lower CpG O/E peak was expected to represent a methylated cluster of CDSs based upon the presumption that methylation causes CpG depletion, while the higher CpG O/E value represented the unmethylated cluster of CDSs. The weighted KDE probability density function curve (Fig. 8B, shown in orange) also shows that the nonmethylated portion of myxozoan genomes is not being affected by the addition of ONT weights, since the peak of this cluster changes only marginally (from 1.04 to 0.98 CpG O/E). However, since genes that experimentally show signs of methylation have cumulative CpG site methylation frequencies that add significantly more weight, a much more dramatic effect on this cluster peak is observed (CpG O/E peak drops from 0.60 to 0.40). The underlying probability density function curve displays a reciprocal relationship between CpG O/E and ONT recorded methylation frequency when the weighted (orange) and unweighted (blue) curves are compared (Fig. 8B), with a clear increase in density of the methylated cluster and a proportional decrease in the nonmethylated cluster.

**Figure 9:**
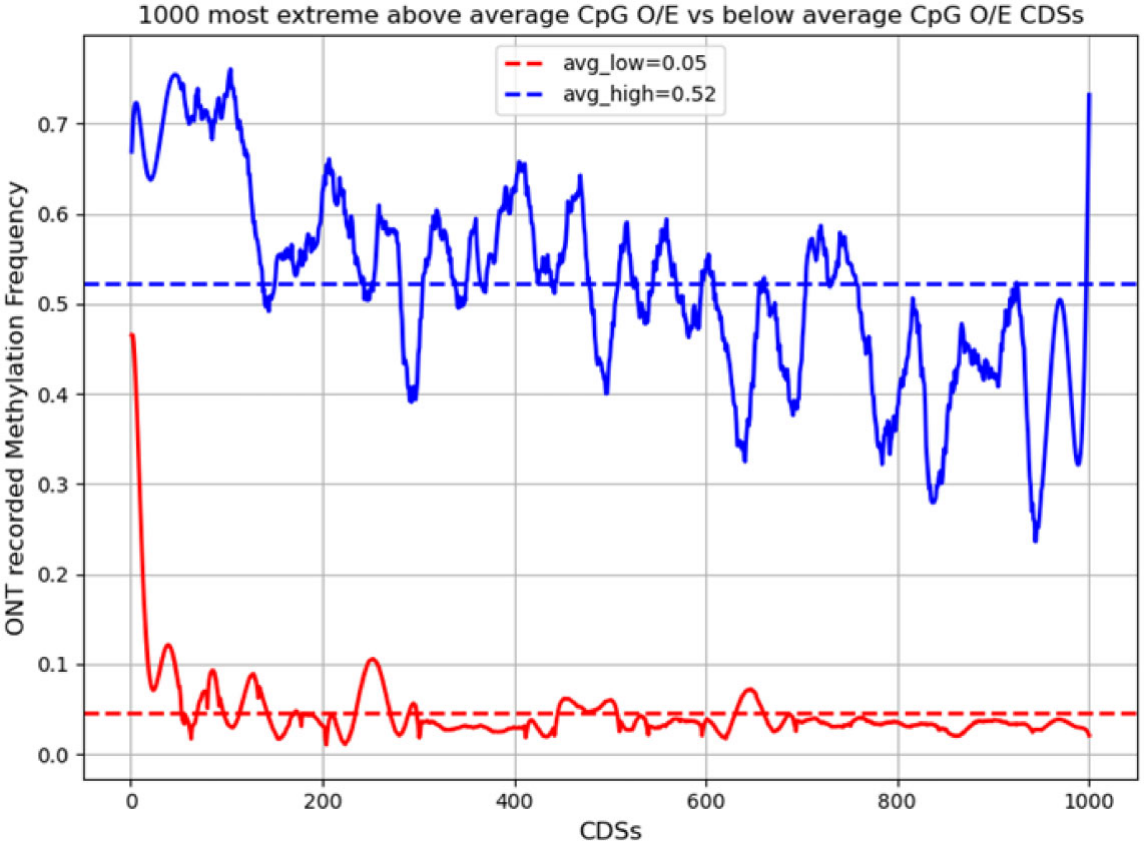
Correlation between CpG O/E values for CDSs as an indicator for CpG methylation. Savitzky–Golay filter smoothed plot displaying 1,000 lowest CpG O/E CDS cumulative CpG methylation frequencies (red) and 1,000 highest calculated CpG O/E CDS cumulative methylation frequencies (blue). Average values for each set methylation frequency is displayed with a dotted line, with the value reported in the plot legend.

The cumulative methylation frequencies (based on experimental data obtained by ONT-based Nanopolish methylation calling results) of the 1,000 most extreme CDSs with above-average CpG O/E (marked “high” and shown in blue in Fig. 9) and 1,000 most extreme below-average CpG O/E CDSs (marked “low” and shown in red in Fig. 9) are displayed on a smoothed plot (Fig. 9). The 1,000 below-average CpG O/E CDSs had a distinctly higher average methylation frequency (avg_low = 0.52), while the 1,000 CDSs with higher average CpG O/E had 11.2 times lower methylation frequency (avg_high = 0.05). These results appear to be consistent and suggest that methylation causes CpG site depletion in the CDSs (which translates to smaller CpG O/E values for more frequently methylated genes).

**Figure 10:**
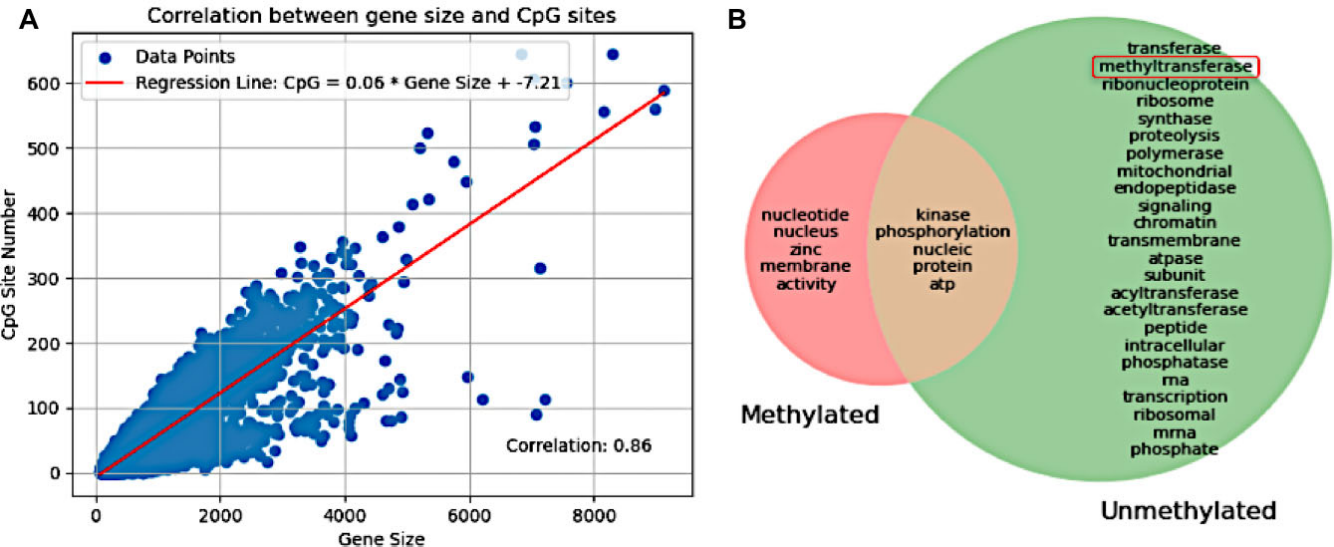
Possible functions of methylated and unmethylated CDSs. (A) Linear relationship between gene/CDS size and number of available CpG sites as shown by linear regression analysis and measured using Pearson correlation coefficient *r* = 0.86. (B) KeyBERT keyword extraction set intersection between unmethylated and methylated genome portions of all complete Myxozoa genome assemblies. The relationship between these 2 portions is displayed as a Venn diagram, in which the unmethylated portion is the bigger part, with a small number of shared keywords related to protein handling being the only similarity to the methylated portion.

Pearson correlation analysis indicated a moderate negative linear relationship between the CpG O/E values for the CDSs and the recorded ONT Nanopore methylation frequencies (*r* = −0.5028 and *p* = 0.0). Our analyses collectively indicate that CDS regions in Myxozoa are less variable and markedly more GC-rich compared to non-CDS regions. The fact that CDS regions of all the analyzed genomes also have a higher degree of CpG regions compared to non-CDS regions provides strong support that myxozoan CDS regions are more likely to be sites for CpG methylation. The CpG content in the context of myxozoan gene length was also analyzed and a linear relationship was found, further supporting this (Fig. 10A). The CDS group with CpG O/E below the regression line represented the genes that are more likely to be methylated, while the group with CpG O/E above this line represented the genes most likely to be unmethylated (Fig. 10A). This was performed on the myxozoan genome assemblies with MetaEuk predicted CDS sequences. The partial genome assembly of *Ceratomyxa* sp. sample 58 is provided to demonstrate the observation of GC content depletion in methylated CpG across coding sequences (Supplementary File 10B). This partial assembly benefits from the specimen having been sequenced in a highly methylated state, highlighting comparison between methylated and unmethylated CDS regions and the corresponding GC content.

When myxozoan gene annotations were grouped based on corresponding CDS CpG O/E values (using the weighted KDE CpG O/E peaks as thresholds [Fig. 8B] for selecting methylated/unmethylated CDSs), ontologies could be linked to the annotations within each group. Using keyword extraction methods, 200 keywords were selected that were able to capture and represent the GO inferred terms related to functions, processes, and cellular compartments that best represent each group of CDSs (Fig. 10B). When the intersecting terms common to all myxozoan genomes within each group were analyzed, it was discovered that genes at the low end of CpG O/E (the ones with high probability of being methylated—i.e., transcriptionally silenced) form a smaller intersection characterized by terms linked to activity (Fig. 10B). On the opposite end, the high CpG O/E genes (most likely not being methylated—i.e., transcriptionally active) formed a larger intersection linked to transcription, translation, and other activities that fit a description of housekeeping genes, notably including genes encoding methylation mechanisms.

## Discussion

### New high-quality myxozoan genome assemblies

Herein, we present the first comprehensive analysis of myxozoan DNA using ONT. This long-read sequencing technology, coupled with downstream bioinformatic processing, generated assemblies of 5 myxozoan genomes, all free from detectable host DNA contamination. The sequences of the 5 species did not match any available myxozoan sequences deposited in the SILVA Release 138.1 nonredundant Small Subunit rRNA Database [56]. We have therefore designated the 5 myxozoan assemblies deposited in the NCBI database as *Ceratomyxa* sp. sample 57-BR2022, *Ceratomyxa* sp. sample 58-BR2022, *Ellipsomyxa* sp. sample 70-BR2022, Myxosporea sp. sample 108-BR2022, and *Ellipsomyxa* sp. sample 115-BR2022.

QUAST and BUSCO metrics confirmed the exceptional quality of these 5 new assemblies, surpassing the assembly statistics of the 8 existing myxozoan genomes available in public databases. Functional annotation of all 5 assembled genomes and reannotation of the 8 existing myxozoan genomes using the same pipeline provided uniformly annotated datasets for comparison. Annotations derived from BUSCO, PANTHER HMM, and GO analyses revealed highly heterogeneous protein annotations. Likely causes of this heterogeneity include short, highly fragmented reads and significant amounts of contaminant DNA in extant myxozoan assemblies. Our results suggest that such extant myxozoan genome assemblies should be resequenced using long-read technologies. To this end, it is worth noting that in our experience, obtaining consistent high-quality long sequence reads depends critically on the collection, storage, and transport of myxozoan infected material and on DNA handling time. Future experimentation will focus on an in-field DNA extraction procedure akin to that achieved for microbial symbionts of marine invertebrates [82]. We therefore plan an in-field extraction procedure optimized beyond a crude template followed immediately by genome sequencing using Oxford Nanopore PromethION technology. This in-field approach has been used to characterize specific genes for other parasites (e.g., malaria parasites [83] and *Blastocystis* spp. [84]) and should also enable generating genome data. It is important to highlight that host DNA contamination is almost inevitable in all genome sequencing attempts on myxozoans due to their endoparasitic lifestyles. This can be at least partially addressed/minimized by mitigating the ratio of host to parasite reads. We found that failure to address challenges relating to read quality and contamination may lead to false inferences of myxozoan genome sizes and phylogenetic relationships. Our approach to deal with this issue was to use minimap2 in order to map all reads onto a large set of reference fish genomes and then assemble the nonmapping reads. These assembled contigs were then screened by BLAST comparison against comprehensive databases such as NT and tsa_NR in order to remove even the slightest remaining homologies to fish hosts.

### Robust methylation detection

The experimental approaches we took to both determine and validate cytosine methylation at CpG sites across all available myxozoan genome assemblies counter the assertion that myxosporeans are among the few animals that have secondarily lost cytosine methylation capability [10]. Our analyses indicated that methylation is indeed present in most myxozoan genomes based on experimentally obtained ONT Nanopore methylation calling and by *in silico* modeling component means distance criteria. The model-obtained observation was further validated and confirmed by a nonparametric method, kernel density estimation. The relationship between methylation and genome defence in Cnidaria [85] would predict that methylated genes are significantly longer than unmethylated genes. We therefore analyzed CpG content in the context of myxozoan gene length using *Ellipsomyxa* sp. sample 70 as the best available myxozoan assembly with limited to no contamination. Accordingly, we found a linear relationship between gene length and number of methylation targets (i.e., the longer the gene, the more methylation targets [CpG sites] it possessed) as expected for cnidarians. The relationship was so straightforward that it could be used to predict the number of CpG sites purely based on gene length.

### GC content and DNA methylation

It is known that GC-rich regions provide more targets for methylation and show a greater frequency of methylated sites than GC-poor regions [86]. Usually, a telltale mark of genome methylation is loss of CpG dinucleotide sites, referred to as CpG depletion (CpG O/E) [87], which results due to increased mutation in methylated CpGs. The value is based on the observed frequency of CpG dinucleotides relative to the product of the frequency of the individual nucleotides (G and C) weighted by the length of a genomic region [87]. This ratio is notably low in vertebrate genomes (e.g., <0.20 in the human genome [88]). Invertebrate genomes, on the other hand, are known to be much less methylated overall. Their methylation pattern is often described as “mosaic-like” [5, 89]—with some CDSs experiencing frequent methylation and others none at all. Since the general invertebrate methylation pattern includes gene bodies instead of non-CDS promoter sites, we divided the myxozoan genome assemblies accordingly and confirmed that Myxozoa are no exception to this. For all the myxozoan genomes analyzed, the CDS sequences are characterized by increased GC content accompanied by more CpG sites when compared to non-CDS regions. Research on 2 sponges, a cnidarian and a ctenophore, provides evidence that DNA methylation was present in early metazoans and has largely been conserved [90]. Although the CpG methylation system has apparently been lost in some invertebrates, most notably *C. elegans* [91], for others, like *Drosophila*, this has been disputed after the initial claims [92]. We suggest that invertebrates currently believed to possess no methylation may nevertheless employ this epigenetic process, albeit not in the expected way.

The most plausible explanation for the observed CpG O/E drop in bimodally distributed CDS clusters that we identified in myxozoan genomes appears to relate to the DNA methylation process. Determining the overall genomic CDS CpG O/E ratio is a very significant indicator of DNA methylation for the following reasons. CDSs that are often methylated will inevitably display markedly lower CpG O/E values than sequences that are not methylated. This is due to an increased rate of mutation in methylated CpG that leads to GC content loss, with C-to-T transitions being the most common type of mutation at these sites. This GC loss will lead to a characteristic 2-peak bimodal distribution of CpG/OE values, which has been observed in all invertebrate species known to methylate DNA and herein (Fig. 8). Additionally, when the distributions of the ratio between CDS and non-CDS regions of the genomes were compared, most suspected methylation events occurred in CDS regions as expected, since this is a tell-tale mark of invertebrate methylation (unlike in vertebrates, which skip non-CDS regions). The GC content has significant biological consequences reflected in gene inactivation and genome instability that could explain reported variation in Myxozoa genome sizes. Except for the partial genomes of *Ceratomyxa* sp. sample 57 and *Ceratomyxa* sp. sample 58, myxozoan genomes are characterized by a GC content of less than 40%. Although the overall genome GC content is highly variable, the CDS sequences are less variable and markedly more GC-rich than sequences in non-CDS regions. This CDS and non-CDS GC content dichotomy within myxozoan genomes is likely a result of a complex interplay between functional requirements, mutational biases, repair mechanisms, recombination patterns, and evolutionary forces. Evidence that myxozoan CDS sequences are actively methylated in myxozoans includes their greater GC content, a higher frequency of CpG regions in CDS than in non-CDS sequences in all analyzed genomes, and 2 distinct subpopulations with markedly different CpG O/E values. Another study has observed that methylated invertebrate genes generally encode for housekeeping functions related to transcription and translation, while nonmethylated genes generally encode for functions including cellular signaling and reproductive processes [93]. This observation was also indicated in myxozoans based on our GO analyses, which leads to new questions regarding the enzymatic machinery behind the methylation process acting upon these distinct groups of CDSs.

### DNA methylation mechanisms and patterns

Our data have enabled us to demonstrate that there is extensive methylation in some genomes (*Ceratomyxa* sp. samples 57 and 58) and low levels of methylation in others (Myxosporea sp. sample 108, *Ellipsomyxa* sp. sample 115, and *Ellipsomyxa* sp. sample 70). Our epigenetic data and the CpG O/E values calculated from the genome assemblies in the form of a weighted KDE enabled us to more precisely define CpG O/E boundaries and more accurately pinpoint which CDSs were actively methylated. We believe this is the first study to use epigenetic methylation frequency results in combination with genomic derived CpG O/E values to assess the frequency of methylation at specific genetic loci. This approach has potential as a preliminary screening method to identify genes of interest for targeted studies to resolve epigenetic processes, including regulatory mechanisms. To further examine DNA methylation mechanisms, we searched for the presence of DNMT-, TET-, and BER-related enzymes in the annotated genomes. The presence of such enzymes would be a strong function-related indicator for the potential to methylate/demethylate DNA. Both PANTHER HMM-based searches and MetaEuk protein annotation indicated that all 3 classes of methylation-relevant enzymes were present. Our results demonstrate that BER is the most abundant methylation component, followed by DNMT and finally TET. Functional annotation has thus revealed the presence of key DNA methylation enzymes (DNMTs, TETs, BERs) in all analyzed myxozoan genomes. However, traces of contamination in some previously sequenced myxozoan genomes were detected. This again highlights the importance of rigorous scrutiny and filtering when performing sequencing and assembly of myxozoans.

Overall, our ONT-based experimental data revealed a striking feature—either the overwhelming presence or near-complete absence of methylation across 5 Myxozoa genomes, with samples *Ceratomyxa* sp. sample 57 and *Ceratomyxa* sp. sample 58 exhibiting extremely high levels of methylation, and Myxosporea sp. sample 108, *Ellipsomyxa* sp. sample 115, and *Ellipsomyxa* sp. sample 70 only marginal ones (Fig. 7). However, all these genomes were strongly predicted to have distinct populations of CDSs being methylated by our *in silico* analyses. To ensure that the low methylation signals were not false positives, the methylation pattern across the entire genome sequence of our most complete assembly (*Ellipsomyxa* sp. sample 70) was compared to the methylation patterns in sequences previously discharged during the removal of host contamination but annotated as fish sequences. This comparison confirmed that the methylation patterns were completely different (Supplementary File 10C). This observation might also explain the conclusions reached by Kyger et al. [10]. If the DNA templates sequenced in that study were obtained from specimens at a point in a parasite life stage when most genes were upregulated, then little if any methylation would be detected. This might also explain our results for *Ceratomyxa* sp. sample 57 and *Ceratomyxa* sp. sample 58. We note that high numbers of mature spores were particularly evident microscopically for *Ceratomyxa* sp. sample 57 and *Ceratomyxa* sp. sample 58 (Adriano, personal observation). Perhaps extensive downregulation characterizes this period of development. Future experimentation is warranted to examine whether there may be some myxozoan life history stages when nearly universal genome methylation is required (“switched on”). Examining patterns of genome methylation will provide valuable new insights into the epigenetic regulation of myxozoan development and host–parasite interactions.

## Conclusions

Our comprehensive analyses of contiguous long sequence reads provide the first evidence for gene body methylation in Myxozoa. We found that DNA methylation was generally either overwhelmingly present or almost completely absent in the material analyzed. *In silico* analyses, based on GC content modeling and empirically derived methylation frequencies, consistently identified methylation as a factor in shaping genomic landscapes of myxozoans. Most analyzed genomes display clear signs of methylation taking place within gene bodies, based on evidence in the form of distinctly lower CpG O/E gene groups. Further examination of the associated genomic architecture, mechanisms, and timing of epigenetic control of gene transcription in myxozoans will expand our understanding of development and parasite–host–environment interactions. It may also enable novel control strategies for these important fish pathogens.

## Availability of Source Code and Requirements

Project name: Myxozoa_supplementary

Project homepage: https://github.com/astarsky2016/Myxozoa_supplementary [94]

Operating system(s): Platform independent

Programming language: Jupyter Notebook and Python

Other requirements: No

License: MIT license

## Supplementary Material

giaf014_Supplemental_Files

giaf014_GIGA-D-24-00150_Original_Submission

giaf014_GIGA-D-24-00150_Revision_1

giaf014_GIGA-D-24-00150_Revision_2

giaf014_GIGA-D-24-00150_Revision_3

giaf014_Response_to_Reviewer_Comments_Original_Submission

giaf014_Response_to_Reviewer_Comments_Revision_1

giaf014_Response_to_Reviewer_Comments_Revision_2

giaf014_Reviewer_1_Report_Original_Submission Jiayong Zhong -- 7/16/2024

giaf014_Reviewer_1_Report_Revision_1 Jiayong Zhong -- 10/17/2024

giaf014_Reviewer_1_Report_Revision_2 Jiayong Zhong -- 11/25/2024

giaf014_Reviewer_2_Report_Original_Submission Chentao Yang -- 7/25/2024

giaf014_Reviewer_2_Report_Revision_1 Chentao Yang -- 10/22/2024

giaf014_Reviewer_2_Report_Revision_2 Chentao Yang -- 12/1/2024

giaf014_Reviewer_2_Report_Revision_3 Chentao Yang -- 12/18/2024

## Data Availability

The 5 myxozoan assemblies, clean from any detectable contamination, are deposited in the NCBI database as follows: All relevant datasets, including the raw data and more detailed outputs of genome assembly metrics comparison, protein annotations, and Gene Ontology Enrichment analysis, have been uploaded in the *GigaScience* repository, GigaDB [95–98], and are publicly available. The accompanying readme.txt in GigaDB lists all uploaded files with a short description of the content of each file, as well as a note to any manuscript figure that the data relate to.

## Additional Files

**Supplementary File 1:** Reference fish genomes.xlsx.

**Supplementary File 2:** Available myxozoa genomes.txt.

**Supplementary File 3:** 18S rDNA sequences.txt.

**Supplementary File 4:** Photos of parasites and fish hosts.pptx.

**Supplementary File 5:** Myxozoa_SSU_annotated.docx.

**Supplementary File 6A:** Myxozoa phylogenetic tree.eps.

**Supplementary File 6B:** Newick representation phylogeny.txt.

**Supplementary File 7:** Assembly statistics.pdf.

**Supplementary File 8:** Genome coverage plot Sample 70.docx.

**Supplementary File 9:** Word cloud analysis.docx.

**Supplementary File 10:** GC content of CDS regions.docx.

## Abbreviations

AIC: Akaike information criterion; BER: base-excision repair; BLAST: Basic Local Alignment Search Tool; BUSCO: Benchmarking Universal Single-Copy Orthologs; CpG islands: DNA sequences rich in guanine and cytosine and contain clusters of CpG dinucleotides; CpG O/E: observed–expected CpG ratio; DNMTs: DNA methyltransferases; EST: expressed sequence tag; FASTA: text-based format representing either nucleotide or amino acid sequences; FASTA5: format from Oxford Nanopore (ONT) for storing and managing data that retains epigenetic information; Gb: gigabase pairs; GMM: Gaussian mixture model; GO: Gene Ontology; HMM: hidden Markov model, a statistical model that describes evolution of observable events dependent on internal factors, not directly observable; KDE: kernel density estimation; 5-Mc: 5-methylcytosine; Mb: megabases pairs; MBDs: methyl-CpG binding domain proteins; N50, N90, L50, and L90: metrics to evaluate quality of genome assembly, specifically indicating how contiguous the assembled sequences are, with “N” representing a sequence length and “L” representing the number of sequences needed to reach a certain percentage of the genome size; NCBI: National Center for Biotechnology Information; NGS: next-generation sequencing; ONT: Oxford-Nanopore PromethION sequencing; Savitzky–Golay filter: digital filter for smoothing data; SILVA: a comprehensive online resource for quality checked and aligned ribosomal RNA sequence data; TET: DNA demethylation; UniProt: high-quality, comprehensive protein resource with expert review and annotation of proteins; WGBS: whole-genome bisulfate sequencing.

## Benefit Sharing Statement

A research collaboration was developed with scientists from the countries providing genetic samples. All collaborators are included as coauthors, and the results of research have been shared with the provider communities and the broader scientific community. The research addresses a priority concern, in this case the conservation of organisms being studied. More broadly, our group is committed to international scientific partnerships, as well as institutional capacity building.
