# Supplementary material for: Long-read metagenomic sequencing negates inferred loss of cytosine methylation in Myxosporea (Cnidaria: Myxozoa)
Source: Gigascience. 2025 Mar 13;14:giaf014. doi: 10.1093/gigascience/giaf014 (PMC11905887; doi:10.1093/gigascience/giaf014)
Supplement: giaf014_Supplemental_Files [file giaf014_supplemental_files.zip › Supplementary FIle 10_GC content of CDS regions.docx]

**(A)**


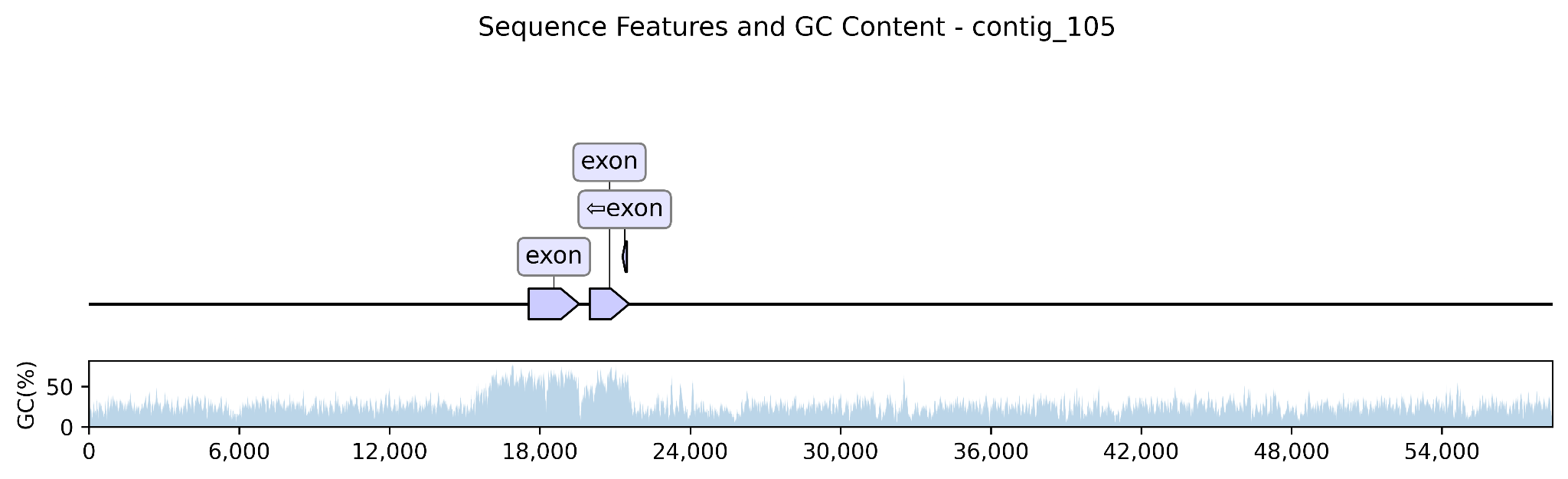

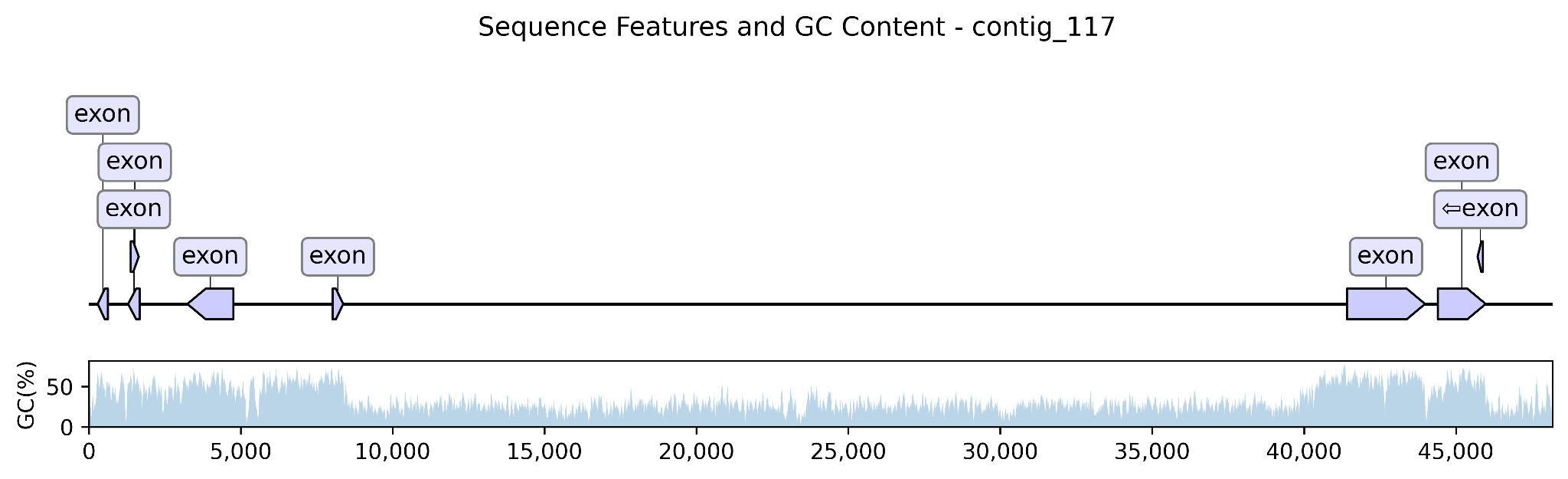


**(B)**


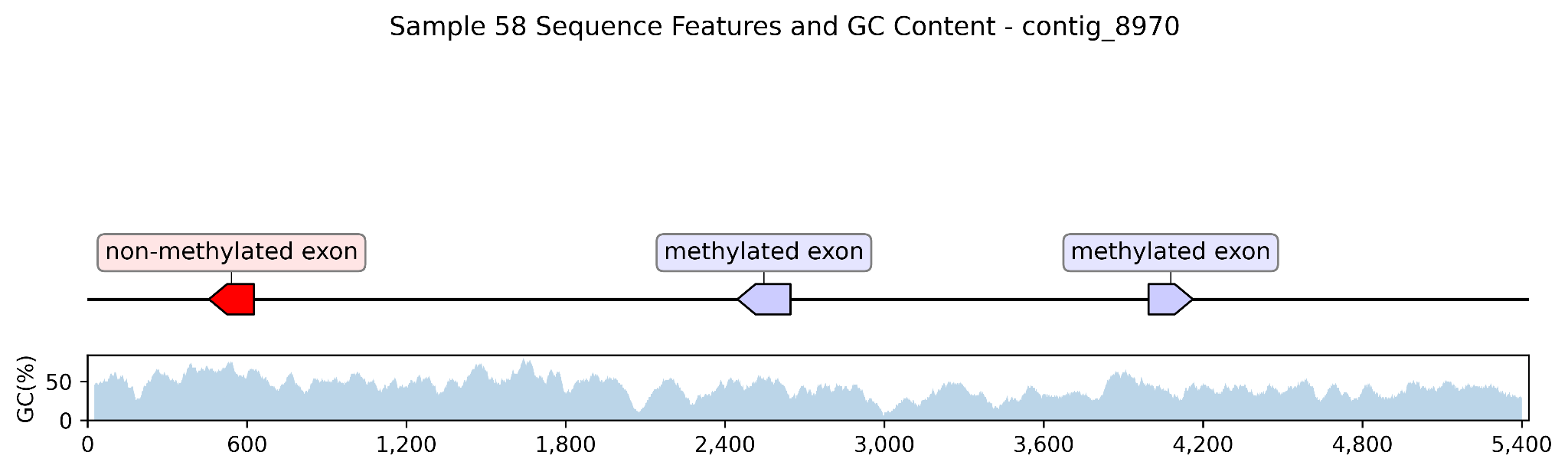


**(C)**

**
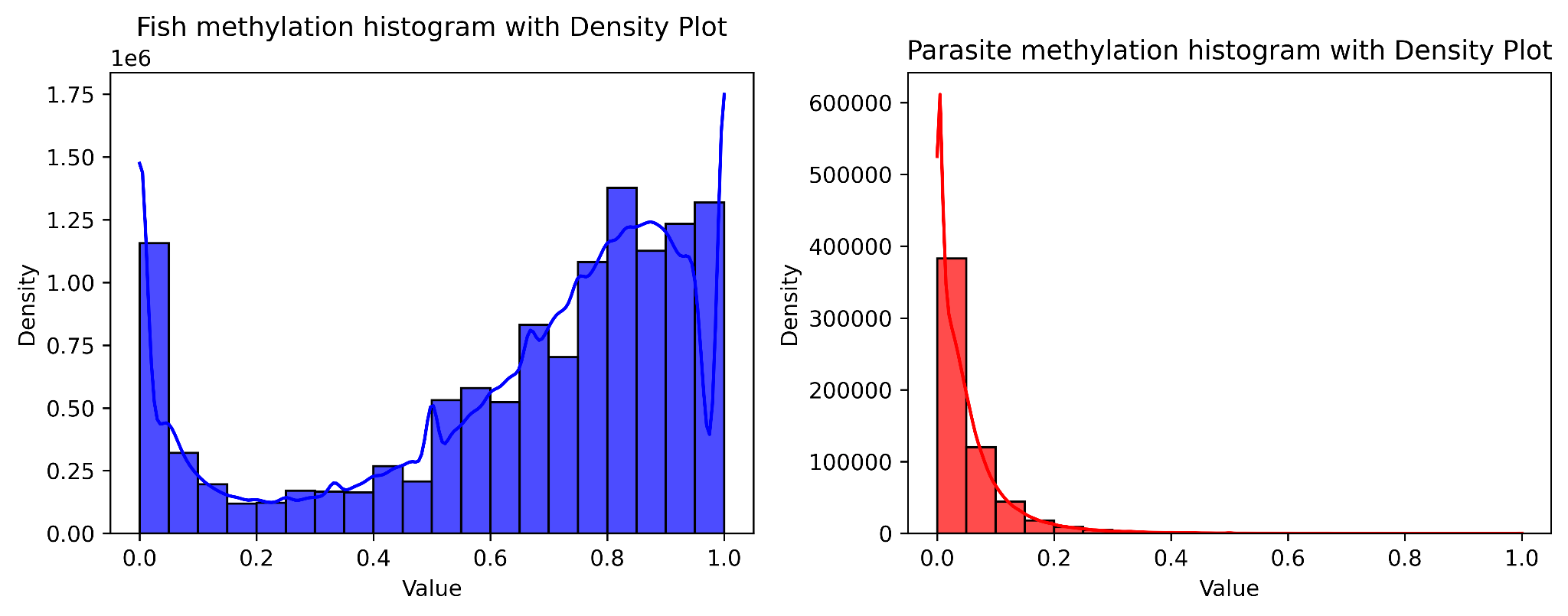
**

Supplementary Figure 10: (A) Sample 70 contig_105 and contig_117 coding sequence (exon) and GC content plots displaying higher GC content of CDS regions compared to non-coding parts of the genome. Plots have been generated using the DNA Features Viewer package (<https://edinburgh-genome-foundry.github.io/DnaFeaturesViewer/>). (B) Slightly higher GC content of sample 58 non-methylated CDS region compared to methylated ones. (C) Different methylation frequency distribution in vertebrate fish host compared to invertebrate myxozoan parasite one.
