# Supplementary material for: Long-read metagenomic sequencing negates inferred loss of cytosine methylation in Myxosporea (Cnidaria: Myxozoa)
Source: Gigascience. 2025 Mar 13;14:giaf014. doi: 10.1093/gigascience/giaf014 (PMC11905887; doi:10.1093/gigascience/giaf014)
Supplement: giaf014_Supplemental_Files [file giaf014_supplemental_files.zip › Supplementary File 4_Photos of parasites and fish hosts.pptx]

## Slide 1
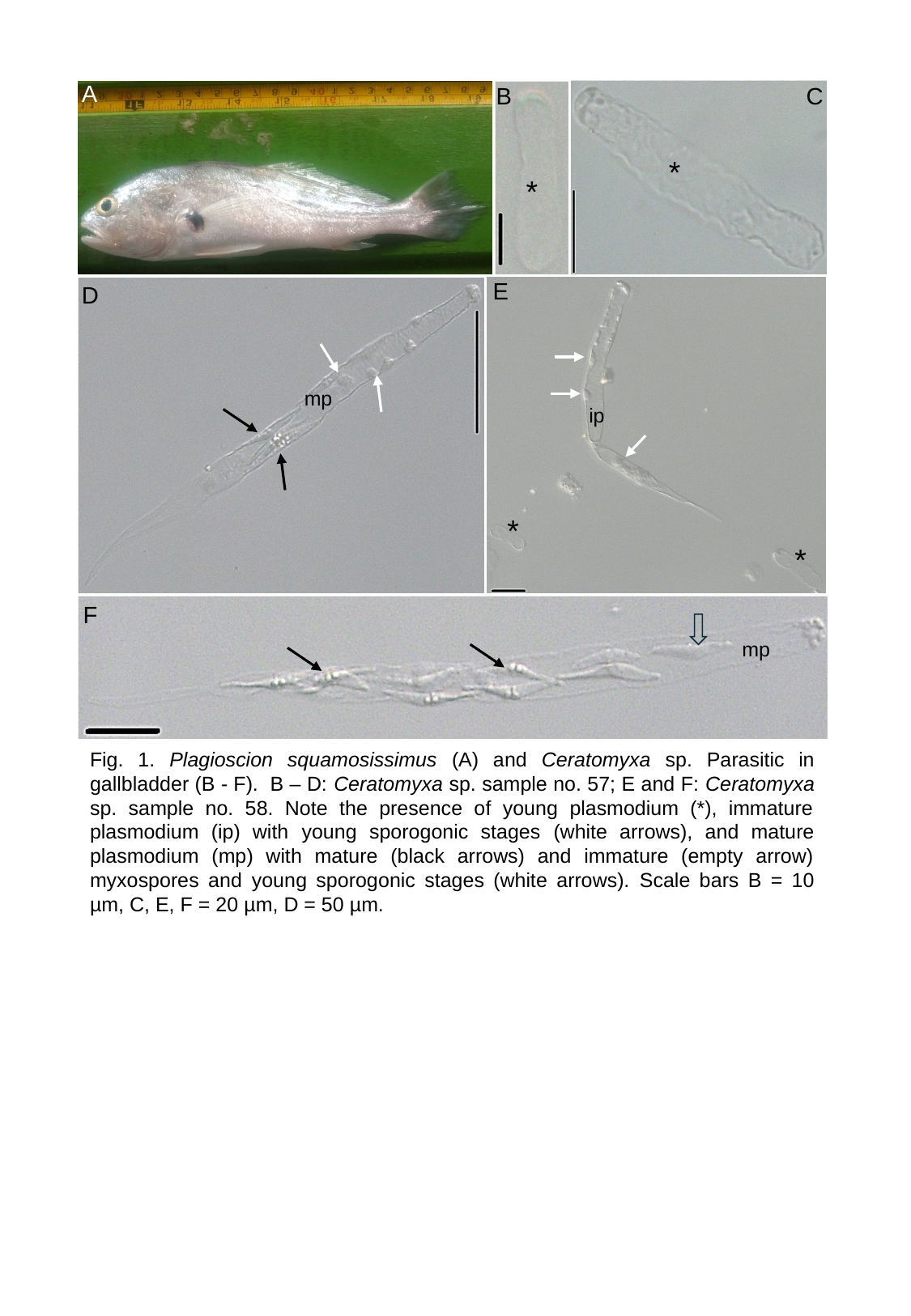

A
B
C
*
*
E
D
mp
ip
*
*
F
mp
Fig. 1. Plagioscion squamosissimus (A) and Ceratomyxa sp. Parasitic in gallbladder (B - F). B – D: Ceratomyxa sp. sample no. 57; E and F: Ceratomyxa sp. sample no. 58. Note the presence of young plasmodium (*), immature plasmodium (ip) with young sporogonic stages (white arrows), and mature plasmodium (mp) with mature (black arrows) and immature (empty arrow) myxospores and young sporogonic stages (white arrows). Scale bars B = 10 µm, C, E, F = 20 µm, D = 50 µm.

## Slide 2
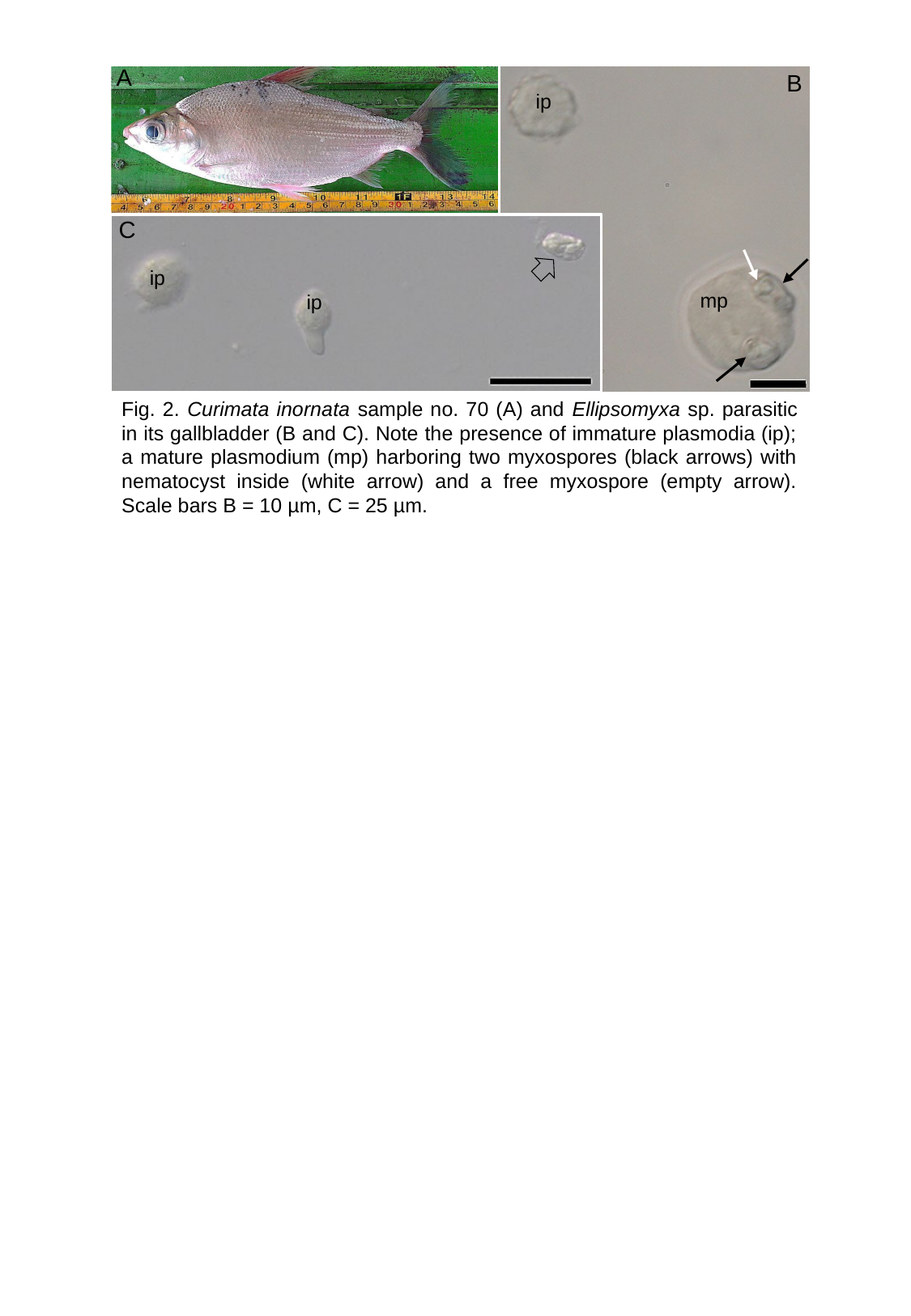

A
B
ip
C
ip
mp
ip
Fig. 2. Curimata inornata sample no. 70 (A) and Ellipsomyxa sp. parasitic in its gallbladder (B and C). Note the presence of immature plasmodia (ip); a mature plasmodium (mp) harboring two myxospores (black arrows) with nematocyst inside (white arrow) and a free myxospore (empty arrow). Scale bars B = 10 µm, C = 25 µm.

## Slide 3
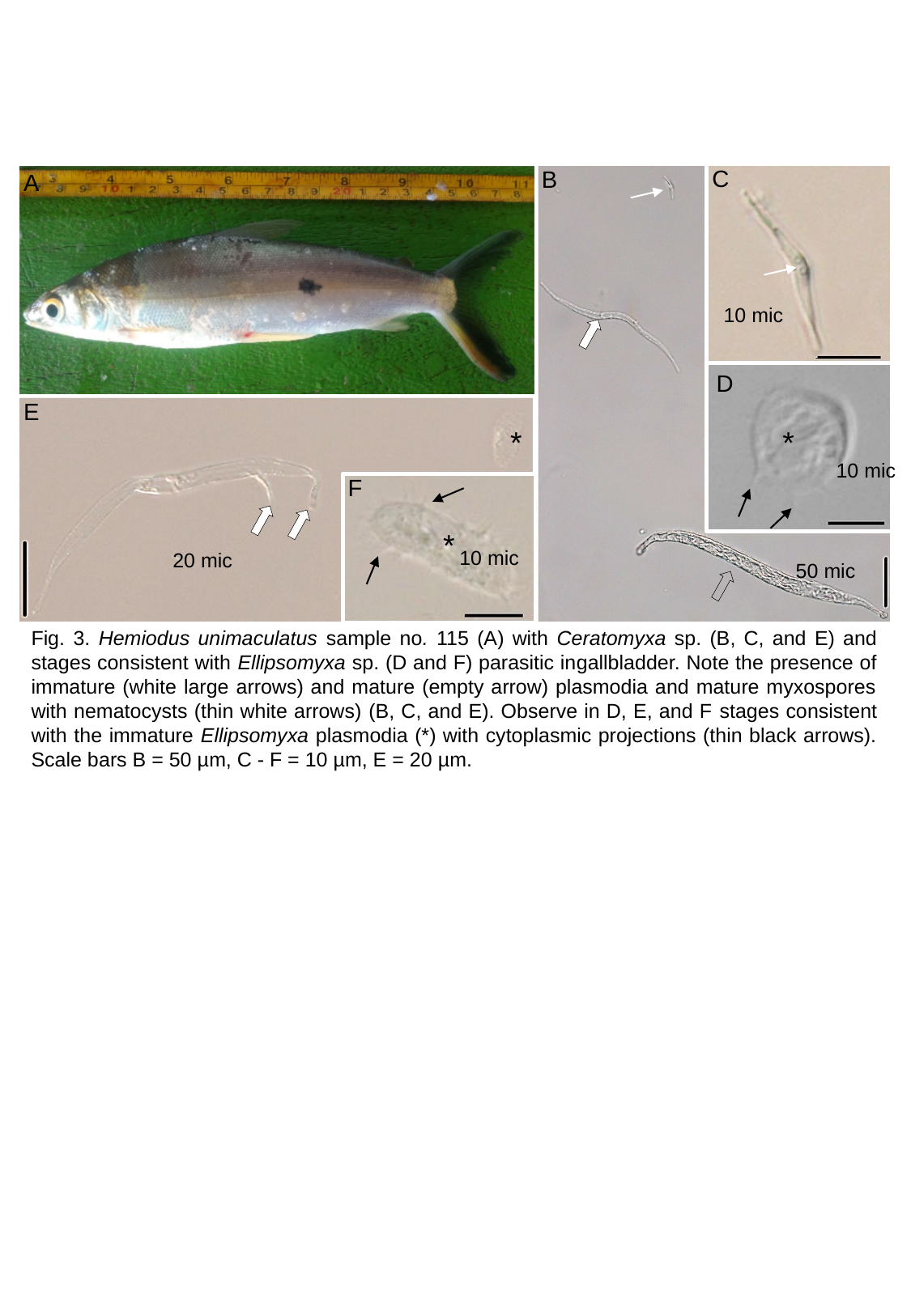

C
B
A
10 mic
D
E
*
*
10 mic
F
*
10 mic
20 mic
50 mic
Fig. 3. Hemiodus unimaculatus sample no. 115 (A) with Ceratomyxa sp. (B, C, and E) and stages consistent with Ellipsomyxa sp. (D and F) parasitic ingallbladder. Note the presence of immature (white large arrows) and mature (empty arrow) plasmodia and mature myxospores with nematocysts (thin white arrows) (B, C, and E). Observe in D, E, and F stages consistent with the immature Ellipsomyxa plasmodia (*) with cytoplasmic projections (thin black arrows). Scale bars B = 50 µm, C - F = 10 µm, E = 20 µm.

## Slide 4
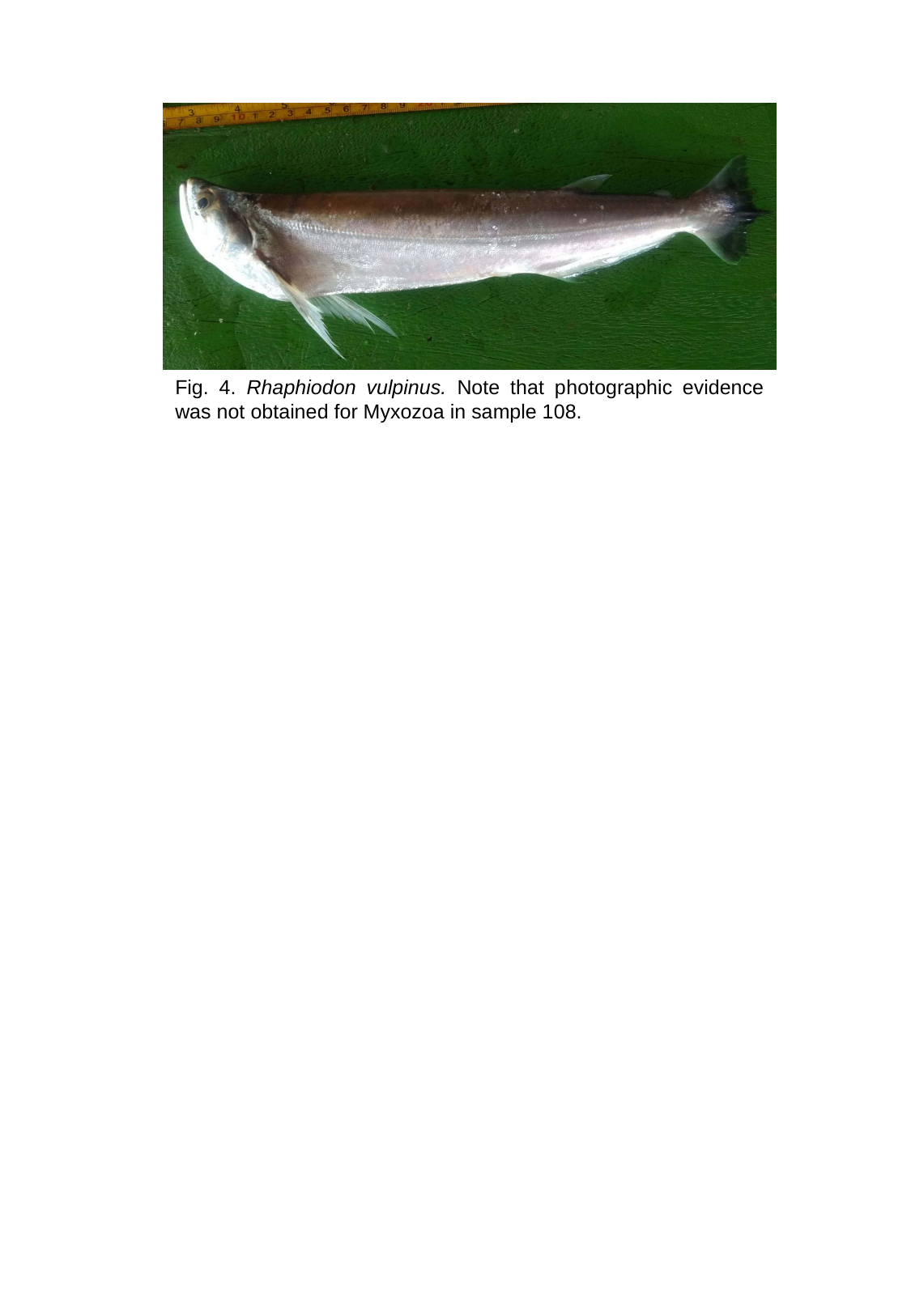

Fig. 4. Rhaphiodon vulpinus. Note that photographic evidence was not obtained for Myxozoa in sample 108.
