# Supplementary material for: Long-read metagenomic sequencing negates inferred loss of cytosine methylation in Myxosporea (Cnidaria: Myxozoa)
Source: Gigascience. 2025 Mar 13;14:giaf014. doi: 10.1093/gigascience/giaf014 (PMC11905887; doi:10.1093/gigascience/giaf014)

Supplementary File 5: **18S rRNA secondary structure diagram based on 678 aligned Myxozoa SSUs.** The red positions are highly variable and were identified using a masking algorithm that is included in SSU-align software. These positions correlate with SSU variable regions V1-V9 and have been marked as such. One noticeable difference is the absence of the V6 hypervariable region, that exists as a structure, but appears to be conserved in Myxozoa.


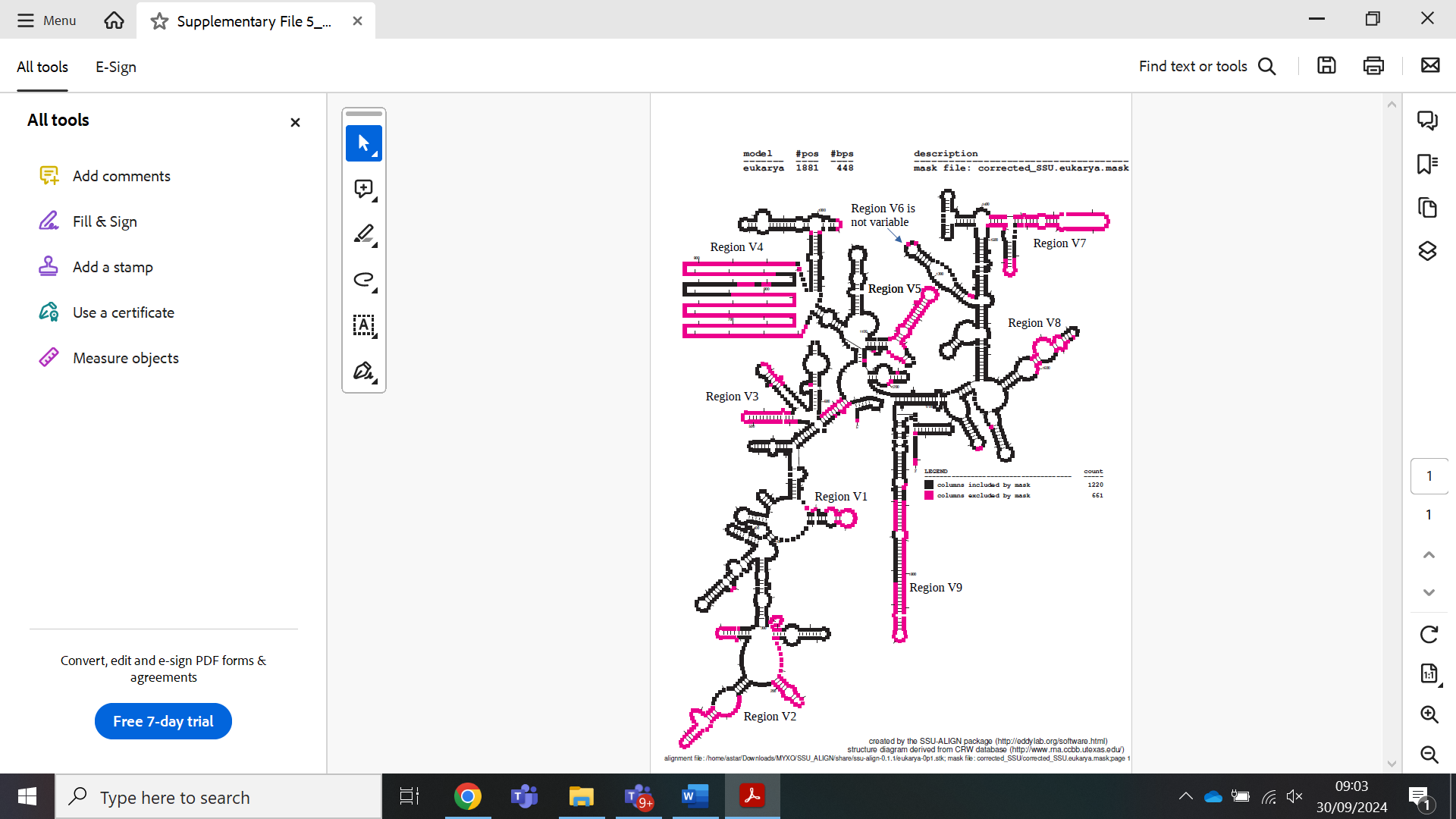

Supplement: giaf014_Supplemental_Files [file giaf014_supplemental_files.zip › Supplementary File 5_Myxozoa_SSU_annotated.docx]
