# Supplementary material for: Long-read metagenomic sequencing negates inferred loss of cytosine methylation in Myxosporea (Cnidaria: Myxozoa)
Source: Gigascience. 2025 Mar 13;14:giaf014. doi: 10.1093/gigascience/giaf014 (PMC11905887; doi:10.1093/gigascience/giaf014)
Supplement: giaf014_Supplemental_Files [file giaf014_supplemental_files.zip › Supplementary File 7_Assembly statistics.pdf]

a.) Table "Report" summarizes QUAST metrics for all assemblies being compared (in separate columns). This is the metrics description: # contigs (≥ x bp) is total number of contigs of length ≥ x bp. Total length (≥ x bp) is the total number of bases in contigs of length ≥ x bp. # contigs is the total number of contigs in the assembly. Largest contig is the length of the longest contig in the assembly. Total length is the total number of bases in the assembly. GC (%) is the total number of G and C nucleotides in the assembly, divided by the total length of the assembly. N50 is the length for which the collection of all contigs of that length or longer covers at least half an assembly. Nx (for x between 0 and 100) is defined similarly to N50 but with x % instead of 50 %. L50 (Lx) is the number of contigs equal to or longer than N50 (Nx). In other words, L50, for example, is the minimal number of contigs that cover half the assembly.

b.) Nx plot shows Nx values as x varies from 0 to 100 % for all compared genomes (marked by different colours in the legend).

c.) Cumulative length plot shows the growth of contig lengths. On the x-axis, contigs are ordered from the largest to smallest. The y-axis gives the size of the x largest contigs in the assembly for all the genomes being compared.

d.) GC content plot by # windows shows the distribution of GC content in the contigs. The x value is the GC percentage (0 to 100 %). The y value is the number of non-overlapping 100 bp windows which GC content equals x %. For a single genome, the distribution is typically Gaussian. However, for assemblies with contaminants, the GC distribution appears to be a superposition of Gaussian distributions, giving a plot with multiple peaks.

e1 - e11.) GC content plot by # contigs shows the distribution of # contigs with GC percentage in a certain range for each genome on a separate plot. The x value is the GC percentage intervals (width is 5 %). The y value is the number of contigs which GC content lies in the corresponding interval.

a.) Report

	sample_108_ellipsomyxa_sp	sample_115_ellipsomyxa_sp	sample_70_ceratomyxa_sp	Ceratonova_shasta	Enteromyxium_leei	Henneguya_salminicola	Kudoa_iwatai	Myxobolus_honghuensis	Myxobolus_squamalis	Sphaeromyxa_zaharoni	Thelohanellus_kitauei
# contigs (>= 0 bp)	377	267	73	14586	69053	18330	1639	169493	37921	70914	5757
# contigs (>= 1000 bp)	367	245	72	3939	18239	11246	1407	60960	12854	42763	5757
# contigs (>= 5000 bp)	351	210	68	2299	509	3530	1036	9636	521	8671	1921
# contigs (>= 10000 bp)	320	140	60	1663	59	1429	851	2808	81	2634	1810
# contigs (>= 25000 bp)	251	88	34	859	0	159	463	380	9	113	1048
# contigs (>= 50000 bp)	187	54	24	329	0	9	151	59	3	1	741
Total length (>= 0 bp)	26974870	30225464	24261813	69794587	68163509	61443780	31197353	254310768	43671844	173585031	150348159
Total length (>= 1000 bp)	26967544	30208071	24261238	66310516	34028660	56536953	31031552	206775870	26629287	153586368	150348159
Total length (>= 5000 bp)	26924652	30110813	24248663	62418945	3707275	38854413	30143113	95661821	4206405	80332694	140112525
Total length (>= 10000 bp)	26685393	29596112	24185424	57823607	813359	23922618	28804415	49246717	1367076	38496209	139329663
Total length (>= 25000 bp)	25635810	28846008	23755363	44616649	0	5391334	22184828	14447662	381364	3427985	127976971
Total length (>= 50000 bp)	23212011	27763465	23417212	25978527	0	540079	10916165	3901386	183184	50567	116822939
# contigs	376	267	73	5506	69053	18330	1639	100771	37919	70914	5757
Largest contig	435480	1725202	3566344	452519	23997	106326	139679	130643	67459	50567	1160795
Total length	26974383	30225464	24261813	67382268	68163509	61443780	31197353	234480756	43671040	173585031	150348159
GC (%)	29.95	29.95	39.39	24.11	33.51	28.96	23.64	22.24	27.29	28.02	31.23
N50	128514	757682	1371065	37829	998	7570	40195	3799	1287	4474	149756
N90	41541	92839	392245	6949	560	1189	12003	927	585	926	12732
auN	145856.3	768309.8	1719141.7	50426.8	1736.1	10574.5	44968.1	7698.8	2592.7	6549.6	210183.5
L50	70	14	6	507	18293	2212	258	14605	8607	10039	268
L90	211	47	17	2001	56147	10115	785	65384	29832	45508	1455
# N's per 100 kbp	0.00	0.00	0.00	727.38	6.82	0.07	1.10	28050.55	0.00	0.33	18364.41

All statistics are based on contigs of size >= 500 bp, unless otherwise noted
(e.g., "# contigs (>= 0 bp)" and "Total length (>= 0 bp)" include all contigs).

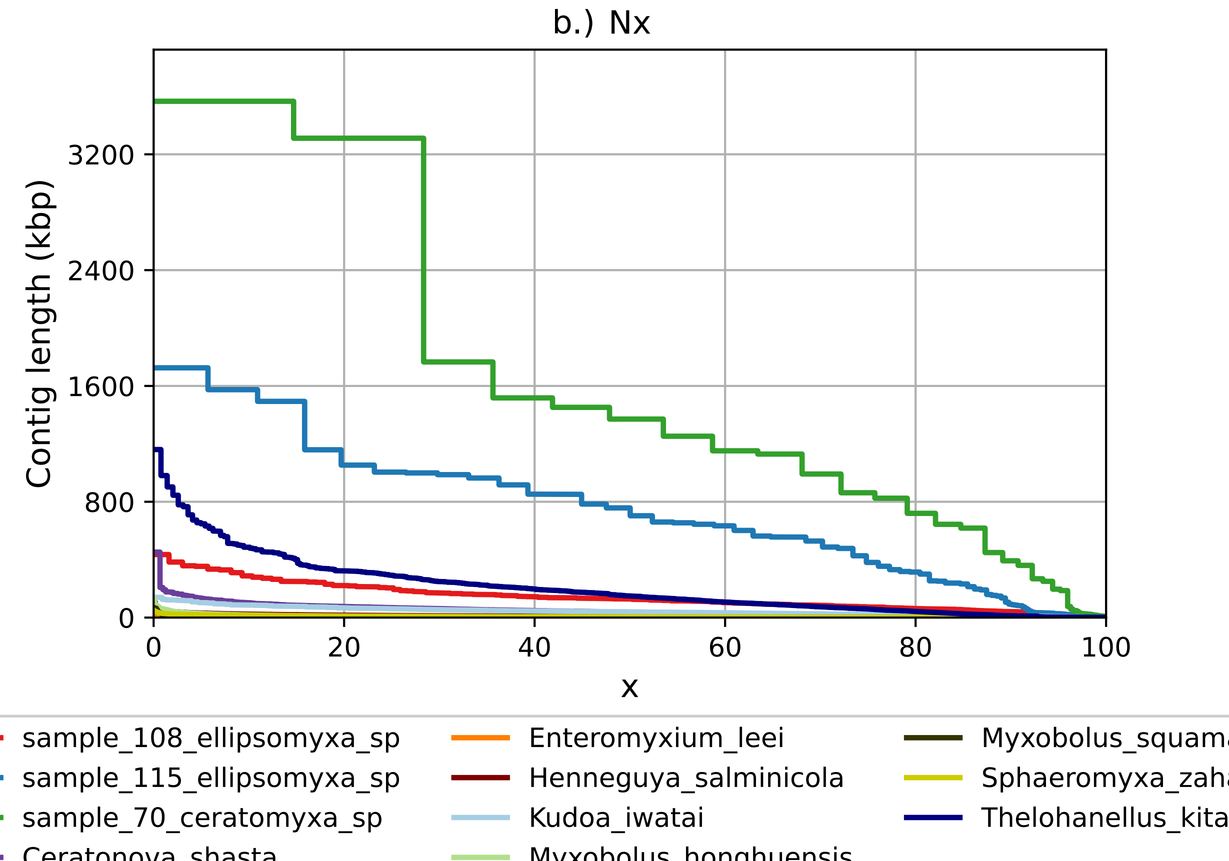

b.) Nx
Contig length (kbp)
3200
2400
1600
800
0
0
20
40
60
80
100
x
sample_108_ellipsomyxa_sp
sample_115_ellipsomyxa_sp
sample_70_ceratomyxa_sp
Ceratonova_shasta
Enteromyxium_leei
Henneguya_salminicola
Kudoa_iwatai
Myxobolus_honghuensis
Myxobolus_squam
Sphaeromyxa_zah
Thelohanellus_kita

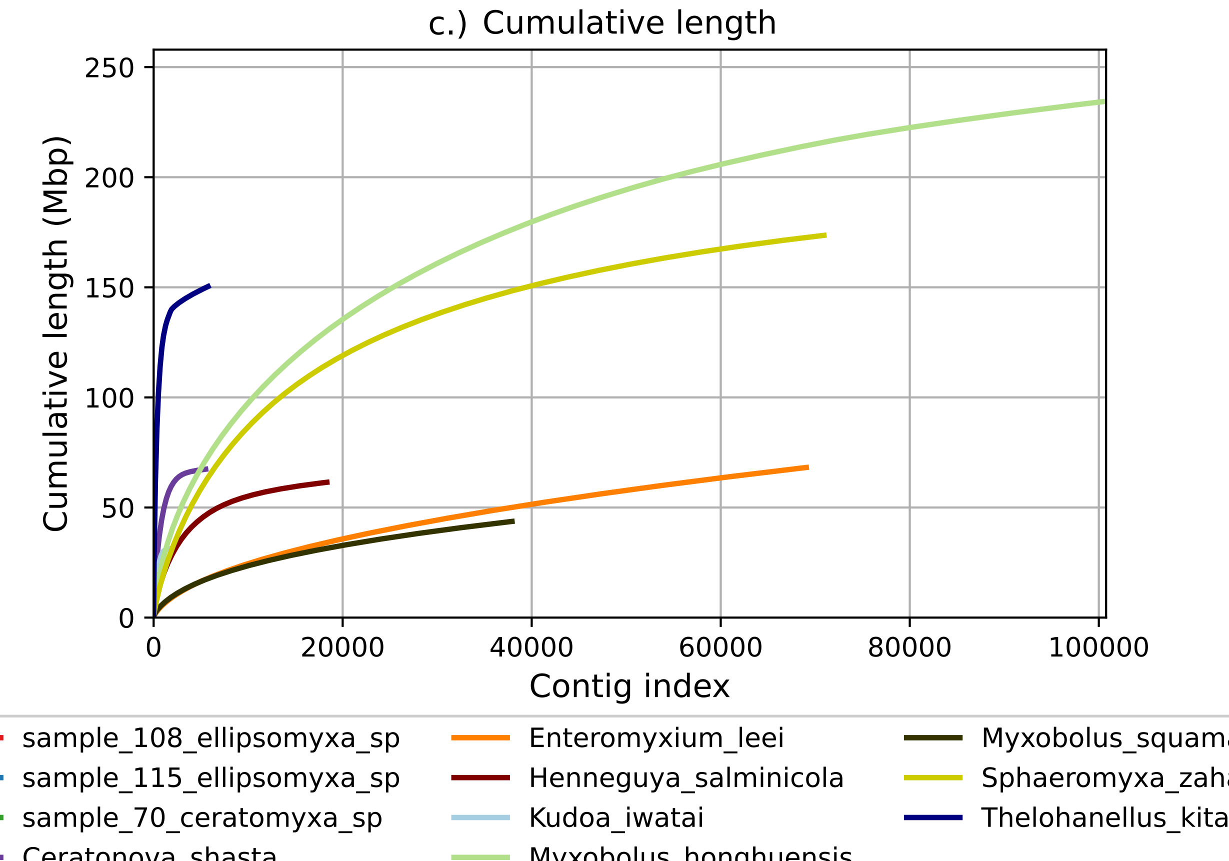
c.) Cumulative length
Cumulative length (Mbp)
0
50
100
150
200
250
0
20000
40000
60000
80000
100000
Contig index
sample_108_ellipsomyxa_sp
sample_115_ellipsomyxa_sp
sample_70_ceratomyxa_sp
Enteromyxium_leei
Henneguya_salminicola
Kudoa_iwatai
Myxobolus_squama
Sphaeromyxa_zaha
Thelohanellus_kita

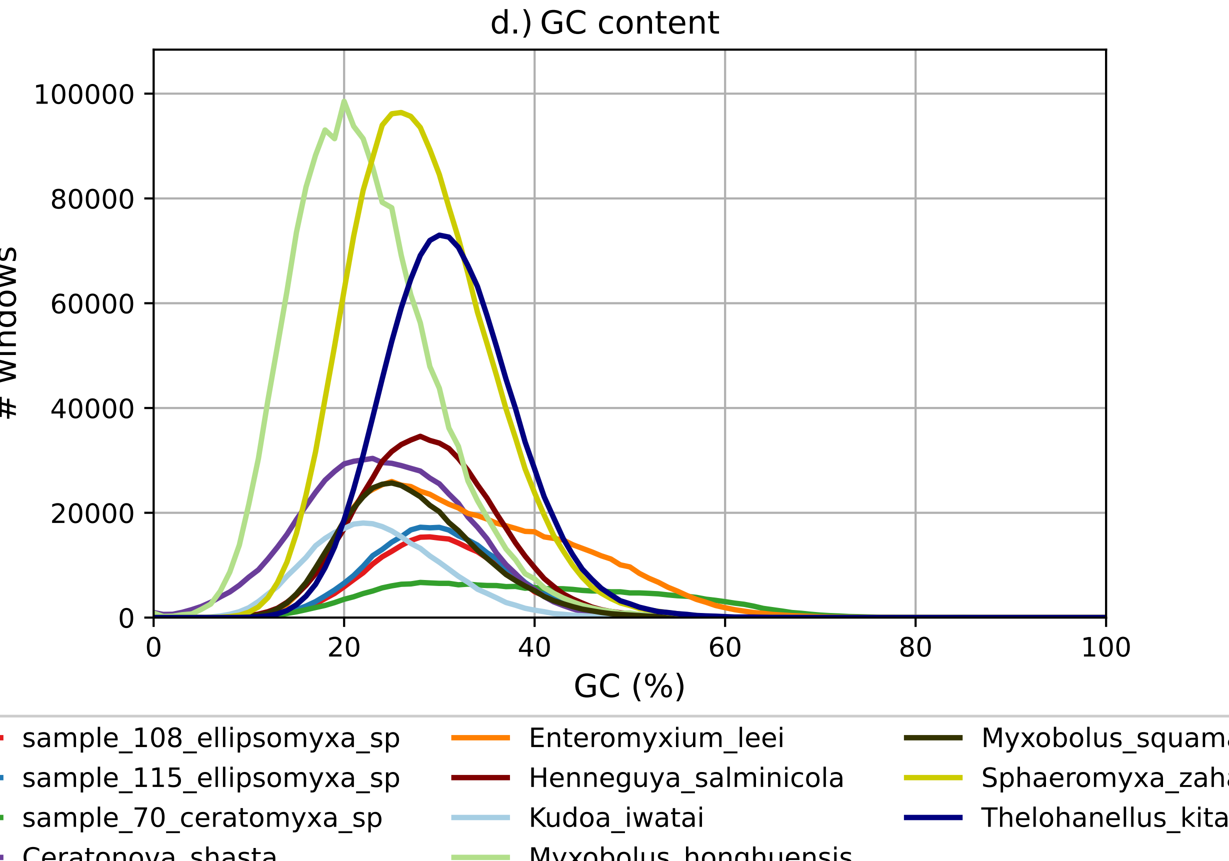

d.) GC content
windows
100000
80000
60000
40000
20000
0
0
20
40
60
80
100
GC (%)
sample_108_ellipsomyxa_sp
sample_115_ellipsomyxa_sp
sample_70_ceratomyxa_sp
Enteromyxium_leei
Henneguya_salminicola
Kudoa_iwatai
Myxobolus_squama
Sphaeromyxa_zaha
Thelohanellus_kita

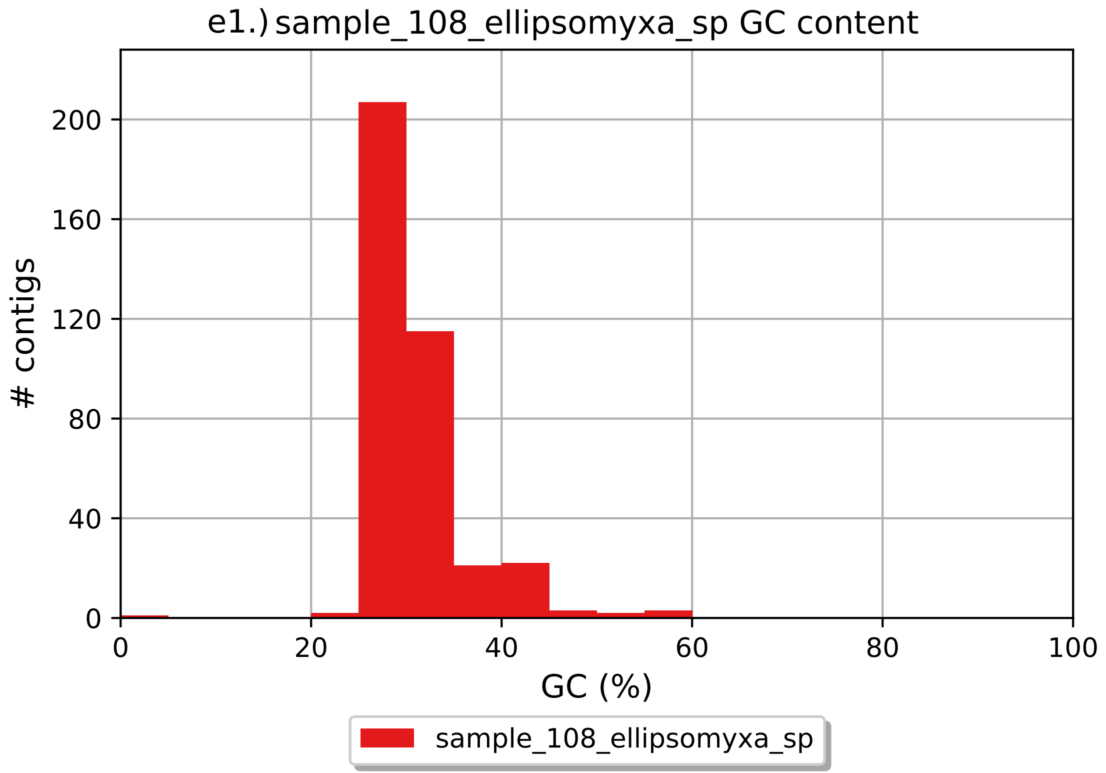

e1.) sample_108_ellipsomyxa_sp GC content
contigs
0
40
80
120
160
200
0
20
40
60
80
100
GC (%)
sample_108_ellipsomyxa_sp

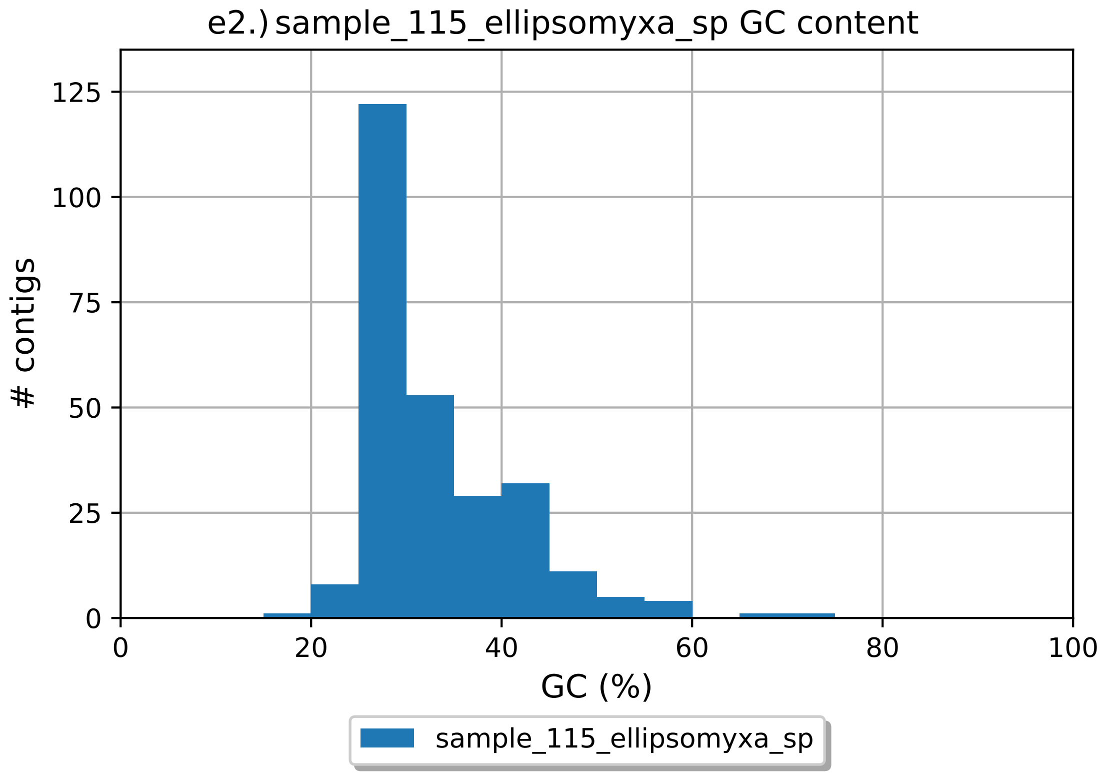
e2.) sample_115_ellipsomyxa_sp GC content
contigs
125
100
75
50
25
0
0
20
40
60
80
100
GC (%)
sample_115_ellipsomyxa_sp

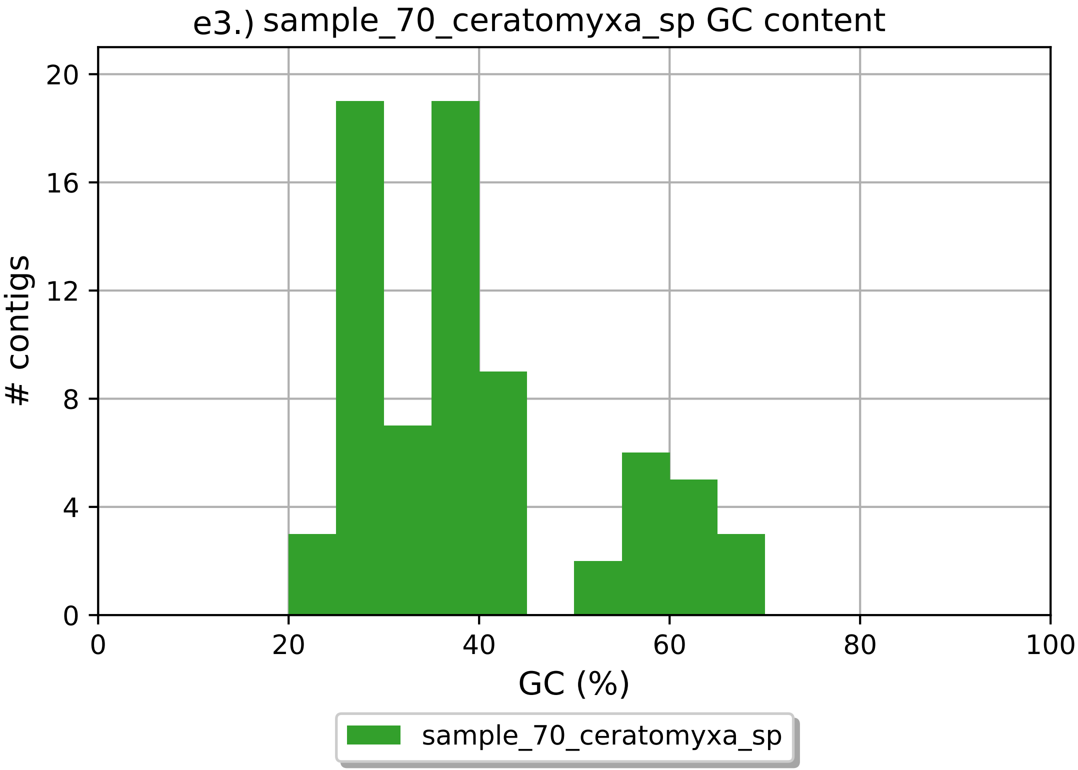

e3.) sample_70_ceratomyxa_sp GC content
contigs
0
4
8
12
16
20
0
20
40
60
80
100
GC (%)
sample_70_ceratomyxa_sp

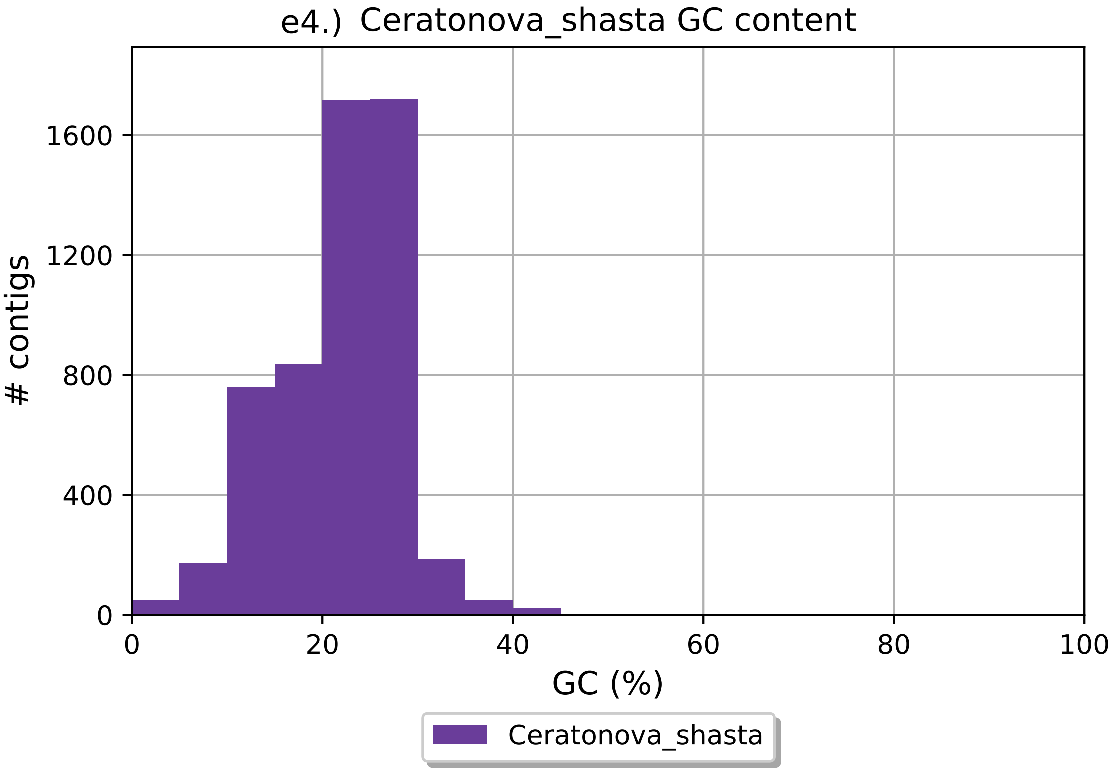

e4.) Ceratonova_shasta GC content
contigs
0
400
800
1200
1600
0
20
40
60
80
100
GC (%)
Ceratonova_shasta

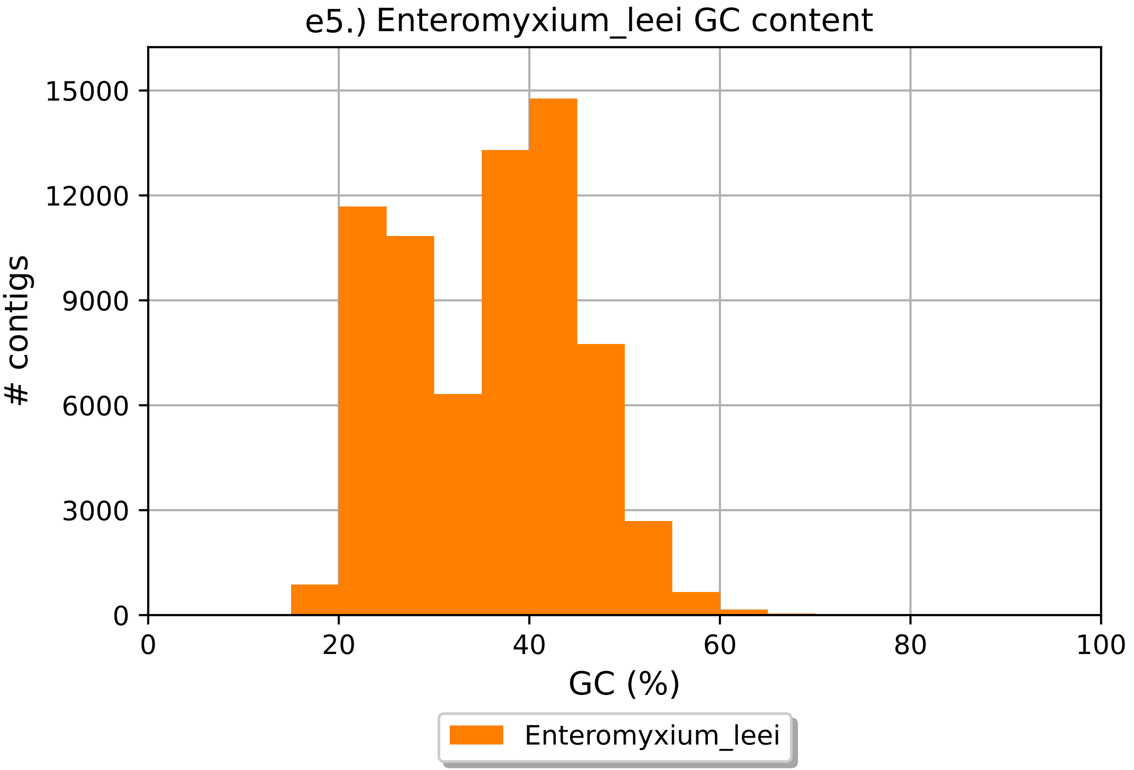
e5.) Enteromyxium_leei GC content
contigs
15000
12000
9000
6000
3000
0
0
20
40
60
80
100
GC (%)
Enteromyxium_leei

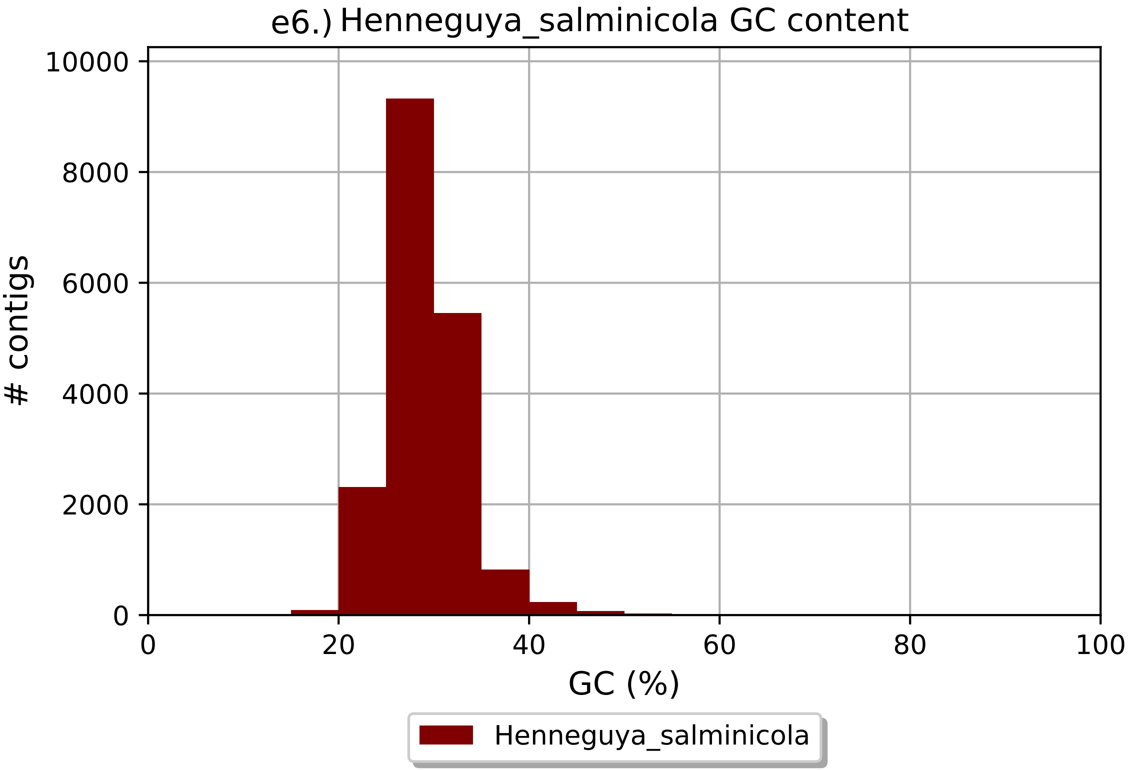

e6.) Henneguya_salminicola GC content
contigs
0
2000
4000
6000
8000
10000
0
20
40
60
80
100
GC (%)
Henneguya_salminicola

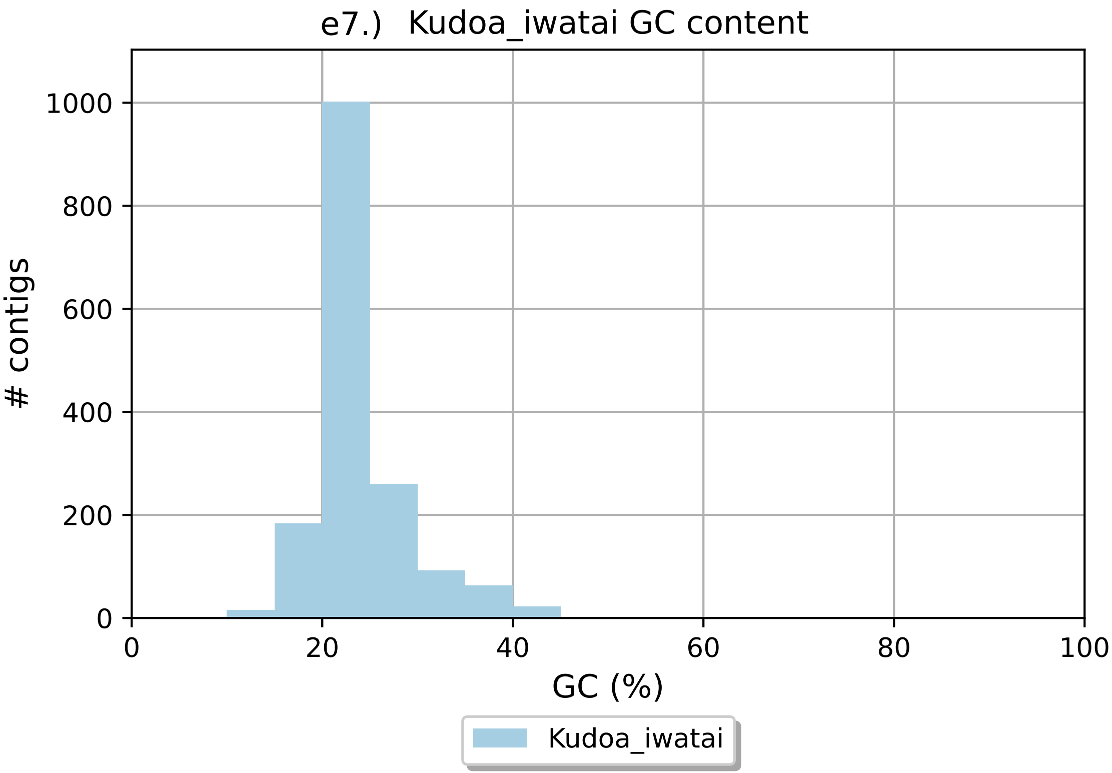

e7.) Kudoa_iwatai GC content
contigs
GC (%)
Kudoa_iwatai
0
200
400
600
800
1000
0
20
40
60
80
100

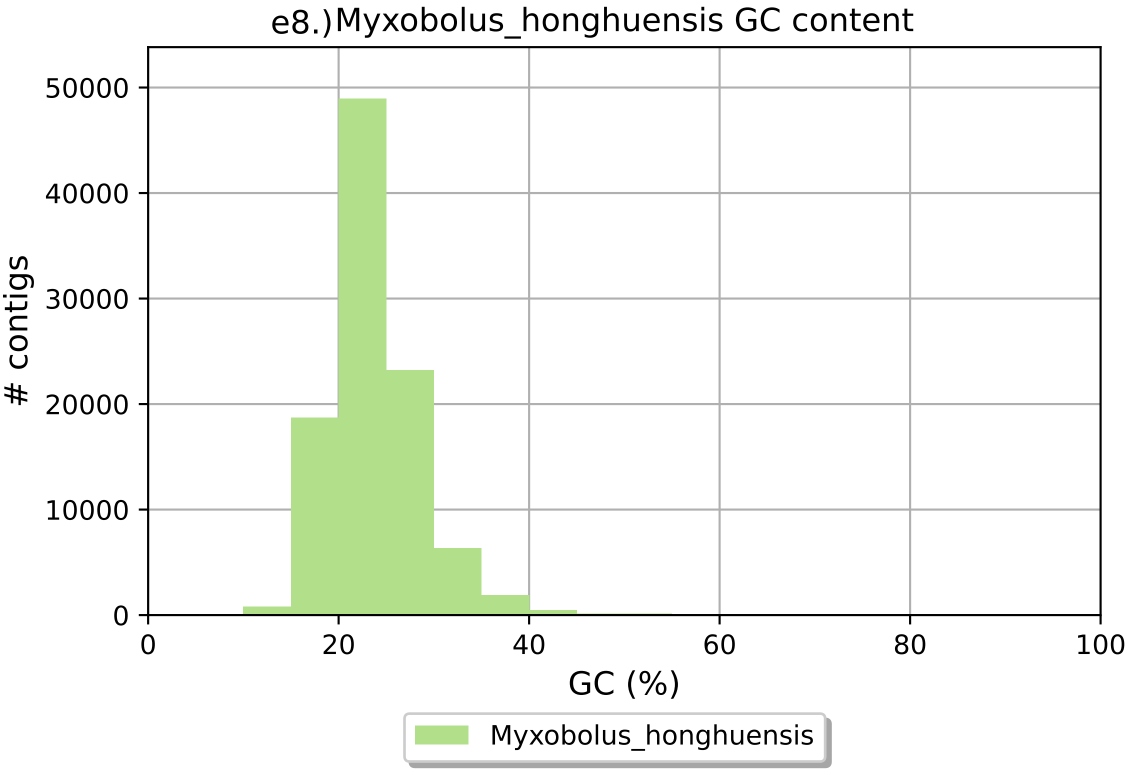
e8.)Myxobolus_honghuensis GC content
contigs
GC (%)
0
10000
20000
30000
40000
50000
0
20
40
60
80
100
Myxobolus_honghuensis

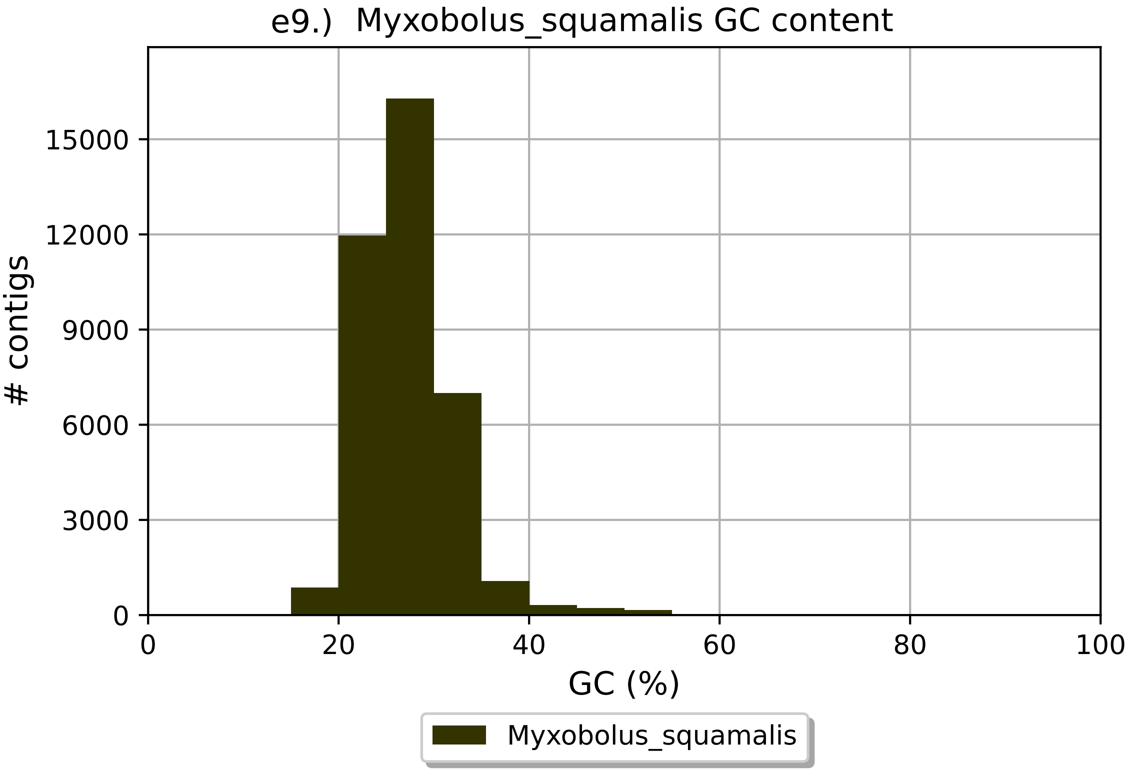
e9.) Myxobolus_squamalis GC content
contigs
GC (%)
0
3000
6000
9000
12000
15000
0
20
40
60
80
100
Myxobolus_squamalis

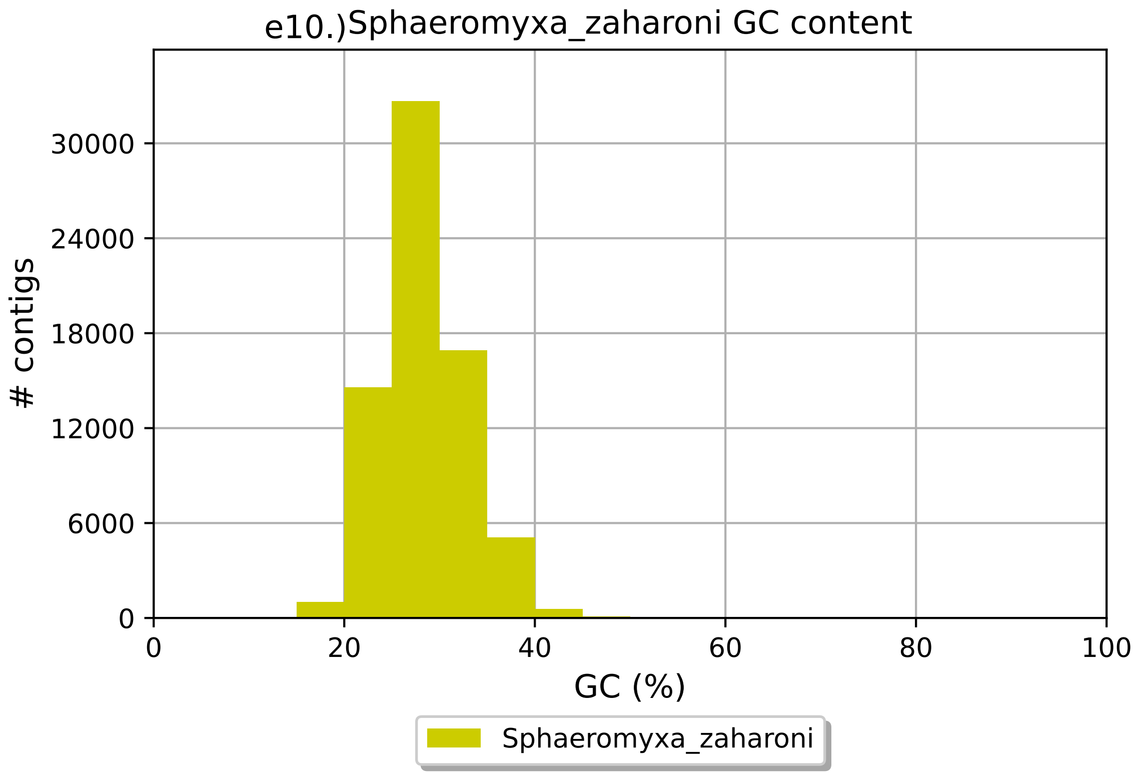

e10.)Sphaeromyxa_zaharoni GC content
contigs
30000
24000
18000
12000
6000
0
0
20
40
60
80
100
GC (%)
Sphaeromyxa_zaharoni

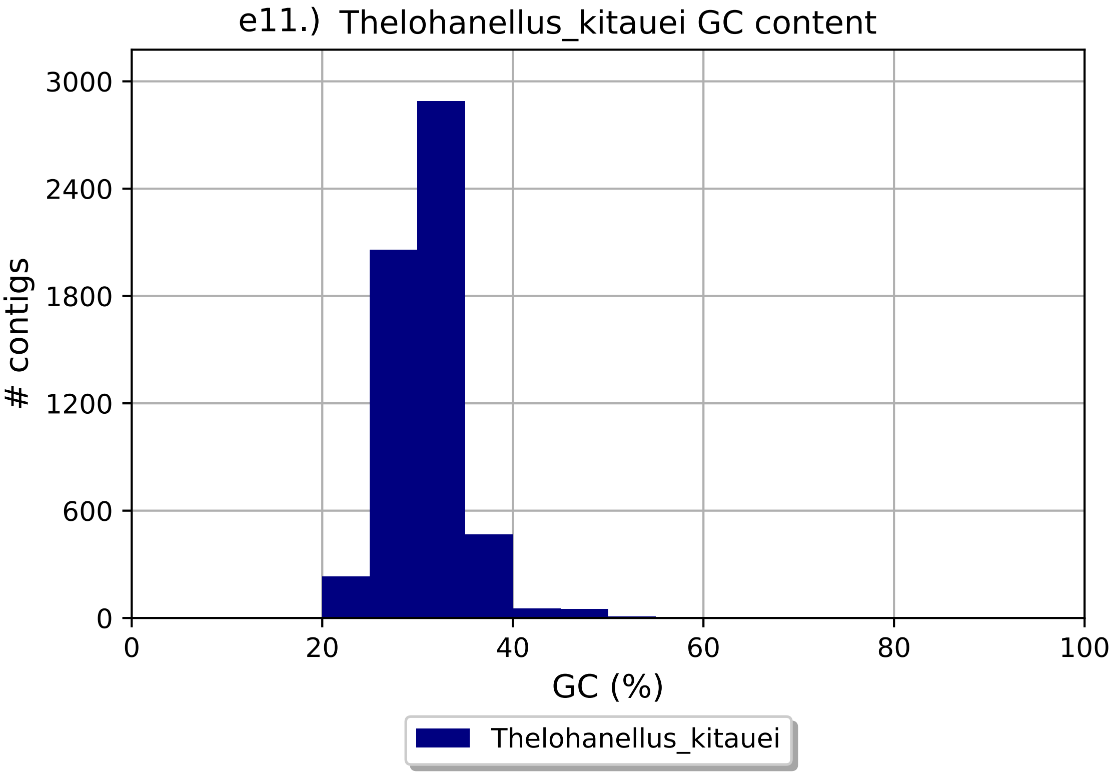
e11.) Thelohanellus_kitauei GC content
3000
2400
1800
1200
600
0
contigs
0
20
40
60
80
100
GC (%)
Thelohanellus_kitauei
