# Supplementary material for: Long-read metagenomic sequencing negates inferred loss of cytosine methylation in Myxosporea (Cnidaria: Myxozoa)
Source: Gigascience. 2025 Mar 13;14:giaf014. doi: 10.1093/gigascience/giaf014 (PMC11905887; doi:10.1093/gigascience/giaf014)
Supplement: giaf014_Supplemental_Files [file giaf014_supplemental_files.zip › Supplementary File 8_Genome coverage plot Sample 70.docx]

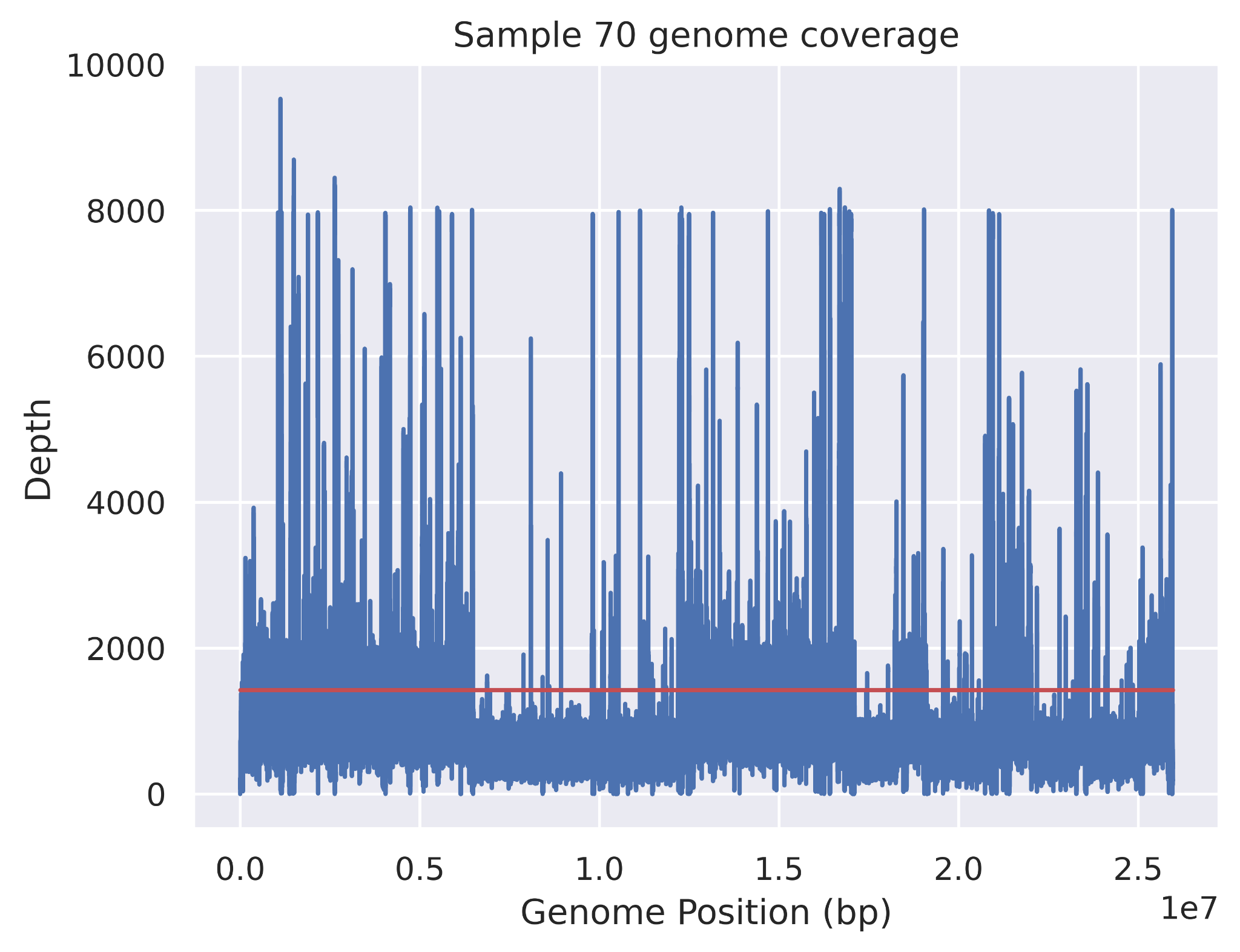


Supplementary File 8: Sample 70 genome coverage plot produced using Seaborn library for making statistical graphics in Python. The figure represents a histogram produced from a bedgraph file for Sample 70 genome assembly. Using Minimap2 with “map-ont” enabled, all fastq_pass reads have been mapped onto the filtered assembly and afterwards a bedtools coverage tool was used to compute both the depth and breadth of coverage based on the Minimap2 .bam mapping file. Median coverage obtained was 1424, with maximal coverage of 9530. A negligible percentage of 0.00069% bases in the assembly has no reported coverage.
