# Supplementary material for: Long-read metagenomic sequencing negates inferred loss of cytosine methylation in Myxosporea (Cnidaria: Myxozoa)
Source: Gigascience. 2025 Mar 13;14:giaf014. doi: 10.1093/gigascience/giaf014 (PMC11905887; doi:10.1093/gigascience/giaf014)

A word cloud analysis to determine which topics are most common across the 11 genomes in each gene ontology category - A) Molecular functions, B) Biological process and C) Cellular component, being conserved across all the 11 Myxozoa genomes studied.

1. Molecular functions


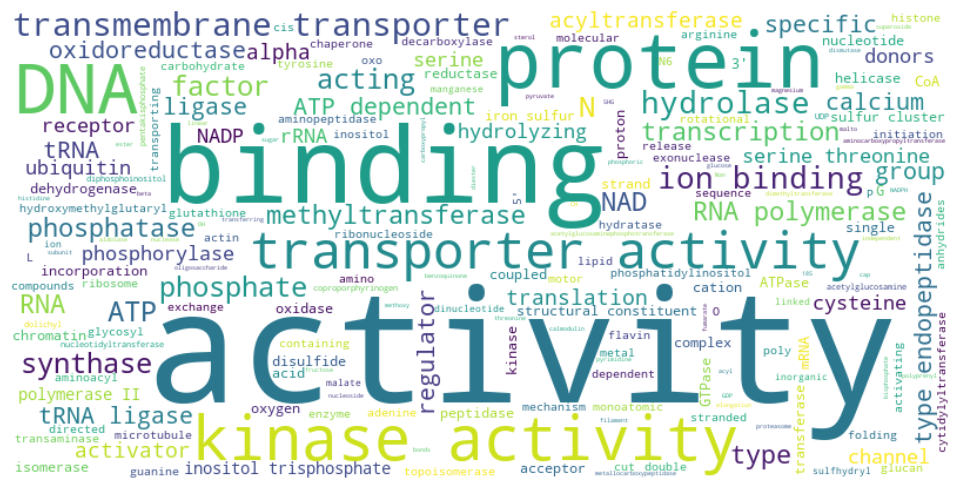


1. Biological process


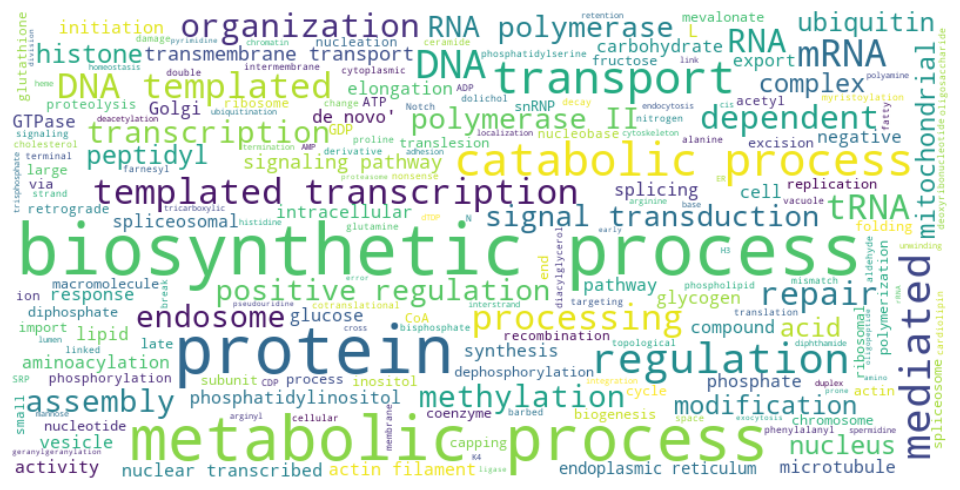


1. Cellular component


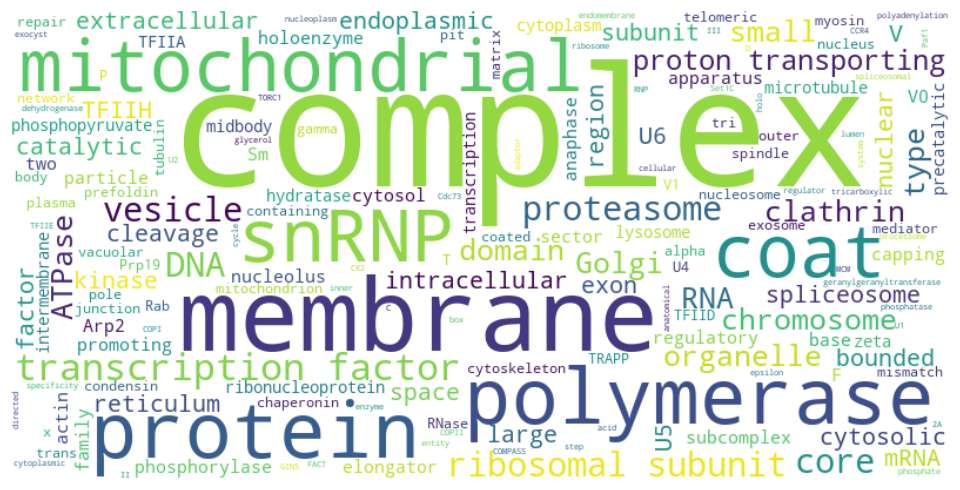

Supplement: giaf014_Supplemental_Files [file giaf014_supplemental_files.zip › Supplementary File 9_Word cloud analysis.docx]
