# Supplementary material for: Long-read metagenomic sequencing negates inferred loss of cytosine methylation in Myxosporea (Cnidaria: Myxozoa)
Source: Gigascience. 2025 Mar 13;14:giaf014. doi: 10.1093/gigascience/giaf014 (PMC11905887; doi:10.1093/gigascience/giaf014)
Supplement: giaf014_GIGA-D-24-00150_Revision_1 [file giaf014_giga-d-24-00150_revision_1.pdf]

GigaScience

Long read metagenomic sequencing negates inferred loss of cytosine methylation in Myxosporea (Cnidaria: Myxozoa)

--Manuscript Draft--

**Manuscript Number:**	GIGA-D-24-00150R1
**Full Title:**	Long read metagenomic sequencing negates inferred loss of cytosine methylation in Myxosporea (Cnidaria: Myxozoa)
**Article Type:**	Research
**Funding Information:**	King's College London (AC15137) — Prof Edson A Adriano; Professor Paul F Long<br>Fundação de Amparo à Pesquisa do Estado de São Paulo (2019/17427-3) — Prof Edson A Adriano; Professor Paul F Long<br>Coordenação de Aperfeiçoamento de Pessoal de Nível Superior (Finance Code 001) — Prof Edson A Adriano<br>Conselho Nacional de Desenvolvimento Científico e Tecnológico (307485/2023-4) — Prof Edson A Adriano<br>Fundação de Amparo à Pesquisa do Estado de São Paulo (2023/06420-3) — Ms Rayline T. A. Figueredo<br>Fundação de Amparo à Pesquisa do Estado de São Paulo (18/24980-8) — Prof Edson A Adriano
**Abstract:**	Oxford-Nanopore PromethION sequencing is a PCR-free method that retains epigenetic markers and provides direct quantitative information about DNA methylation. Using this long-read sequencing technology, we successfully assembled five myxozoan genomes free from discernible host DNA contamination, surpassing previous studies in both quality and completeness. Genome assembly revealed DNA methylation patterns within myxozoan genomes, particularly in GC-rich regions within gene bodies. The findings not only refute the notion of myxozoans lacking DNA methylation capability but also offer a new perspective on gene regulation in these parasites. The high-quality genome assemblies lay a solid foundation for future research on myxozoans, including new strategies to control these commercially significant fish pathogens.
**Corresponding Author:**	Paul F Long<br>King's College London<br>LONDON, UNITED KINGDOM
**Corresponding Author Secondary Information:**	
**Corresponding Author's Institution:**	King's College London
**Corresponding Author's Secondary Institution:**	
**First Author:**	Antonio Starcevic
**First Author Secondary Information:**	
**Order of Authors:**	Antonio Starcevic
	Rayline T. A. Figueredo
	Juliana Naldoni
	Lincoln L Corrêa
	Edson A Adriano
	Beth Okamura

Powered by Editorial Manager® and ProduXion Manager® from Aries Systems Corporation

	Paul F Long
**Order of Authors Secondary Information:**	
**Response to Reviewers:**	Reviewer #1:<br><br>Comment 1. Does the selected sample sufficiently represent the genetic diversity of the target Myxozoa population? Could the authors elaborate on the criteria and rationale for sample selection?<br>We find this comment somewhat obscure. Our aim was to detect whether there is evidence for methylation in myxozoans, not to explore genetic diversity in populations. Our overall ‘target population’ was therefore the 5 genomes we sequenced plus the myxozoan genomes already available from other studies. In terms of the material we specifically collected, we have shown that genome sequences for samples 70, 108 and 115 are different from each other and from samples 57 and 58, which are biological replicates. Hence, the data we have generated for this study does represent genetic diversity in the population sampled from Amazonian fish hosts. We acknowledge that we did not provide information on how we ‘selected’ the samples, but we have now addressed this by adding the following text on page 8 of the Methods:<br>‘ Our aim was to sample opportunistically for myxozoans in Amazonian fish gall bladders, which have been commonly infected by these cnidarian parasites (44). After the dissection of each fish the gall bladder was removed and a sterile scalpel blade was used to open the gall bladder pouring the bile directly into a sterile 1.5 mL Eppendorf tube. With a sterile micro-pipette, a drop of the bile fluid was immediately placed on a microscope slide and examined using a light microscope (Carl Zeiss model Primo Star) at 20x or 40x to ascertain infection. When infection was confirmed, the bile fluid content of the Eppendorf tube was immediately preserved in DNA/RNA Shield reagent (Zymo Research Corp., Irvine, CA, USA).<br>And to page 19 of the Results where we discuss sampling fish hosts:<br>‘We detected myxozoan infections in 102 out of 189 fish gall bladders (54%). The DNA of myxozoans collected from the bile of five fish specimens was isolated in sufficient concentration and purity required for ONT sequencing. Just over 9 terabytes of sequencing data were generated.’<br><br>Comment 2. Based on genomic synteny analysis, how do the researchers ascertain the homology relationships between samples? Is there a possibility of mixed infections or other complex scenarios that could influence this determination?<br>In addition to genomic synteny analysis, we did extract 18S rRNA genes and used these for both taxonomic placement of our samples, and to assess the overall genomic homology between samples. Contamination with host DNA is inevitable, and to assess the extent of this in our samples, we developed a stringent quality criterion in which we screened both our assembled genomes and the ONT long reads to “fish out” 18S rRNA genes that we could then analyze using the latest Silva (SILVA Release 138.2) SSU NR portion of the database. This analysis confirmed that our genome assemblies indeed represented single myxozoan species in various degrees of completeness, with the only other detectable SSUs coming from bacteria, fish hosts and human contamination. Since submission of our manuscript, the genomes assemblies have also passed the NCBI host contamination filtering process and are now deposited with their own individual accession numbers, which have been included in the data availability statement on page 41.<br><br>Comment 3. The manuscript mentions the generation of over 9TB of sequencing data using Oxford Nanopore PromethION technology and steps taken to mitigate host DNA contamination during data analysis. However, the original proportion of host to myxozoan DNA in the samples is not detailed. Clarification on this proportion is essential for understanding the complexity of the samples and the accuracy of subsequent analyses.<br>Host DNA contamination is inevitable in all myxozoan sequencing attempts reported to date due to the endoparasite lifestyle of these animals. It is accepted that Myxozoa have undergone quite drastic genome reduction whereas fish hosts, on other hand, have likely had whole-genome duplication events during their long evolutionary histories. This inevitably leads to imbalanced genomic datasets. For our data, parasites are the minority class, while the fish hosts represent the majority. Acknowledging this, we have used well established imbalance dataset training reasoning and applied this during genome assembly. In particular, we have used under-sampling of all host

related reads when preparing our assembly datasets. Furthermore, after resolving the assembly contigs, we used BlobTools and, based on several comprehensive BLAST searches, judiciously removed all suspicious contigs.
We have included the following text to page 21 of the Results:
Overall, this produced satisfactory results despite the initial imbalance ranging from 10 to 1 in favor of host DNA in Ellipsomyxa sp. sample 115, up to 4 to 1 in Ceratomyxa sp. sample 58. The only sample that had relatively more parasite reads compared to the host was Ellipsomyxa sp. sample 70, with a 2 to 1 ratio in favor of parasite DNA. These calculations were based on ratios between excluded reads (i.e., ones that mapped onto reference fish genomes) and reads that mapped onto contamination pruned assemblies.
We have included the following text to page 32 of the Discussion:
Although the ratio of host to parasite reads is important to mitigate host contamination, in our experience when handling ONT sequencing datasets the actual quantity of DNA sequencing template has a much greater impact on the final completeness of the genome assemblies. compared to Illumina based NGS. This is because the read quality and read length impacts subsequent analyses, and we found these to be variable amongst the samples. For example, Ellipsomyxa sp. sample 70 had significantly longer reads of better quality on average, when compared to the rest of the samples.

Comment 4.The potential for genomic contamination is acknowledged in the study, but its impact on the results is not thoroughly discussed. How did the researchers assess the extent of contamination, and how might it affect the interpretation of the myxozoan genomes?
We thank the referee for this comment and address this by adding the following text to page 32 of the Discussion:
Host DNA contamination is almost inevitable in all Myxozoa sequencing attempts due to the endoparasite lifestyle of the animals. This will lead to false interpretation of Myxozoa genome sizes and introduce confusion in understanding of evolutionary relationships due to very diverse host ranges. To reduce the extent to which this contamination might affect interpretation of myxozoan genomes was reduced herein by using minimap2 to map all reads onto a large set of reference fish genomes, and then assembling the non-mapping reads. These assembled contigs were then screened by BLAST comparison against comprehensive databases such as NT and tsa_NR in order to remove even the slightest remaining homologies to fish hosts.

Comment 5. While Nanopolish was utilized for DNA methylation detection, considering the limitations mentioned for non-targeted WGBS in myxozoan genomic studies, could the authors consider targeted capture WGBS as a validation method? Given the low methylation levels observed in sample 70, have additional experiments been conducted to ensure these signals are not false positives from CpG site identification by Nanopore?
The referee is correct, WGBS is used as a validation method for ONT based methylation calling. There are even readily available scripts provided by ONT for this task: https://nanopolish.readthedocs.io/en/latest/quickstart_call_methylation.html. However, we were unable to perform such a validation test in this study. This was because sample collection was in a very remote location, and we could not perform additional sampling nor could we preserve sufficient quantities for repeated studies and avoid DNA degradation. Therefore, we have used a biological duplicate in case of samples 57 and 58, which contain the same species of Myxozoa, from the same fish host, sampled independently and processed separately. Using these samples, we performed the same validation as would have done using WGBS as a control. In a cross-reference manner, akin to a bi-directional blast approach, we mapped reads from sample 57 onto sample 58 assembly and vice-versa, whilst performing methylation calling in both cases. The resulting methylation frequencies per site were compared and a correlation statistic calculated. The results obtained were comparable to most studies using WGBS (as a control), so we are confident in their accuracy. Sample 70 is our best genomic sample, based on multiple criteria (assembly completeness, read quality, coverage). Due to very high read coverage (>300x), methylation frequencies inferred from this sample data are as reliable as one can get. Each methylated/unmethylated CpG location was assessed based on at least 300 individual ONT read signals encoded in fast5 files, making the probability of false positives/negatives virtually non-existent. Regarding the low methylation level in this

sample, this is comparable to methylation levels in samples 108 and 115, and is in line with previous work by Kyger et al. We believe the stark differences in levels of genome methylation might be explained by the stage of the life cycle when the DNA was extracted. We have mentioned in the discussion that further work needs to be done to prove this experimentally, but it certainly opens new and challenging avenues of research in Myxozoa.

Comment 6. The manuscript refers to the CpG Observed/Expected (O/E) ratio as an indicator of DNA methylation, but the specific biological implications and influencing factors of this ratio in different genomic regions, such as CDS and non-CDS, are not fully explored. Could the authors further elucidate the biological significance of the CpG O/E ratio and its role in gene regulation?
CpG O/E values were used as the input for MethMod - a Gaussian Mixture Model (https://github.com/JanLeipzig/MethMod/) to either prove or disprove the existence of epigenetic capabilities of various invertebrates based solely upon genome data. In this regard, our samples displayed a methylation associated pattern of CDS CpG O/E values consistent with other invertebrates known to methylate their DNA.
We have added the following text to page 36 of the Discussion to explain the biological significance of CpG O/E patterns:
Determining the overall genomic CDS CpG O/E ratio is a very significant indicator of DNA methylation and in essence is quite a simple concept. Any CDSs that are often methylated will inevitably display markedly lower CpG O/E values than sequences that do not. This is due to an increased rate of mutation in methylated CpG that leads to GC content loss, because C-to-T transitions are the most common type of mutation at these sites. In terms of resultant distribution, this GC loss will lead to a characteristic two-peak bimodal distribution which is a characteristic that has been observed in all invertebrate species known to methylate DNA (Figure 8). Additionally, when the distribution of the ratio between CDS and non-CDS regions of the genomes were compared, most suspected methylation events occurred in CDS regions as expected, since this is a tell-tale mark of invertebrate methylation (unlike in vertebrates which skip non-CDS regions). The GC content has significant biological consequences reflected in gene inactivation and genome instability that could explain reported variation in Myxozoa genome sizes, and overall high fragmentation and difficulties in assembly and annotation of genomes.

Comment 7. The use of Kernel Density Estimation (KDE) analysis to reveal a bimodal methylation pattern is intriguing, yet the biological significance of this pattern is not clearly explained. Could the authors provide more detail on how the KDE analysis supports the existence of two distinct methylation states in CDS populations and the potential impact of this finding on understanding gene regulatory mechanisms in myxozoans?
KDE was used as a fully non-parametric method to prove the existence of two distinct gene sub-populations typical of methylation patterns observed in invertebrates, without having to assume a particular distribution is present. The KDE results clearly indicated the presence of a bimodal distribution composed of one cluster of CDSs with lower CpG O/E values, and second with higher CpG O/E values - which indicates presence of preferably methylated and preferably unmethylated CDSs within the same genome (Figure 8). In addition, KDE provides a more flexible and robust method for estimating density functions, especially when the underlying distribution is not strictly Gaussian, which is a possibility we needed to consider given that Myxozoa are known to display genome reduction. By confirming the bimodal pattern using KDE, we strengthened the evidence for the existence of two distinct methylation states by analyzing CDS populations, and validated results obtained using MethMod. However, we must say that this does not reveal anything on the underlying methylation mechanisms except confirming that they exist. One additional thing we did was to implement weighted KDE, where we used epigenetic data and ONT nanopore based methylation frequency results in its full reported range as weights, and genomic based CpG O/E values as data points. Adding weights did not change the distribution, which was still bimodal, nor did it significantly shift the low CpG O/E cluster peak. However, it did highlight the low CpG O/E cluster that is being methylated and shifted its peak to a lower value. Using epigenetic data as weight has also inverted the peak heights that represent probability density of observing a data point at that specific value. This allowed us to make more precise CpG O/E boundaries that can be used to pinpoint which CDSs are being actively methylated, and which are not and it also confirmed our hypothesis that large

Powered by Editorial Manager® and ProduXion Manager® from Aries Systems Corporation

number of gene bodies are being methylated/demethylated in an unison manner, hence such an inversion of peak heights with a methylation frequency weighted KDE compared to unweighted one based solely on genome level CpG O/E data.
We have added the following text to page 37 of the Discussion:
The combination of epigenetic data with the CpG O/E value calculated from the genome assemblies in the form of a weighted KDE, more precisely defined CpG O/E boundaries and additionally more accurately pinpointed which CDSs were being actively methylated. We believe this is the first study to use epigenetic methylation frequency results in combination with genomic derived CpG O/E values to assess the frequency of methylation at specific genetic loci. This approach has potential as a preliminary screening method to identify genes of interest for targeted experimental approaches to resolve epigenetic processes, including regulatory mechanisms.

Comment 8. The manuscript proposes a "transcription switch" mechanism for gene expression regulation in myxozoan genomes. To substantiate this hypothesis, has consideration been given to quantitative PCR (qPCR) experiments to compare gene expression levels under varying methylation conditions? If feasible, could experiments be designed to measure transcription levels of CDSs with high and low CpG O/E values to determine if methylation status correlates with gene expression repression or activation? Such experiments would provide functional evidence for the "transcription switch" model and contribute to understanding the role of DNA methylation in gene regulation in myxozoans.
We thank the referee for their comment and fully agree that additional work needs to be done to substantiate our hypothesis. However, this is outside of the scope of the work described herein, which was to determine if DNA methylation exists in Myxozoa. Therefore, we have now omitted this “transcription switch” hypothesis from the text, including the title and have rewritten the abstract. This hypothesis has been fueled by stark difference between samples 57/58 and the rest of the samples methylation frequency findings we report herein. The weighted KDE method as explained above has further strengthened our initial suggestion that the most plausible explanation of this experimentally confirmed difference would be a most rudimentary mechanism in which a large number of gene bodies are being methylated/demethylated in unison, hence the “switch” parallel. Using experimental evidence we obtained (methylation frequencies) and “theoretical” data - CpG O/E values of CDSs, we were able to provide additional evidence for this, but as you noted further work is a proposition we shall certainly undertake in near future.

Reviewer #2:
Comment 1. 'transcription switch' mechanism: The main assertion of the paper states that “experimental and in silico analyses revealed variable methylation levels genomes, suggesting a 'transcription switch' mechanism regulating gene expression,” as noted in the abstract. However, there appears to be a significant gap between sequence analysis and the proposed mechanism. Could the authors provide additional validation for this finding through cross-platforms sequencing, such PacBio HiFi and WGBS? Although the authors mention associated with WGBS, incorporating data from multiple sequencing technologies could strengthen their conclusions. Furthermore, given that most assembled genome CpG sites are methylated, suggesting that many genes may be transcriptionally silenced, transcriptomic analyses could serve as compelling evidence for this finding. Additionally, the identification of DNA methyltransferase (DNMT), ten-eleven translocation methylcytosine dioxygenase (TET), and base excision repair (BER) enzymes warrants further investigation. A comparative analysis of these proteins with their homologys, particularly focusing on their functional domains, would be meaningful to support the authors’ conclusions.
We fully agree with the referee that our findings need to be further validated, including cross-platform sequencing and comparative analysis of proteins related to methylation mechanisms. However, as we have provided in response to Referee 1 Comment 8, these experiments are outside of the scope of the current manuscript, which aimed to determine if DNA methylation occurs in Myxozoa. Also, as far as we are aware, it is only ONT nanopore that has the capability of addressing both base-calling and methylation calling tasks in a single run, and this is the reason why it was used here. Cross-platform sequencing could be used to improve existing genome assemblies and indeed we are already planning to use it for continuation of the work described here. Regarding your comment on CpG sites, where you stated that “most assembled genome CpG sites are methylated”, in our results section we in fact reported the

Powered by Editorial Manager® and ProduXion Manager® from Aries Systems Corporation

opposite of this. As we clearly stated and provided sufficient experimental evidence (ONT recorded methylation calls, additionally validated through one biological replicate), only sample 57 and 58 (the biological replicate) provided this outlook where most CpG sites within genome bodies has been extensively methylated, while the remainder of samples - 108, 115 and sample 70 were total opposites, with only a minor fraction of CDSs showing signs of CpG methylation. This has led us to name this putative mechanism 'transcription switch'. Now when looking at this expression which we came up with, in the light of your very insightful comments, we believe “epigenetic switch” might have been a more proper phrase. Nevertheless, concurrent with your comments and Referee 1 Comment 8, we have removed the concept of a transcriptional switch from the manuscript, including the title and have re-written the abstract.

Comment 2. Pangenome Analysis: The utilization of pangenome analysis is generally intended to assess genetic diversity within populations of a single species or closely related species. However, as this study encompasses several different myxozoan species, the relevance of a pangenome approach is questionable. The authors should consider reframing this section as “genome comparison” if no specific pangenome graphs or structural variants are presented.

We completely with the referee’s comment and have rephrased throughout the manuscript and figures.

Comment 3. Word Cloud Analysis for GO Terms: While I understand the rationale behind using a word cloud analysis to highlight overrepresented protein functions within the core gene repertoire, this may not be necessary because no strong relationship with your propose. GO term comparisons can often be too vague to provide specific insights into study objectives, particularly for this research.

After carefully assessing your comment, we have come to the conclusion that our explanation of MethMod and KDE related analyses might have been too vague, and we sincerely apologize for this. We believe this might have obscured the actual intent behind our GO work presented. The major reason why we performed the word cloud analysis was not to highlight overrepresented protein functions, since we performed no transcriptomics or proteomics in this research. The reasoning behind the GO based word cloud analysis was to display the most prominent functions accumulated in what we believe are preferably methylated gene bodies and preferable non-methylated ones. These groups (or clusters) have been determined based on extensive modeling effort, first using MethMod parametric Gaussian mixture modeling and followed by non-parametric KDE, in order to validate and confirm our findings and grouping of the gene bodies in preferably methylated/unmethylated clusters. As described above, we went one step further, by combining epigenetic and genetic markers - the methylation frequencies of CpG sites within gene bodies, with the gene bodies probabilistic based CpG O/E values, that we used to assess long-term exposure of these gene bodies to methylation/demethylation events. You are right to state that this might be inconclusive, since obviously no proteomics or transcriptomics data has been presented, however as you also noted, there is a strong rationale behind these word clouds, they were based on strong statistics and our intention was to suggest future transcriptomics and proteomics based work.

Comment 4. Claims Regarding Database Matches: In the abstract, the authors state, “These genomes did not match any existing sequences in public databases, suggesting a need for updated sequence data for these elusive parasites.” This claim appears exaggerated potentially misleading, as I could not find where this assertion is supported in the manuscript. I recommend rephrasing this statement and including additional discussion to substantiate it.

We agree with the referee’s comment. This sentence has been removed from the abstract.

Comment 5. Genome Completeness: The authors performed assembly quality assessments, including N50, N90, BUSCO analyses. However, the BUSCO results alone cannot substantiate the claim of the genome being “complete” (sample 70), as it only covered half of the complete BUSCOs (123 out of 255), and the estimated genome size is considerably smaller that of other previously sequenced myxozoans, which range from 61.44 to 234.48 Mbp. I suggest the authors directly map the ONT reads to their assembly to measure genome quality and provide a quality value (QV). Furthermore, I caution in the use of the term “complete” when discussing genome assembly.

We have changed “complete” into “best quality” throughout the text in order to make this statement more acceptable. We do agree that no available metrics is sufficiently

good enough to be able to claim genome completeness. It was not our intent to claim we have a “complete” genome, it was our intent was to state that we have “the most complete” genome when compared to previously assembled myxozoan genomes. Based on these metrics we have presented; we believe this is accurate.
Regarding the BUSCO completeness, this is especially difficult to address since these parasites are known to undergo genome reduction, so we did not expect to obtain a full set of BUSCOs, or to assess how many might be retained. Regarding your suggestions on mapping, we performed multiple rounds of ONT read mappings onto each of our genomes. This was extensive work, and it is included in our genome assembly pipeline as well as species confirmation.
To clarify, here is what was exactly done in this regard: In the read filtering stage, as part of the host contamination removal, we have mapped all fastq reads onto reference fish genomes and used only the unmapped ones for the subsequent genome assemblies. The reasoning behind this being the fact that unmapped reads are obviously enriched with the parasite DNA. After performing the Flye based assembly, we have evaluated obtained genome sizes using kmer counts, as described in the manuscript, but we have also remapped all reads back onto the alignment in order to assess coverage (whether all assembled contigs are evenly covered) and also as part of the BlobTools removal of suspicious contigs that might harbor some contamination that we failed to remove in previous step. BlobTools provides interactive quality assessment based exactly on the mapping you are mentioning, but also based on additional blast search reports for which we used two comprehensive databases (euk_nt and tsa_nr).
Comment 6. Genome contamination: According to Supplementary File 5, sample 70 appears to exhibit two peaks in GC content. Does this indicate possible contamination? The authors should provide more regarding this observation and assess how it may affect the interpretation of the myxozoan genomes.
This was also raised in several comments by Referee 1 for which we have also offered an explanation and additional text. Please see our responses to Referee 1 comments 3, 4, 6 and 7.
Briefly, two GC content peaks were expected based on our findings using MethMod and weighted KDE to analyze sample 70. One peak (with greater GC content) represented CDSs that were likely methylated, and a second peak (lower GC content) which represented CDSs which were not. This is characteristic for invertebrate genome methylation rather than contamination. To further rule out contamination, we used exhaustive filtering plus BlobTools post assembly. Additionally, we performed 18S rRNA analysis by analysis of the entire SSU molecule for variable and hypervariable regions (which we now include in the revised manuscript as Supplementary File 5) and by performing a phylogenetic analysis that confirmed this sample represented a single distinct species of Myxozoa. This phylogeny is uploaded to the GigaDB for review by the referees and we have included a new Supplementary File 6 into the Results section together with supporting text in the Methods section, thus:
Phylogenetic analysis encompassing 678 Myxozoa 18S rRNA sequences, where 673 have been obtained from Silva database (ref) with the addition of 5 more extracted from our genomes. The analysis was based on multiple sequence alignment where all insert columns have been removed, including any consensus column for which fewer than 95% of the sequences that are nongaps and have a posterior probability of less than 0.95. This data preparatory process, which assumes all aligned nucleotides are homologous, ensures that the resulting phylogeny is based on robust data. The alignment and variable region masking have been performed using SSU-ALIGN (PMID: 11869452, and the phylogenomic inference including tree reconstruction has been accomplished using IQ-TREE (PMID: 32011700) installed on a Linux server. The built-in "ModelFinder" feature (PMID: 28481363) has been used to determine the best-fit substitution model for 18S DNA multiple sequence alignment input. For the visualization of the resulting unrooted tree, iTol v6 has been used (PMID: 38613393) has been utilized.
Additional Suggestions:
Comment i: Ensure consistent sample descriptions and avoid using sample IDs when describing species.
Consistent species descriptions have now been used throughout the manuscript, tables and figures.
Comment ii: Figure quality should be improved; the workflow should be clearly presented, and legends should be complete.
This has been corrected.

	Comment iii: The BUSCO figure appears to be incorrect, as sample 70 is indicated to have the most complete BUSCOs but with a short blue bar.<br>The referee is mistaken, there is no error in the BUSCO figure. BUSCO reports complete and single copy, complete and duplicated, fragmented and missing BUSCOs. These four categories are represented by different colours which are included in the legend. Confusion may relate to the first two categories that overlap on "complete" BUSCOs. Since these overlap, we ask the referee to look at where the green bar stops and not the blue bar. It should then be clear that sample 70 is indeed the most complete genome and, therefore, that the figure is correct. Other genomes that also exhibit duplicated complete BUSCOs display similar "anomaly" (Kudoa iwatai and Sphaeromyxa zaharoni), which is not an error but simply the fact that one needs to account for overlapping sets of BUSCOs. Extending the blue bar representing complete BUSCOs and then repeating this for the portion of complete BUSCOs that are also being duplicated would not be correct.<br>Comment iv: CDS stands for coding sequences, not coding regions.<br>This has been corrected throughout the text.
**Additional Information:**	
**Question**	**Response**
Are you submitting this manuscript to a special series or article collection?	No
**Experimental design and statistics**<br><br>Full details of the experimental design and statistical methods used should be given in the Methods section, as detailed in our Minimum Standards Reporting Checklist. Information essential to interpreting the data presented should be made available in the figure legends.<br><br>Have you included all the information requested in your manuscript?	Yes
**Resources**<br><br>A description of all resources used, including antibodies, cell lines, animals and software tools, with enough information to allow them to be uniquely identified, should be included in the Methods section. Authors are strongly encouraged to cite Research Resource Identifiers (RRIDs) for antibodies, model organisms and tools, where possible.<br><br>Have you included the information requested as detailed in our Minimum Standards Reporting Checklist?	Yes

**Availability of data and materials**<br><br>All datasets and code on which the conclusions of the paper rely must be either included in your submission or deposited in publicly available repositories (where available and ethically appropriate), referencing such data using a unique identifier in the references and in the "Availability of Data and Materials" section of your manuscript.<br><br>Have you have met the above requirement as detailed in our Minimum Standards Reporting Checklist?	Yes

Powered by Editorial Manager® and ProduXion Manager® from Aries Systems Corporation

Revised Manuscript
Click here to access/download;Manuscript;REVISION FINAL.docx

Long read metagenomic sequencing negates inferred loss of cytosine methylation in Myxosporea (Cnidaria: Myxozoa)

Running title: Gene body methylation in Myxozoa

Antonio Starcevic[1] [0000-0003-2386-2124], Rayline T. A. Figueredo[2] [0000-0003-4100-9039], Juliana Naldoni[3] [0000-0002-6764-448X], Lincoln L. Corrêa[4] [0000-0002-6453-4824], Beth Okamura[5] [0000-0001-7279-715X], Edson A. Adriano[6] [0000-0002-6903-9531], Paul F. Long[7,8*] [0000-0001-6698-4602]

[1]University of Zagreb Faculty of Food Technology and Biotechnology, Pierottijeva 6, HR-10000 Zagreb, Croatia. (Email: antonio.starcevic@gmail.com)
[2]Department of Animal Biology, Institute of Biology, University of Campinas, Campinas, 13083-970, SP, Brazil. (Email: rayline.figueredo@gmail.com)
[3]Department of Pathology, University of Cambridge, Tennis Court Road, Cambridge, CB2 1QP, UK (Email: jnaldoni@gmail.com)
[4]Institute of Water Sciences and Technology, Federal University of Western Pará (UFOPA), Santarém, 68040-255, PA, Brazil. (Email: lincorre@gmail.com)
[5]Life Sciences, Natural History Museum, Cromwell Road, London, I SW7 5BD, United Kingdom. (Email: b.okamura@nhm.ac.uk)
[6]Universidade Federal de São Paulo, Instituto de Ciências Ambientais, Químicas e Farmacêuticas, Diadema, 09972-270, SP, Brazil. (Email: adriano@unifesp.br)
[7]Institute of Pharmaceutical Science, King's College London, 150 Stamford Street, London, SE1 9NH, United Kingdom. (Email: paul.long@kcl.ac.uk)
[8]Faculdade de Ciências Farmacêuticas, Universidade de São Paulo, Av. Prof. Lineu Prestes, 580, B16, 05508-000 São Paulo, SP, Brazil.

*Corresponding author Email: paul.long@kcl.ac.uk

Key words: cnidarians, DNA methylation, long-read sequencing, bioinformatics

ABSTRACT

Oxford-Nanopore PromethION sequencing is a PCR-free method that retains epigenetic markers and provides direct quantitative information about DNA methylation. Using this long-read sequencing technology, we successfully assembled five myxozoan genomes free from discernible host DNA contamination, surpassing previous studies in both quality and completeness. Genome assembly revealed DNA methylation patterns within myxozoan genomes, particularly in GC-rich regions within gene bodies. The findings not only refute the notion of myxozoans lacking DNA methylation capability but also offer a new perspective on gene regulation in these parasites. The high-quality genome assemblies lay a solid foundation for future research on myxozoans, including new strategies to control these commercially significant fish pathogens.

INTRODUCTION

Epigenetic processes enable cells to control gene activity without altering DNA sequences [1]. Cytosine methylation is an epigenetic mechanism widely found in eukaryotes and involves DNA methyltransferases (DNMTs) that transfer a methyl group from S-adenosylmethionine to the C5 position of cytosine of genomic DNA [2]. Such cytosine methylations are subsequently recognised by methyl-CpG binding domain proteins (MeCP2 and MBD1-4), leading to transcriptional silencing and the subsequent generation of phenotypic variation [3-6]. Together, DNMTs and MBDs

when complexed with other proteins, comprise the core metazoan DNA methylation system found in both vertebrates and invertebrates [7]. The regulation of gene expression achieved by cytosine methylation is linked with many key processes in animals including gametogenesis, embryonic development, cellular differentiation, X-chromosome inactivation and transposon repression [8]. Most investigations of cytosine methylation have been conducted on free-living animals, but some have focused on endoparasites where they have been linked with persistence in hosts. For example, cytosine methylation in the cestode, *Taenia solium*, is associated with key parasitism-related genes (secretory proteins) leading to the suggestion that targeting DNA methylation processes may offer a therapeutic strategy [9].

Cytosine methylation contributes to key biological processes, and is viewed as highly conserved, occurring in viruses, prokaryotes and eukaryotes [5, 10, 11]. Nevertheless, cytosine methylation is absent in the nematode *Caenorhabditis elegans* [12]. There is also evidence for loss in all dipterans and some hymenopterans [13], and possibly in the helminth *Schistosoma mansoni* [14, 15]. Knowledge of distinct epigenetic processes will expand our understanding of gene regulation and provide more nuanced views on the relevance of model organisms, such as *C. elegans* and *Drosophila melanogaster*. Alternative mechanisms of DNA modification, such as adenine methylation [16], may act as a substitute in invertebrates (*C. elegans* [12], *D. melanogaster* [17]), in mammals (mouse embryonic stem cells [18]) and in plants (*Arabidopsis thaliana* [19]). However, it is not always clear what replacement mechanisms may be operating for organisms that appear to have lost cytosine methylation [11].

Another epigenetic mechanism widely found in eukaryotes, including parasites, involves modification of histone proteins [20]. Control of histone gene expression ensures that a fine balance is maintained between histone abundance, correct packaging of DNA into chromosomes, and regulation of DNA transcription [21]. Differential expression of histones has been linked with endoparasitism, for example, in eukaryotic microbial organisms such as *Plasmodium falciparum* [22], *Leishmania infantum* [23], *Trypanosoma cruzi* [24] and the myxozoan *Myxobolus bejeranoi* [25]. Transcriptomic analyses of *M. bejeranoi* infecting cultured tilapia hosts also provided evidence for regulation of gene silencing by miRNA, which is a widely recognised epigenetic mechanism associated with host-parasite interactions [26, 27].

Histone deacetylation and CpG methylation are interconnected in several ways, including acting in concert to effect one of the more important epigenetic processes – gene silencing [28]. When histone deacetylases remove acetyl groups from histone proteins, it leads to a more condensed chromatin structure, making DNA less accessible for transcription factors and RNA polymerase. Methylation of CpG islands in promoter regions or within genes themselves (a process called gene body methylation) can repress gene transcription. Moreover, methylated CpG sites can recruit proteins like Methyl-CpG Binding Proteins [29]. These proteins can, in turn, recruit histone deacetylases to further compact chromatin and inhibit gene expression. These collective processes can create a reinforcing loop with histone deacetylation and DNA methylation working together to maintain gene silencing. Certain multiprotein complexes, such as the MeCP1 complex, contain both DNA methyltransferases (which add methyl groups to CpG sites) and histone deacetylases [30], which indicates a direct physical interaction that promotes these two epigenetic mechanisms [31]. The

exact outcome of this interaction depends on the specific context, genomic location and interacting proteins.

Recently Kyger et al. [10] proposed that most myxozoans lack cytosine methylation based on analyses of two distant species of myxosporeans – the clade comprising the vast majority of myxozoan species [32, 33]. They first searched for methylation-associated proteins in transcriptomic and genomic data from 29 cnidarians, including eight myxosporean species. They then conducted whole genome bisulphate sequencing (WGBS) of two distantly related myxosporeans to determine which cytosine residues were methylated. Neither approach provided evidence for cytosine methylation in myxosporeans. However, they detected methyl-associated proteins in many free-living cnidarians and the cytosine methylation-associated protein, MDB1/2/3, was identified in a limited EST library of the malacosporean myxozoan, *Buddenbrockia plumatellae*. This collective evidence led Kyger et al. [10] to conclude that myxosporeans are one of the few confirmed instances of animals that have secondarily lost cytosine methylation capability. They proposed that cytosine methylation has been retained, or is in the process of being lost, in the taxon-poor sister clade – the Malacosporea.

Although the WGBS method used by Kyger et al. [10] is a powerful approach, various associated processes can lead to false negative results. If the bisulfite conversion reaction is incomplete, some unmethylated cytosines may not be converted to uracil, leading to false negative results [34]. DNA fragmentation or degradation can also result in the loss of methylated cytosines. Furthermore, PCR amplification can introduce biases in the representation of methylated and unmethylated DNA sequences. Critically, the presence of host DNA greatly challenges characterising endoparasites using untargeted Next Generation Sequencing (NGS) approaches. In

particular, untargeted DNA sequencing of all genomes within a sample produces highly complex datasets with millions of short reads representing different genome fragments [35]. In this genomic mixture, host DNA may potentially overwhelm endoparasite signals during assembly. Thus, if only a limited fraction of the DNA derives from endoparasites, assemblies based on multiple samples and/or host DNA sequence filtering may be required to obtain reasonable coverage of endoparasite genomes. Finally, DNA methylation patterns can also be variable between cell types (i.e., host *versus* endosymbiont) or different endoparasite unicellular stages (i.e., plasmodia vs spores in the case of myxosporeans), potentially leading to false negative results if the methylated regions are not well represented in the sample [36]. To address these issues, it is important to sequence multiple independent samples or to use alternative sequencing methods.

Myxozoa comprise a bizarre radiation of extremely morphologically simplified parasitic cnidarians whose extensive molecular divergence long precluded their phylogenetic placement [37, 38]. However, it is now clear that they evolved within Cnidaria [39-41] and comprise some 20% of all described cnidarian species in the present day [33]. Furthermore, the speciose and diverse myxosporeans are highly derived and demonstrate a unique convergence to eukaryotic microparasitic lifestyles by developing exclusively as plasmodia and various invasive unicellular stages [37, 42]. This evolutionary trajectory may ultimately have been facilitated by the diploblast nature of cnidarians [43]. The discovery of vermiform plasmodia in gall bladders of Amazonian fishes, in turn, demonstrates convergence to metazoan worm-like forms achieved at the unicellular level by myxosporeans [44]. It is of considerable interest, therefore, to confirm whether proposed loss of cytosine methylation may be associated with the rapid evolution and the unique morphological trajectories involving

miniaturisation displayed by myxozoans. However, as outlined above, further confirmation of this loss is required in view of potential complications associated with bisulfite sequencing using the small read-length generating Illumina HiSeq platform adopted by Kyger et al. [10]. Oxford-Nanopore PromethION sequencing (ONT) is a PCR-free method that retains epigenetic markers and provides direct quantitative information about DNA methylation [45]. The aim of this study was to use ONT as an alternative method to ascertain loss of cytosine methylation in myxosporeans and to generate the first long sequence reads for myxozoan genomes.

METHODS

Fish sampling

Sampling and access to genetic heritage were authorized by the Brazilian Ministry of the Environment (SISBIO authorization #67616-2 and SisGen #A656D). The study was approved by the Ethics Committee on Animal Use of the Federal University of São Paulo-UNIFESP (CEUA #6549290920). Fish were caught during January 2022 using gill nets in waters where the Tapajós and Amazon Rivers merge, close to the city of Santarém, Pará State, Brazil (2°23′49.79″S 54°43′53.33″W). The fish were transported live to a makeshift field laboratory, close to the sampling sites, and euthanised prior to dissection. Our aim was to sample opportunistically for myxozoans in Amazonian fish gall bladders, which have been commonly reported to be infected by these cnidarian parasites (44). After the dissection of each fish the gall bladder was removed, and a sterile scalpel blade was used to open the gall bladder pouring the bile directly into a sterile 1.5 mL Eppendorf tube. With a sterile micro-pipette, a drop of the bile fluid was immediately placed on a microscope slide and examined using a

light microscope (Carl Zeiss model Primo Star) at 20x or 40x to ascertain infection. When infection was confirmed, the bile fluid content of the Eppendorf tube was immediately preserved in DNA/RNA Shield reagent (Zymo Research Corp., Irvine, CA, USA) to give 50 % (v/v) in a final sample volume of 900 µL. Pictures of parasites in bile were obtained using a digital camera (Sony CyberShot) coupled to the light microscope objective. Observations of plasmodia and, especially, of diagnostic myxospores with polar capsules enabled microscopic identification of myxozoan infections.

DNA isolation and sequencing

Metagenomic DNA was extracted from each sample using the Quick-DNA Fecal/Soil Microbe DNA Miniprep Kit (Zymo Research) in accordance with the manufacturer's instructions. The entire sample was added to a ZR BashingBead™ Lysis Tube and the first step of the extraction proceeded by bead bashing (Vortex-Genie 2, USA Scientific, Inc., Ocala, FL, USA) at 3,200 rpm for 2 minutes, followed by a rest on ice for 1 minute, repeating these steps 3 times. The concentration of DNA was measured using a NanoDrop 2000 spectrophotometer (Thermo Scientific, Wilmington, DE, USA). Metagenomic libraries were generated using ~200 ng DNA, and sequenced at the Novogene Sequencing Centre (Tianjin, China). Crucially the standard workflow deviated from the sequencing centre’s proprietary methodology in three respects. Firstly, DNA was not fragmented or size selected. Secondly, the 1D libraries were not purified following end-polishing, nick repair and ligation of sequencing adaptors. Thirdly, the libraries were pooled and sequenced, irrespective of the final library concentration, using an entire ONT sequencing cell per sample. The original nanopore signal was recorded in Fast5 format and base calling was achieved using Guppy v5.1.13 software to produce FASTQ format. Quality control of the raw reads was

conducted using NanoPlot software to remove adapter contamination and low-quality reads. Only reads with Q7 > 7 were used in subsequent assemblies. An overview of the entire ONT data analysis pipeline is given in Figure 1 and is next described in detail below.

Removal of fish host contamination and assembly of Myxozoa reads.

The first step to remove fish host DNA from each metagenomic DNA sample was to map reads onto a set of 21 complete reference genomes representing fish from the order Characiformes and family Sciaenidae (Supplementary File 1). Minimap2 [46] was used since this software allows mapping of long and noisy DNA reads to reference genomes with the “map-ont” option. All the reads that mapped to the fish reference genomes were removed as contaminants, leaving datasets comprised of Myxozoa-enriched reads. After this first round of read filtering, the remaining reads having average quality score > Q7 and with lengths > 3000 bp were used as inputs for Flye [47]. Flye is a *de novo* assembler for single genome and metagenome sequencing reads, such as those produced by the ONT sequencing [47]. It is important to note that Flye works using uncorrected reads as inputs and produces error-corrected contigs as outputs without the need to specify the expected genome size, which is very important since the expected genome sizes for Myxozoa are largely an unknown variable. The quality of the assembled contigs was further enhanced using Medaka (https://github.com/nanoporetech/medaka), which has been specifically trained to correct draft sequence outputs from the Flye assembler in a process called polishing, that pushes the error correction close to that expected from an assembly if it were constructed using Illumina-generated reads. Custom code was written to improve parallelism for this Medaka consensus generating step.

To eliminate any remaining host and other contaminating DNA that might have evaded the first stage minimap2-based filtering process (and thus might subsequently become erroneously assembled into chimeric contigs), a second more stringent filtering step was introduced that used BlobToolKit v4.2.0 [48]. This is a tool designed to aid genome assembly quality control, contaminant detection and filtering. This second stage filtering process involved: a) pairwise BLAST [49] comparisons between the Medaka polished assembled contigs for each Myxozoa sample against the entire GenBank Eukaryota nt (nt_euk) database, and; b) Diamond [50] BLASTx comparisons against the protein portion of the Transcriptome Shotgun Assembly Sequence Database (tsa_nr). The hits from these two BLAST analyses provided inputs which the BlobToolKit used, together with read coverage information, to obtain taxonomic assignments for the assembled DNA. Any contigs that provided majority of hits to host related taxa (Actinopterygii) were removed. The outputs, therefore, were Medaka-polished contigs that represented Myxozoa genomes clean of any detectable fish host DNA.

Lastly, the genome size for each of the samples was predicted by comparing each assembly total size (in bp) with the genome size estimated by K-mer count analysis using error corrected ONT reads. This approach was taken because Myxozoa are highly variable in terms of genome size and no true reference genomes exist. To estimate the genome sizes, all the Medaka polished contigs and all the error corrected long-reads were separately used for 21-mer and 25-mer frequency analyses. Jellyfish v2.1.3 [51] was used to count K-mer frequency, and a histogram of K-mer distributions was generated. Jellyfish could be used on ONT read data because read trimming and error correction steps were completed manually. Peak coverage was taken to be the average K-mer coverage, and genome size was estimated by the formula: G_{size} = K-

$mer_{count}/Peak_{position}$, where the $K\text{-}mer_{count}$ and $Peak_{position}$ are the total number and the average depth of 21-mer and 25-mer combinations, respectively. ONT read error correction was performed using Canu correction and trimming modules [52].

Assigning taxonomic labels to reads

To confirm Myxozoa identification based upon microscopic morphology and to check that the final datasets were free from host and other contaminating DNA, a search for both 16S rDNA and 18S rDNA taxonomic markers in raw and corrected reads was undertaken using the SILVA Release 138.1 non-redundant Small Subunit rRNA Database (https://www.arb-silva.de/ [53]). Hits to the dataset were assigned taxonomic labels using the all-species taxonomic framework function [54]. To assign taxonomic labels at a species level, a manual examination of alignment hits between the query reads and the SILVA database was performed to extract sequences that corresponded to 18S rDNA hypervariable regions. These regions were analysed by BLASTn alignment against unpublished Myxozoa 18S rDNA sequences generated by our group, and also against the entire nr nucleotide database at NCBI (https://blast.ncbi.nlm.nih.gov/Blast.cgi) to further confirm removal of contaminating sequences. Phylogenetic analysis was conducted on 678 Myxozoa 18S rRNA sequences, with 673 obtained from the Silva database [53] and 5 extracted from our genomes. The analysis was based on multiple sequence alignment where all insert columns have been removed, including any consensus column for which fewer than 95% of the sequences that are nongaps and have a posterior probability of less than 0.95. This data preparatory process, which assumes all aligned nucleotides are homologous, ensures that the resulting phylogeny is based on robust data. The alignment and variable region masking were performed using SSU-ALIGN [55], and the phylogenomic inference, including tree reconstruction, was accomplished using

IQ-TREE [56] installed on a Linux server. The built-in "ModelFinder" feature [57] has been used to determine the best-fit substitution model for 18S DNA multiple sequence alignment input. For the visualization of the resulting unrooted tree, iTol v6 was used [58].

Assessment of genome assembly completeness and quality

The completeness and quality of each individual genome assembly was assessed separately and then compared against all 8 Myxozoa genomes currently available (Supplementary File 2) using QUAST [59] and BUSCO analyses [60]. The QUAST analyses provided technical metrics with which to compare the relative completeness of genomes, for example, numbers and sizes of contigs, G+C content, numbers of base mismatches, and overall quality in terms of N50, N90, L50 and L90 values. Genome quality was also assessed by measuring anticipated gene content using BUSCO analysis with a 255 single-copy ortholog dataset (Eukaryota Odb10) that is considered representative of genes that encode core biological functions in eukaryotes [61, 62]. To be included in the BUSCO set, genes had to be present as single copy orthologs in at least 90% of the species within a major branch of the species phylogeny. Additionally, to ensure a diverse distribution across various taxa, the BUSCO dataset does not contain a single sub-clade where all the genes were missing. The BUSCO analyses were implemented using a docker container built from hub.docker.com/r/vera/busco. BUSCO analyses using the same Eukaryota Odb10 dataset provided standardized metrics that allowed comparative genome quality to be determined between the genomes assembled herein, and those Myxozoa genomes already available in public databases.

Methylation calling

DNA methylation signals were present in the fast5 files, which originated directly from the ONT sequencing cell (PromethION FLO-PRO002 with SQK-LSK110 sequencing kit) and which were also used for base-calling. We adopted Nanopolish v.0.14.0 [45], an analytic tool that utilises a hidden Markov model to distinguish 5-mC from unmethylated cytosine directly from fast5 files, for two reasons. First, it uses an agnostic model approach to detect DNA methylation that does not require training data, which is a constraint when dealing with relatively unknown genomes. Second, the method has been shown to either outperform or provide equivalent results for methylation calling offered by other approaches [63] that are highly dependent on the training data. Since Nanopolish needs to access the signal-level data measured by the ONT sequencer, index files were created that linked long-read identifiers with the corresponding signal-level data in the fast5 files. Next, the base-called reads were aligned to each assembled genome contig, using the ONT specific “map-ont” option of minimap2. Nanopolish was then used to detect methylated bases (5-methylcytosine in CpG context) using the internal “call-methylation” function. The original Nanopolish output file contained much information including, identifiers, the position of the CG dinucleotide on genome contigs, and the log-likelihood ratio calculated by the model. A positive value in the log_lik_ratio column of the output files indicated support for methylation. By using a helper script called “calculate_methylation_frequency.py” accessible within the Nanopolish installation files, the methylation frequency for all detected CpG sites on each assembly contig could be calculated. These methylation frequency values range from 0 to 1, with zero indicating no support for methylation whatsoever, and 1 indicating that all the sequence data covering a particular CpG site had the methylation signal. Each value placed between 0 and 1 boundaries represents

the ratio of mapped sequence data giving a positive methylation signal for a particular CpG site.

Gene finding, annotation and genome comparisons

Because DNA material was isolated from endoparasites living within their hosts, MetaEuk v.6.a5d39d9 was used for gene discovery and annotation [64]. This toolset is designed specifically to handle large-scale datasets reflecting eukaryotic metagenomic contigs. It also integrates the rapid and sensitive homology search functionalities offered by MMseqs2 with a dynamic programming approach tailored for the retrieval of optimal exon sets [65]. For both gene annotation and taxonomic assignment, the latest UniProtKB database was used. The entire set of UniProtKB protein sequences was downloaded locally in FASTA format and converted to become our own customized baseline annotation database using the MMseqs2 “mmseqs” command. This procedure enabled us to augment the sequence database with additional taxonomic information using the sequence accession descriptions, thus linking our sequence data with taxonomic identity using an appropriate mapping file. The “easy-predict” MetaEuk workflow was used on all assembled genome contigs and scaffolds to predict protein coding sequences and respective proteins in a non-redundant manner. To complement the customized UniProtKB reference database, the entire current release of PANTHER HMM library (v.18) was also downloaded and installed locally for functional classification of proteins [66]. After obtaining gene predictions, the “taxtocontig” workflow in MetaEuk was used to assign taxonomic labels to the predicted proteins. The parameter “--majority” was used with this workflow, so that at least 50% of labelled predictions were used for positive taxonomic assignment. To integrate this workflow into our Myxozoa genomic pipeline (Figure 1), several Python scripts were written for more efficient parallelization of search task, for

PANTHER search output parsing, and for further downstream analysis. For example, the PANTHER HMM scoring tool was used to screen a Hidden Markov Models (HMM) library specifically for all protein families related to the nucleus-based GO biological processes of base-excision repair (BER), DNA methylation (DNMT) and DNA demethylation (TET).

Word Cloud analysis of Gene Ontology terms linked to genes was used as a visualization strategy and aimed to enhance the interpretation of the vast and complex information the genome annotations yielded. Keywords and key phrases were extracted from the textual descriptions of gene functions using a state-of-the-art keyword extraction tool KeyBERT (https://github.com/MaartenGr/KeyBERT). The extracted terms were next visualized using WordCloud, which provides a powerful tool for representing the relative importance of terms through graphical representation (http://amueller.github.io/word_cloud/).

Determining cytosine methylation at CpG sites within and outside protein coding sequences (CDSs)

The "taxtocontig" and "--lca-mode" function of MetaEuk was used to predict the taxonomy of CDS and non-CDS sequences. The additional parameter "--majority" was also used with this workflow. All predictions were based on candidate sequence alignment to the UniProtKB database used for genome annotation. Alongside this pipeline, a set of Python scripts and programs were written utilizing BioPython (https://biopython.org/) modules to parse the gene prediction output files and to link these predictions to other layers of information. The average G+C content of CDS and non-CDS sequences was then calculated based on predicted sequence boundaries within each genome assembly. Knowing the G+C content, the Observed–Expected

CpG ratio (CpG O/E) for each CDS and non-CDS sequence in each genome could be calculated using the following equation:

$$CpG\ O/E\ = \frac{CG\ x\ l}{C\ x\ G}$$

where "l" is the sequence length, "C" denotes the number of cytosine residues and "G" the number of guanine residues within a sequence. A Python implementation of a Gaussian Mixture Model (GMM) called "MethMod" (https://github.com/JanLeipzig/MethMod/) was used to predict the presence of DNA methylation based on observed differences between CpG rates in CDSs extracted from genome annotations [64]. The GMM had two components and calculated the Akaike information criterion (AIC) alongside the statistical mean of each component. The distance between the statistical mean of each component and the relative amount of data points in each component were also calculated (i.e., the percentage). To characterise the distributions of each Myxozoa genome CDS CpG O/E values, the distribution means, sample standard deviations, and the skewness were also calculated. To report only the reliable "MethMod" calculations, shuffled data were used as a control. The most informative "MethMod" value calculated is the distance between the component means, which serves as an indicator for DNA methylation. If the distance is greater or equal to 0.25, it can be assumed that DNA methylation is present, otherwise it is more likely that DNA methylation is absent [67].

Statistics used for validation

To determine if the number of CpG sites within a CDS was correlated to the length of the CDS (in bp), the "Matplotlib" and "NumPy" modules in BioPython were used to calculate a Pearson's correlation coefficient. The lengths and the observed number of CpGs for each CDS in the three genome assemblies were plotted, and a linear least-

squares regression was used to model the relationship between the number of CpG sites and CDS length. A Kernel Density Estimation (KDE) was used to further validate the “MethMod” results and to justify the application of this method on our Myxozoa assemblies. It is important to notice that the GMM used in MethMod is a parametric probability density function represented as a weighted sum of Gaussian component densities. As such, it has to make an underlying assumption on the number of components. In the case of DNA methylation, two components are a natural choice accounting for methylated and unmethylated genes. However, in view of the rapid evolution that characterises myxozoans, the non-uniform myxozoan genome assemblies, and the Kyger et al [10] results indicating absence of methylation; an additional non-parametric method (KDE) was used to validate the parametric GMM test results.

KDE applies kernel smoothing for probability density estimation and represents a non-parametric method often used to estimate the probability density function of a random variable based on kernels as weights. Testing different kernels for KDE is important because the choice of kernel can have a significant impact on the estimated probability density function. Three were selected, and these were the “Epanechnikov”, “tophat” and “Gaussian” kernels, with Gaussian providing the closest estimate. A code to generate KDE was written using “NumPy”, “Matplotlib”, “SciPy” and “scikit-learn” modules of BioPython. The code resulted in bimodal distributions based upon the kernel density estimates, fitted to the ONT Nanopore predicted myxozoan CDS CpG O/E data. To determine the exact location of each methylated base (i.e., 5-methylcytosine in a CpG context) for each assembled Myxozoa genome, the fast5 datasets were processed using the same Nanopolish, Guppy, minimap2 pipelines described previously. To demonstrate the inverse correlation between calculated CDS

CpG O/E and experimentally recorded CpG methylation frequencies within the respective CDSs gene bodies, a scatter plot was drawn. This scatter plot included a group of 1000 CDSs characterised by highest calculated CpG O/E ratios, and another group consisting of the same number of CDSs with the lowest CpG O/E ratios in the dataset. On the Y-axis for each CDS group, a cumulative methylation frequency (expressed as an average) was calculated by summing all respective CpG site methylation frequencies and dividing this sum by the number of CpG sites for each of the selected CDSs. To smooth the methylation frequency plot and to make a more informative figure, a Savitzky-Golay filter was implemented from the “SciPy” module of BioPython. An elbow method was additionally employed to independently assess the optimal number of clusters e.g., distributions (e.g. number of components) according to CDS CpG O/E values. To go even further and validate these expectations using experimental data obtained by ONT sequencing, the KDE analysis was repeated and ONT Nanopolish derived methylation frequencies were introduced which were calculated for each CpG site within CDSs in its full range (floating point value from 0 - 1 range) as function weights. Thus, in this weighted KDE approach an entire methylation frequency floating point scale from 0 to 1 was utilized allowing us to pinpoint CpG O/E values that are best at separating methylated vs non-methylated CDSs. Since CpG O/E values can be calculated solely based on genomic sequences, this is a very useful method for proving the existence of methylation in genes and to provide the accurate boundaries separating methylated from non-methylated genes within genomes. All figures were drawn using “matplotlib” within the Anaconda environment (https://anaconda.org/conda-forge/matplotlib) and using JupyterLab (https://jupyter.org/).

RESULTS

Identification of Myxozoa

We detected myxozoan infections in 102 of 189 fish gall bladders (54%). The DNA of myxozoans collected from the bile of five fish specimens was isolated in sufficient concentration and purity required for ONT sequencing. Just over 9 terabytes of sequencing data were generated. To assign taxonomic labels to the Myxozoa, both raw and corrected reads were used as inputs for identification of 16S and 18S rDNA sequences by comparison to SILVA Release 138.1. Table 1 summarises the material studied (FASTA sequences are provided in Supplementary File 3). Light microscopy at low magnification without oil (typically x 20 or x40) was also used to identify myxozoan infections and to tentatively identify the myxozoans to genus level based on morphology (Table 1). Photographs of the Myxozoa and host fish are provided in Supplementary File 4 for four of the five bile samples listed in Table 1. Closer inspection of the *Ceratomyxa* spp. isolated from fish samples 57 and 58 (*Plagioscion squamosissimus*) revealed cell morphologies reflecting different stages of development (Supplementary File 4, Figure 1). These ranged from immature to mature plasmodia, the latter containing numerous spores. Advanced sporogenesis stages were predominant. The identification of plasmodia and myxospores was associated with hits to 18S sequences of myxozoans in the genus *Ceratomyxa* in the SILVA database. The inferred identification of Myxozoa found in the bile from fish sample 70 (*Curimata inornata*) was inconsistent between the molecular and microscopic data. Molecular data indicated a closest database match to *Ceratomyxa.* However, the morphologies of parasite stages in the bile (many immature plasmodia, some mature plasmodia with myxospores and some free myxospores) were clearly those of *Ellipsomyxa* (Supplementary File 4 Figure 2). Two morphological types of Myxozoa were observed in the bile collected from sample 115 (*Hemiodus unimaculatus*). These

were mainly *Ceratomyxa* plasmodia at different levels of sporogenesis and free myxospores, but also some stages consistent with the morphology of immature *Ellipsomyxa* plasmodia without myxospores (Supplementary File 4 Figure 3). It would appear that *Ellipsomyxa* DNA was sequenced from sample 115 as only sequences homologous to *Ellipsomyxa* were matched in the SILVA database. Sequences homologous to infection by *Ellipsomyxa* and *Myxidium* were retrieved in the SILVA database for sample 108 (*Rhaphiodon vulpinus*, a photo of the fish is given in Supplementary File 4 Figure 4). Photographic evidence was not obtained for Myxozoa in this sample, but *Ceratomyxa* sp. was identified by microscopic examination and recorded in the field notebook.

To confirm species identification, the identified 18S FASTA sequences (Supplementary File 3) were analysed manually for hypervariable regions (Supplementary File 5). Subsequent phylogenetic analysis revealed no overlap of 18S sequences with those of existing myxozoan sequences deposited in the SILVA database (Supplementary File 6A provides a phylogenetic tree where our samples are labelled in red. The phylogeny is also represented in Newick format in Supplementary File 6B). Very recently published Canu built-in methods that handle the typically high read error rates linked with nanopore sequencing were employed for error correction and read trimming to provide more reliable 18S rRNA gene analysis [68]. However, no convincing homology alignments could be made with known myxozoan species. In addition, a pairwise assessment of genome synteny was performed using ONT-generated polished assemblies to determine if any of the samples shared identity. Using standalone D-Genies [69] to display the synteny outputs, it was possible to globally align sample 57 to 58 (Figure 2A), and sample 108 to 115 (Figure 2B). Such synteny further strengthened the previous 18S rDNA based taxonomic assignments

(Table 1) which, together with the annotation results reported below, strongly support identity of myxozoans in samples 57 and 58. These were likely the same *Ceratomyxa* species isolated from different specimens of the same fish species. The synteny result also supported the identity of myxozoans in samples 108 and 115, these being *Ellipsomyxa* isolated from two different fish species. However, later comparisons between the functional annotations of genes would suggest a clear taxonomic difference between samples 108 and 115. In the absence of photographic evidence, sample 108 was considered a myxosporean species and is referred to as Myxosporea sp.

<u>Myxozoa assembly comparisons</u>

Ceratomyxa sp. sample 57 and *Ceratomyxa* sp. sample 58 both contained large numbers of myxospores (Supplementary File 4 Figure 1), but only provided partial genome assemblies and were discounted from further genomic comparisons. ONT read assembly provided three high quality draft Myxozoa genomes: for *Ellipsomyxa* sp. sample 70, Myxosporea sp. sample 108 and *Ellipsomyxa* sp. sample 115. Overall, this produced satisfactory results despite the initial imbalance of reads ranging from 10 to 1 in favour of host DNA in *Ellipsomyxa* sp. sample 115 and up to 4 to 1 in favour of host DNA in *Ceratomyxa* sp. sample 58. The only sample that had relatively more parasite reads compared to the host was *Ellipsomyxa* sp. sample 70, which revealed a 2 to 1 ratio in favour of parasite DNA reads. These calculations were based on ratios between excluded reads (i.e., ones that mapped onto reference fish genomes) and reads that mapped onto contamination pruned assemblies. The eight Myxozoa genome assemblies currently available in public databases were all assembled from reads generated using various Illumina sequencing platforms (Supplementary File 2). QUAST analysis clearly demonstrated that the three genomes generated from

samples *Ellipsomyxa* sp. sample 70, Myxosporea sp. sample 108 and *Ellipsomyxa* sp. sample 115 had superior overall quality to the eight published genomes in terms of best N50, N90, L50 and L90 values, and were also completely free of mismatches (Supplementary File 7). The estimated genome sizes were in the 20 - 30 Mbp range. These estimated genome sizes were further confirmed by Jellyfish K-mer analysis which revealed total genome lengths for *Ellipsomyxa* sp. sample 70, Myxosporea sp. sample 108, and sample 115 as 24.26, 26.97 and 30.22 Mbp, respectively. Our estimated total genome sizes are comparable to the 31.2 Mbp genome size estimated for *Kudoa iwatai*, but far smaller than the predicted genome sizes for the other seven previously sequenced myxozoans, which range from 61.44 – 234.48 Mbp (Supplementary File 7).

The QUAST comparison only provided a technical assessment of the genome assemblies, with no regards to functional aspects or genome completeness. To compare the ONT assembly more thoroughly to the previously generated Myxozoa assemblies that were all generated by Illumina platforms, a BUSCO based assessment was performed (Figure 3). This assessment included the entire dataset of 11 Myxozoa genome assemblies. There were 163 complete and unique BUSCOs identified from a total of 255 eukaryotic genes that comprised the BUSCO Eukaryota Odb10 dataset (63.9%). A reduced set of BUSCO genes is expected given the observed trend in parasite genome reduction. However, there were only 6 (2.4%) BUSCOs shared by all the genomes assessed. The highest quality genome assembly belonged to *Ellipsomyxa* sp. sample 70, which scored a total of 123 complete and duplicated BUSCOs. This represented 75.5% of the overall 163 BUSCOs identified. All other assemblies shared a majority of identified complete BUSCOs with this assembled sample 70 genome (ranging from a minimum of 68% shared with

Myxobolus honghuensis to 90% shared with Myxosporea sp. sample 108 and with *Enteromyxium leei*). It is therefore reasonable to consider *Ellipsomyxa* sp. sample 70 as the best quality myxozoan genome assembly reported to date. The overall low number of shared BUSCOs between *Ellipsomyxa* sp. sample 70 and the other assembled genomes could be attributed to incompleteness, excessive fragmentation, contamination (including co-infections of myxozoan species, a scenario that may partly explain our identification incongruities and could contribute to variation in qualities of assemblies), or a combination of these factors (Supplementary File 7). Even though a high-level eukaryote BUSCO set was used, a notable proportion of BUSCOs detected were duplicates. These ranged from 0% to 72% of all identified complete BUSCOs in the assembled genomes (Figure 3). This result is striking given the ubiquitous underlying basis of the BUSCO set (Eukaryota Odb10) and that the genomes are reduced in size (Supplementary File 7). The unusually low level of BUSCOs shared amongst these myxozoan taxa, also suggests that a small consensus of core proteins may be essential for endoparasites with reduced genomes. This was explored further when the genomes were annotated and is described below.

Gene annotation and genome analysis

Comparisons of the gene annotations for ONT sequenced *Ellipsomyxa* sp. sample 70, Myxosporea sp. sample 108 and *Ellipsomyxa* sp. sample 115 are shown in Figure 4. The Euler-Venn diagrams reveal that most genes are common to all three genomes, irrespective of the level of GO annotation used (molecular function, biological process, or cellular component). Although the level of unique functions, processes or components shared between Myxosporea sp. sample 108 and *Ellipsomyxa* sp. sample 115 was rather small (between 4 and 10%), the genome annotations were not completely identical. This further supported variation in 18S rDNA sequences, genome

synteny, microscopic morphology and BUSCO analyses that suggested Myxosporea sp. sample 108 and *Ellipsomyxa* sp. sample 115 were different taxa. A genome analysis revealed a core gene repertoire that contained 39,140 gene variants (76.83% of all genes in the top 20 GO terms) falling within molecular function GO process, 8,339 genes annotated with a biological function (43.80% of all genes in the top 20 GO terms) and 23,186 genes assigned to a cellular component (92.60% of all genes in the top 20 GO terms). Given the large number of genes that share predicted coding functions amongst all 11 myxozoan genomes, we used Word Cloud analysis to decipher what protein functions were overrepresented within the core gene repertoire (Supplementary File 8). Key words and key terms could then be more easily distinguished from the word clouds (see Methods), and these are shown in Table 2. In summary, when *Ellipsomyxa* sp. sample 70 was used as a reference genome, both BUSCO (Figure 3) and genome analysis (Figure 5, Table 2) concurred that the same 20 most abundant GO terms were common to *Ellipsomyxa* sp. sample 70 and all the other 10 assemblies (i.e., samples Myxosporea sp. sample 108 and *Ellipsomyxa* sp. sample 115, and the eight previously published myxozoan genomes). These data support that myxozoans possess a conserved core function gene repertoire, perhaps essential for endoparasites with reduced genomes which was not immediately evident when relying solely on gene homology.

<u>ONT nanopore methylation calling</u>

The sequencing reads for *Ceratomyxa* spp. isolated from fish samples 57 and 58 could only be assembled as partial genomes. However, these genomes displayed close to total synteny (Figure 2a). This, along with similar gene annotation results, indicated the same *Ceratomyxa* species was present in the bile of individuals of the same fish species that were caught at the same time. These partial genomes can thus be

considered as independent biological replicates with which the Nanopolish DNA methylation calling can be assessed. The sample methylation cross-comparison (bi-directional methylation calling) of *Ceratomyxa* sp. sample 57 and *Ceratomyxa* sp. sample 58 in both directions validated the ONT methylation calling results obtained using Nanopolish for each sample independently. These analyses also provided further support for the same species of *Ceratomyxa* in samples 57 and 58. Figure 6A is a heat map for the results of such a cross-comparison analysis using *Ceratomyxa* sp. sample 57 signal-level data mapped against *Ceratomyxa* sp. sample 58 genome assembly data as the biological replicate. Figure 6B is the reverse result whereby sample *Ceratomyxa* sp. sample 58 signal-level data were mapped against *Ceratomyxa* sp. sample 57 genome assembly data as the biological replicate. The heatmaps obtained by grouping comparable methylation frequencies across all CpG sites in the two sample assemblies indicated two things. First, the correlation was very strong (Pearson's correlation coefficient as a measure of the linear relationship between two variables = 0.764 in both sample comparisons), validating the ONT methylation calling without the necessity of employing bisulfite sequencing. Second, most of the recorded methylation frequencies fell in the range between 0.8 and 1. This provides strong evidence that most of the assembled genome CpG sites were methylated and thus, that most of the genes in these genomes were likely to be transcriptionally silenced at the time of sampling.

The cross-comparison additionally provided a procedure to determine the quality and accuracy of the sequence assemblies and to identify conserved regions or orthologous genes between the samples. The methylation frequencies and associated gene counts for assemblies (genome size estimates) of *Ceratomyxa* sp. sample 57, *Ceratomyxa* sp. sample 58, *Ellipsomyxa* sp. sample 70, Myxosporea sp. sample 108 and

Ellipsomyxa sp. sample 115 are displayed as a bar chart (Figure 7). The methylation frequencies for assemblies obtained from *Ellipsomyxa* sp. sample 70, Myxosporea sp. sample 108 and *Ellipsomyxa* sp. sample 115 are in striking contrast to those from samples *Ceratomyxa* sp. sample 57 and *Ceratomyxa* sp. sample 58. This surprising result indicates that most of the genes in the assemblies deriving from *Ellipsomyxa* sp. sample 70, Myxosporea sp. sample 108 and *Ellipsomyxa* sp. sample 115 were likely not methylated. Figure 7 also illustrates that the *Ellipsomyxa* sp. sample 70 assembly is arguably the cleanest and most accurate myxozoan genome yet described. This is supported by the genome assembly analyses (the QUAST technical assessment of the genome assemblies [Supplementary File 7] and BUSCO evaluation of genome completeness [Figure 3]).

Distribution of DNA cytosine methylation proteins

To rigorously assess the feasibility of CpG methylation capabilities of myxozoans, all eight Myxozoa genomes currently available and the additional 5 ONT based assemblies produced herein were all annotated using the same bioinformatics pipeline (Figure 1). Table 3 summarizes the presence of the most relevant mechanisms supporting molecular methylation in these various myxozoan genomes identified by the results of this uniform annotation procedure. These include DNA methyltransferase (DNMT), ten-eleven translocation methylcytosine dioxygenase (TET) and base excision repair (BER) enzymes. Five myxozoan genomes encoded all three key methylation components necessary for successful CpG methylation/demethylation and subsequent base excision repair (*M. honghuensis*, *Ceratonova shasta*, *Ellipsomyxa* sp. sample 70, *Ellipsomyxa* sp. sample 115 and *Henneguya salminicola*). The remaining seven genomes were missing either TET or DNMT components. BER was the dominant methylation enzyme in all the genomes, and more than one copy

was present in all genomes analysed. BER was absent in the partial genome assemblies of *Ceratomyxa* sp. sample 57 and *Ceratomyxa* sp. sample 58, both of which were also characterised by missing genetic information. The most important enzyme class for inferring CpG methylation is DNMT. DNMT was the second most abundant methylation mechanism, being present in 8 genomes, including *Ceratomyxa* sp. sample 57 which provided strong evidence for extensive CpG methylation (Figure 7). TET was the third most abundant methylation enzyme and had protein family representatives in over 50% of analysed genomes.

Distribution of CpG sites within and outside CDSs (protein coding sequences)

In general, there is a positive correlation between gene GC content and the number of CpG sites in a gene. This means that genes with higher GC content tend to have more CpG sites and vice versa. A calculation of G+C content of the genomes presented herein (Table 4) shows that myxozoans species have genomes ranging in G+C content as low as 16.71% in the case of *M. honghuensis*, and up to 51.19% in *Ellipsomyxa* sp. sample 70. The myxozoan genome assemblies were divided into CDS and non-CDS sequences. Table 4 shows that consistently in all genomes analysed, the CDS regions are characterized by increased GC content accompanied by more CpG sites when compared to non-CDS regions. The ratio of observed vs expected number of CpG sites serves as an indicator of DNA methylation (the expected number assumes that all cytosine and guanine residues within a sequence will be methylated). Based upon this assumption, Python implemented GMM was conducted on all assembly CDS calculated CpG O/E data. Component means distances ≥ 0.25 were interpreted as indicative of DNA methylation in the modelling results [64]. This stringent threshold was selected to exclude false positives. The analysis indicated that 9 of 13 myxozoans are predicted to have methylation capabilities (Tables 3 and 4).

There were 2 borderline cases, with component mean distances of 0.247 and 0.2316. *Ellipsomyxa* sp. sample 70, *Ellipsomyxa* sp. sample 115 and Myxosporea sp. sample 108 and *Thelohanellus kitauei* genomes have a value below 0.25, but still display slightly smaller average CpG O/E values in CDS genome sequences in comparison to non-CDS sequences, although the opposite was revealed for GC content. This indicated that even in these genomes associated with very low degree of experimentally recorded methylation (e.g., *Ellipsomyxa* sp. sample 70; Figure 7), there was depletion of CpG in coding sequences and this could also be an indicator of methylation.

Our Python implementation of a non-parametric KDE method revealed a striking pattern of bimodal methylation distribution across the myxozoan CDSs, further confirming the existence of two distinct groups of CDSs with differential methylation patterns (Figure 8A) MethMod results already indicated. These results confirm the parametric MethMod results. This bimodality suggested the presence of two distinct populations of coding sequences based on CpG O/E values, potentially reflecting different methylation states and/or biological processes (Figure 8B). The resulting probability density function curve (Figure 8B, shown in blue) clearly indicated two distinct peaks corresponding to the underlying distributions of CDSs. One peak with an CpG O/E of 0.60 and another of 1.04. The lower CpG O/E peak was expected to represent a methylated cluster of CDSs based upon the presumption that methylation causes CpG depletion, whilst the higher CpG O/E value represented the unmethylated cluster of CDSs. The weighted KDE probability density function curve (Figure 8B, shown in orange) also shows that the non-methylated portion of myxozoan genomes is not being affected by the addition of ONT weights, since the peak of this cluster changes only marginally (from 1.04 to 0.98 CpG O/E). However, since genes that

experimentally show signs of methylation have cumulative CpG site methylation frequencies that add significantly more weight, a much more dramatic effect on this cluster peak is observed (CpG O/E peak drops from 0.60 to 0.40). The underlying probability density function curve displays a reciprocal relationship between CpG O/E and ONT recorded methylation frequency when the weighted (orange) and unweighted (blue) curves are compared (Figure 8B), with a clear increase in density of the methylated cluster and a proportional decrease in the non-methylated cluster.

The cumulative methylation frequencies (based on experimental data obtained by ONT based Nanopolish methylation calling results) of the 1,000 most extreme CDSs with above average CpG O/E (marked "high" and shown in blue in Figure 9) and 1,000 most extreme below average CpG O/E CDSs (marked "low" and shown in red in Figure 9) are displayed on a smoothed plot (Figure 9). The 1,000 below average CpG O/E CDSs had a distinctly higher average methylation frequency (avg_low=0.52), while the 1,000 CDSs with higher average CpG O/E had on average, 11.2 times lower methylation frequency (avg_high=0.05). These results appear to be consistent and suggest that methylation causes CpG site depletion in the CDSs (which translates to smaller CpG O/E values for more frequently methylated genes).

Pearson's correlation analysis indicated a moderate negative linear relationship between the CpG O/E values for the CDSs and the recorded ONT Nanopore methylation frequencies ($r = -0.5028$ and $p=0.0$). Our analyses collectively indicate that CDS regions in Myxozoa are less variable and markedly more GC-rich compared to non-CDS regions. The fact that CDS regions of all the analysed genomes also have a higher degree of CpG regions compared to non-CDS regions, provides strong support that myxozoan CDS regions are more likely to be sites for CpG methylation. The CpG content in the context of myxozoan gene length was also analysed and a

linear relationship was found, further supporting this (Figure 10A). The CDS group with CpG O/E below the regression line represented the genes that are more likely to be methylated, while the group with CpG O/E above this line represented the genes most likely to be unmethylated (Figure 10A). This was performed on the myxozoan genome assemblies with MetaEuk predicted CDS sequences.

When myxozoan gene annotations were grouped based on corresponding CDS CpG O/E values (using the weighted KDE CpG O/E peaks as thresholds [Figure 8B] for selecting methylated/unmethylated CDSs), ontologies could be linked to the annotations within each group. Using keyword extraction methods, 200 keywords were selected that were able to capture and represent the GO inferred terms related to functions, processes and cellular compartments that best represent each group of CDSs (Figure 10B). When the intersecting terms common to all myxozoan genomes within each group were analyzed, it was discovered that genes at the low end of CpG O/E (the ones with high probability of being methylated - i.e., transcriptionally silenced) form a smaller intersection characterized by terms linked to activity (Figure 10B). On the opposite end, the high CpG O/E genes (most likely not being methylated - i.e., transcriptionally active) formed a larger intersection linked to transcription, translation and other activities that fit a description of housekeeping genes, notably including genes encoding methylation mechanisms.

DISCUSSION

New high quality myxozoan genome assemblies

Herein, we present the first comprehensive analysis of myxozoan DNA using Oxford Nanopore technology (ONT). This long-read sequencing technology coupled with

downstream bioinformatic processing generated assemblies of five myxozoan genomes, all free from detectable host DNA contamination. The sequences of the five species did not match any available myxozoan sequences deposited in the SILVA Release 138.1 non-redundant Small Subunit rRNA Database [53]. We have therefore designated the five myxozoan assemblies deposited in the NCBI database as: *Ceratomyxa* sp. sample 57-BR2022, *Ceratomyxa* sp. sample 58-BR2022, *Ellipsomyxa* sp. sample 70-BR2022, Myxosporea sp. sample 108-BR2022 and *Ellipsomyxa* sp. sample 115-BR2022.

QUAST and BUSCO metrics confirmed the exceptional quality of these five new assemblies, surpassing the assembly statistics of the eight existing myxozoan genomes available in public databases. Functional annotation of all five assembled genomes and reannotation of the 8 existing myxozoan genomes using the same pipeline provided uniformly annotated datasets for comparison. Annotations derived from BUSCO, PANTHER HMM, and Gene Ontology analyses revealed highly heterogenous protein annotations. Likely causes of this heterogeneity include short, highly fragmented reads and significant amounts of contaminant DNA in extant myxozoan assemblies. Our results suggest that such extant myxozoan genome assemblies should be re-sequenced using long-read technologies. To this end, it is worth noting that in our experience obtaining consistent high quality long sequence reads depends critically on the collection, storage and transport of myxozoan infected material and on DNA handling time. Future experimentation will focus on an in-field DNA extraction procedure akin to that achieved for microbial symbionts of marine invertebrates [70]. We therefore plan an in-field extraction procedure optimized beyond a crude template followed immediately by genome sequencing using Oxford Nanopore PromethION technology. This in-field approach has been used to

characterise specific genes for other parasites (e.g., malaria parasites [71] and *Blastocystis* spp. [72]) and should also enable generating genome data. It is important to highlight that host DNA contamination is almost inevitable in all genome sequencing attempts on myxozoans due to their endoparasitic lifestyles. This can be at least partially addressed/minimised by mitigating the ratio of host to parasite reads. We found that failure to address challenges relating to read quality and contamination may lead to false inferences of myxozoan genome sizes and phylogenetic relationships. Our approach to deal with this issue was to use minimap2 in order to map all reads onto a large set of reference fish genomes, and then assembling the non-mapping reads. These assembled contigs were then screened by BLAST comparison against comprehensive databases such as NT and tsa_NR in order to remove even the slightest remaining homologies to fish hosts.

<u>Robust methylation detection</u>

The experimental approaches we took to both determine and validate cytosine methylation at CpG sites across all available myxozoan genome assemblies counter the assertion that myxosporeans are amongst the few animals that have secondarily lost cytosine methylation capability [10]. Our analyses indicated that methylation is indeed present in most myxozoan genomes based on experimentally obtained ONT Nanopore methylation calling and by *in silico* modelling component means distance criteria. The model-obtained observation was further validated and confirmed by a non-parametric method, the Kernel Density Estimation. The relationship between methylation and genome defence in Cnidaria [73] would predict that methylated genes are significantly longer than unmethylated genes. We therefore analysed CpG content in the context of myxozoan gene length using *Ellipsomyxa* sp. sample 70 as the best available myxozoan assembly with limited to no contamination. Accordingly, we found

a linear relationship between gene length and number of methylation targets (i.e., the longer the gene, the more methylation targets [CpG sites] it possessed) as expected for cnidarians. The relationship was so straightforward that it could be used to predict the number of CpG sites purely based on gene length.

GC content and DNA methylation

It is known that GC-rich regions provide more targets for methylation and show a greater frequency of methylated sites than GC-poor regions [74]. Usually, a telltale mark of genome methylation is loss of CpG dinucleotide sites, referred to as CpG depletion (CpG O/E) [75], which results due to increased mutation in methylated CpGs. The value is based on the observed frequency of CpG dinucleotides relative to the product of the frequency of the individual nucleotides (G and C) weighted by the length of a genomic region [75]. This ratio is notably low in vertebrate genomes (e.g. < 0.20 in the human genome [76]). Invertebrate genomes on the other hand are known to be much less methylated overall. Their methylation pattern is often described as "mosaic-like" [5, 77] - with some CDSs experiencing frequent methylation and others none at all. Since the general invertebrate methylation pattern includes gene bodies instead of non-CDS promoter sites, we divided the myxozoan genome assemblies accordingly and confirmed that Myxozoa are no exception to this. For all the myxozoan genomes analysed the CDS sequences are characterized by increased GC content accompanied by more CpG sites when compared to non-CDS regions. Research on two sponges, a cnidarian and a ctenophore provide evidence that DNA methylation was present in early metazoans and has largely been conserved [78]. Although the CpG methylation system has apparently been lost in some invertebrates, most notably *C. elegans* [79], for others, like *Drosophila,* this has been disputed after the initial claims [80]. We suggest that invertebrates currently believed to possess no

methylation may nevertheless employ this epigenetic process, albeit not in the expected way.

The most plausible explanation for the observed CpG O/E drop in bimodally distributed CDS clusters that we identified in myxozoan genomes appears to relate to the DNA methylation process. Determining the overall genomic CDS CpG O/E ratio is a very significant indicator of DNA methylation for the following reasons. CDSs that are often methylated will inevitably display markedly lower CpG O/E values than sequences that are not methylated. This is due to an increased rate of mutation in methylated CpG that leads to GC content loss, with C-to-T transitions being the most common type of mutation at these sites. This GC loss will lead to a characteristic two-peak bimodal distribution of CpG/OE values, and which has been observed in all invertebrate species known to methylate DNA and herein (Figure 8). Additionally, when the distributions of the ratio between CDS and non-CDS regions of the genomes were compared, most suspected methylation events occurred in CDS regions as expected, since this is a tell-tale mark of invertebrate methylation (unlike in vertebrates which skip non-CDS regions). The GC content has significant biological consequences reflected in gene inactivation and genome instability that could explain reported variation in Myxozoa genome sizes. Except for the partial genomes of *Ceratomyxa* sp. sample 57 and *Ceratomyxa* sp. sample 58, myxozoan genomes are characterised by a GC content of less than 40%. Although the overall genome GC content is highly variable, the CDS sequences were less variable and markedly more GC-rich than sequences in non-CDS regions. This CDS and non-CDS GC content dichotomy within myxozoan genomes is likely a result of a complex interplay between functional requirements, mutational biases, repair mechanisms, recombination patterns, and evolutionary forces. Evidence that myxozoan CDS sequences are actively methylated

in myxozoans includes their greater GC content, a higher frequency of CpG regions in CDS than in non-CDS sequences in all analysed genomes, and two distinct subpopulations with markedly different CpG O/E values. Another study has observed that methylated invertebrate genes generally encode for housekeeping functions related to transcription and translation, whilst non-methylated genes generally encode for functions including cellular signalling and reproductive processes [81]. This observation was also indicated in myxozoans based on our GO analyses, which leads to new questions regarding the enzymatic machinery behind the methylation process acting upon these distinct groups of CDSs.

DNA methylation mechanisms and patterns

Our data have enabled us to demonstrate that there is extensive methylation in some genomes (*Ceratomyxa* sp. samples 57 and 58) and low levels of methylation in others (Myxosporea sp. sample 108, *Ellipsomyxa* sp. sample 115, and *Ellipsomyxa* sp. sample 70). Our epigenetic data and the CpG O/E values calculated from the genome assemblies in the form of a weighted KDE, enabled us to more precisely define CpG O/E boundaries and more accurately pinpoint which CDSs were actively methylated. We believe this is the first study to use epigenetic methylation frequency results in combination with genomic derived CpG O/E values to assess the frequency of methylation at specific genetic loci. This approach has potential as a preliminary screening method to identify genes of interest for targeted studies to resolve epigenetic processes, including regulatory mechanisms. To further examine DNA methylation mechanisms, we searched for the presence of DNMT, TET and BER related enzymes in the annotated genomes. The presence of such enzymes would be a strong function-related indicator for the potential to methylate/demethylate DNA. Both PANTHER HMM based searches and MetaEuk protein annotation indicated that

all three classes of methylation relevant enzymes were present. Our results demonstrate that BER is the most abundant methylation component, followed by DNMT and finally TET. Functional annotation has thus revealed the presence of key DNA methylation enzymes (DNMTs, TETs, BERs) in all analysed myxozoan genomes. However, traces of contamination in some previously sequenced myxozoan genomes were detected. This again highlights the importance of rigorous scrutiny and filtering when performing sequencing and assembly of myxozoans.

Overall, our ONT-based experimental data revealed a striking feature – either the overwhelming presence or near complete absence of methylation across five Myxozoa genomes, with samples *Ceratomyxa* sp. sample 57 and *Ceratomyxa* sp. sample 58 exhibiting extremely high levels of methylation, and Myxosporea sp. sample 108, *Ellipsomyxa* sp. sample 115 and *Ellipsomyxa* sp. sample 70 only marginal ones (Figure 7). However, all these genomes were strongly predicted to have distinct populations of CDSs being methylated by our *in-silico* analyses. This observation might also explain the conclusions reached by Kyger et al. [10]. If the DNA templates sequenced in that study were obtained from specimens at a point in a parasite life stage when the majority of genes were upregulated, then little if any methylation would be detected. This might also explain our results for *Ceratomyxa* sp. sample 57 and *Ceratomyxa* sp. sample 58. We note that high numbers of mature spores were particularly evident microscopically for *Ceratomyxa* sp. sample 57 and *Ceratomyxa* sp. sample 58 (Adriano, pers. obs). Perhaps extensive downregulation characterises this period of development. Future experimentation is warranted to examine whether there may be some myxozoan life history stages when nearly universal genome methylation is required (“switched on”). Examining patterns of genome methylation will

provide valuable new insights into the epigenetic regulation of myxozoan development and host-parasite interactions.

CONCLUSIONS

Our comprehensive analysis of contiguous long sequence reads provide the first evidence for gene body methylation in Myxozoa. We found that DNA methylation was generally either overwhelmingly present or almost completely absent in the material analysed. *In silico* analyses, based on GC content modelling and empirically derived methylation frequencies, consistently identified methylation as a factor in shaping genomic landscapes of myxozoans. The majority of analysed genomes display clear signs of methylation taking place within gene bodies, based on evidence in the form of distinctly lower CpG O/E gene groups. Further examination of the associated genomic architecture, mechanisms and timing of epigenetic control of gene transcription in myxozoans will expand our understanding of development and parasite-host-environment interactions. It may also enable novel control strategies for these important fish pathogens.

ACKNOWLEDGEMENTS

This work was supported by Fundação de Amparo à Pesquisa do Estado de São Paulo and King's College London, Regular Research Award (APR), 2019/17427-3, E A Adriano; Fundação de Amparo à Pesquisa do Estado de São Paulo and King's College London, Regular Research Award (APR), 2019/17427-3, P F Long; Fundação de Amparo à Pesquisa do Estado de São Paulo and King's College London, Regular Research Award (APR), AC15137, E A Adriano; Fundação de Amparo à Pesquisa do Estado de São Paulo and King's College London, Regular Research Award (APR),

AC15137, P F Long. Fundação de Amparo à Pesquisa do Estado de São Paulo, Thematic Research Award, #2023/06420-3, E A Adriano; Coordenação de Aperfeiçoamento de Pessoal de Nível Superior (CAPES), Research Fellowship, Finance Code 001, E A Adriano; Conselho Nacional de Desenvolvimento Científico e Tecnológico (CNPq), Research Fellowship, 307485/2023-4, E A Adriano; Fundação de Amparo à Pesquisa do Estado de São Paulo, PhD Fellowship, #2023/06420-3, R T A Figueredo. We extend our thanks for the technical assistance and customer support offered by Novogene Europe, especially Yuwei Jiao, Shahudul Uddin and Xinzhu Zhang. We also thank the fishermen: Fernando Dias de Souza, Francisco dos Santos Pinto, and Arlindo Teixeira Guimarães for their local knowledge of fish and for providing study material from the Amazon and Tapajós rivers.

DATA AVAILABILITY

The five myxozoan assemblies, clean from any detectable contamination are deposited in the NCBI database as:

SUBID	BioProject	BioSample	Accession	Organism
SUB14430520	PRJNA1107121	SAMN41159988	JBDJAL000000000	*Ceratomyxa* sp. 57-BR2022
SUB14430520	PRJNA1107121	SAMN41159989	JBDJAM000000000	*Ceratomyxa* sp. 58-BR2022
SUB14430520	PRJNA1107121	SAMN41159990	JBDJAN000000000	Myxosporea sp. 108-BR2022

SUB14430520	PRJNA1107121	SAMN41159991	JBDJAO000000000	*Ellipsomyxa* sp. 115-BR2022
SUB14430520	PRJNA1107121	SAMN41159961	JBDJAP000000000	*Ellipsomyxa* sp. 70-BR2022

All helper scripts used for Myxozoa data processing are publicly available on GitHub [https://github.com/astarsky2016/Myxozoa_supplementary]. More detailed outputs of genome assembly metrics comparison, protein annotations and Gene Ontology Enrichment analysis can be found on publicly available Mendelay Data repository and as GigaDB dataset on dedicated FTP directory (reference below)

Mendelay Data reference:

Starcevic, Antonio; Figueredo, Rayline; Naldoni, Juliana; Corrêa, Lincoln; Okamura, Beth; Adriano, Edson; Long, Paul (2024), “Gene body methylation in Myxozoa”, Mendeley Data, V1, doi: 10.17632/dzdnwnxyrv.1

BENEFIT SHARING STATEMENT

A research collaboration was developed with scientists from the countries providing genetic samples, all collaborators are included as co-authors, the results of research have been shared with the provider communities and the broader scientific community, and the research addresses a priority concern, in this case the conservation of organisms being studied. More broadly, our group is committed to international scientific partnerships, as well as institutional capacity building.

AUTHOR CONTRIBUTIONS

A.S., E.A.A., B.O. and P.F.L. designed research; A.S., R.T.A.F., J. N., L.L.C., E.A.A. and P.F.L. performed research; A.S., E.A.A., B.O., and P.F.L. analysed data; A.S., E.A.A., B.O., and P.F.L. wrote the paper.

REFERENCES

[1] Goldberg AD, Allis CD, Bernstein E. Epigenetics: a landscape takes shape. Cell. 2007;128(4):635-8. doi: 10.1016/j.cell.2007.02.006

[2] Bogdanović O, Veenstra GJ. DNA methylation and methyl-CpG binding proteins: developmental requirements and function. Chromosoma. 2009;118(5):549-65. doi: 10.1007/s00412-009-0221-9.

[3] Klose RJ, Bird AP. Genomic DNA methylation: the mark and its mediators. Trends Biochem Sci. 2006;31(2):89-97. doi: 10.1016/j.tibs.2005.12.008

[4] Altun G, Loring JF, Laurent LC. DNA methylation in embryonic stem cells. J Cell Biochem. 2010;109(1):1-6. doi: 10.1002/jcb.22374.

[5] Zemach A, McDaniel IE, Silva P, Zilberman D. Genome-wide evolutionary analysis of eukaryotic DNA methylation. Science. 2010;328(5980):916-9. doi: 10.1126/science.1186366.

[6] Jin J, Lian T, Gu C, Yu K, Gao YQ, Su XD. The effects of cytosine methylation on general transcription factors. Sci Rep. 2016;6:29119. doi: 10.1038/srep29119

[7] Cedar H, Bergman Y. Linking DNA methylation and histone modification: patterns and paradigms. Nat Rev Genet. 2009;10(5):295-304. doi: 10.1038/nrg2540.

[8] Richa R, Sinha RP. Hydroxymethylation of DNA: an epigenetic marker. EXCLI J. 2014;13:592-610.

[9] Wang X, Song W, Ji G, Song Y, Liu X, Luo X, et al. Regulation of DNA methylation on key parasitism genes of *Cysticercus cellulosae* revealed by integrative epigenomic-transcriptomic analyses. Hereditas. 2021;158(1):28. doi: 10.1186/s41065-021-00195-9.

[10] Kyger R, Luzuriaga-Neira A, Layman T, Milkewitz Sandberg TO, Singh D, Huchon D, et al. Myxosporea (Myxozoa, Cnidaria) Lack DNA Cytosine Methylation. Mol Biol Evol. 2021;38(2):393-404. doi: 10.1093/molbev/msaa214.

[11] Varma SJ, Calvani E, Grüning NM, Messner CB, Grayson N, Capuano F, et al. Global analysis of cytosine and adenine DNA modifications across the tree of life. eLife. 2022;11:e81002. doi: 10.7554/eLife.81002.

[12] Greer EL, Blanco MA, Gu L, Sendinc E, Liu J, Aristizábal-Corrales D, et al. DNA Methylation on N6-Adenine in *C. elegans*. Cell. 2015;161(4):868-78. doi: 10.1016/j.cell.2015.04.005.

[13] Bewick AJ, Vogel KJ, Moore AJ, Schmitz RJ. Evolution of DNA Methylation across Insects. Mol Biol Evol. 2017;34(3):654-665. doi: 10.1093/molbev/msw264. Erratum in: Mol Biol Evol. 2017 Apr 1;34(4):1025.

[14] Geyer KK, Chalmers IW, Mackintosh N, Hirst JE, Geoghegan R, Badets M, et al. Cytosine methylation is a conserved epigenetic feature found throughout the phylum *Platyhelminthes*. BMC Genomics. 2013;14:462. doi: 10.1186/1471-2164-14-462.

[15] Raddatz G, Guzzardo PM, Olova N, Fantappié MR, Rampp M, Schaefer M, et al. Dnmt2-dependent methylomes lack defined DNA methylation patterns. Proc Natl Acad Sci U S A. 2013;110(21):8627-31. doi: 10.1073/pnas.1306723110.

[16] Ratel D, Ravanat JL, Berger F, Wion D. N6-methyladenine: the other methylated base of DNA. BioEssays. 2006;28(3):309-15. doi: 10.1002/bies.20342.

[17] Zhang G, Huang H, Liu D, Cheng Y, Liu X, Zhang W, et al. N6-methyladenine DNA modification in *Drosophila*. Cell. 2015;161(4):893-906. doi: 10.1016/j.cell.2015.04.018.

[18] Wu TP, Wang T, Seetin MG, Lai Y, Zhu S, Lin K, et al. DNA methylation on N(6)-adenine in mammalian embryonic stem cells. Nature. 2016;532(7599):329-33. doi: 10.1038/nature17640.

[19] Liang Z, Shen L, Cui X, Bao S, Geng Y, Yu G, et al. DNA N6-Adenine Methylation in *Arabidopsis thaliana*. Dev Cell. 2018;45(3):406-416.e3. doi: 10.1016/j.devcel.2018.03.012.

[20] Sullivan WJ Jr, Naguleswaran A, Angel SO. Histones and histone modifications in protozoan parasites. Cell Microbiol. 2006;8(12):1850-61. doi: 10.1111/j.1462-5822.2006.00818.x.

[21] Zhang Y, Sun Z, Jia J, Du T, Zhang N, Tang Y, et al. Overview of histone modification. Adv Exp Med Biol. 2021;1283:1-16. doi: 10.1007/978-981-15-8104-5_1.

[22] Miao J, Fan Q, Cui L, Li J, Li J, Cui L. The malaria parasite *Plasmodium falciparum* histones: organization, expression, and acetylation. Gene. 2006;369:53-65. doi: 10.1016/j.gene.2005.10.022.

[23] Soto M, Requena JM, Quijada L, Alonso C. Organization, transcription and regulation of the *Leishmania infantum* histone H3 genes. Biochem J. 1996;318(3):813-9. doi: 10.1042/bj3180813.

[24] García-Salcedo JA, Oliver JL, Stock RP, González A. Molecular characterization and transcription of the histone H2B gene from the protozoan parasite *Trypanosoma cruzi*. Mol Microbiol. 1994;13(6):1033-43. doi: 10.1111/j.1365-2958.1994.tb00494.x.

[25] Maor-Landaw K, Avidor I, Rostowsky N, Salti B, Smirnov M, Ofek-Lalzar M, et al. The molecular mechanisms employed by the parasite *Myxobolus bejeranoi* (Cnidaria: Myxozoa) from invasion through sporulation for successful proliferation in its fish host. Int. J. Mol. Sci. 2023;12824. doi.org/10.3390/ijms241612824.

[26] Bağcı ÖU, Caner A. The role of microRNAs in parasitology. Turkiye Parazitol Derg. 2020;44(2):102-108. doi: 10.4274/tpd.galenos.2020.6776.

[27] Rojas-Pirela M, Andrade-Alviárez D, Medina L, Castillo C, Liempi A, Guerrero-Muñoz J, et al. MicroRNAs: master regulators in host-parasitic protist interactions. Open Biol. 2022;12(6):210395. doi: 10.1098/rsob.210395.

[28] Liu X, Luo M, Wu K. Epigenetic interplay of histone modifications and DNA methylation mediated by HDA6. Plant Signal Behav. 2012;7(6):633-5. doi: 10.4161/psb.19994.

[29] Du Q, Luu PL, Stirzaker C, Clark SJ. Methyl-CpG-binding domain proteins: readers of the epigenome. Epigenomics. 2015;7(6):1051-73. doi: 10.2217/epi.15.39.

[30] Fuks F, Hurd PJ, Wolf D, Nan X, Bird AP, Kouzarides T. The methyl-CpG-binding protein MeCP2 links DNA methylation to histone methylation. J Biol Chem. 2003;278(6):4035-40. doi: 10.1074/jbc.M210256200.

[31] Lee JY, Lee TH. Effects of histone acetylation and CpG methylation on the structure of nucleosomes. Biochim Biophys Acta. 2012;1824(8):974-82. doi: 10.1016/j.bbapap.2012.05.006.

[32] Lom J, Dyková I. Myxozoan genera: definition and notes on taxonomy, life-cycle terminology and pathogenic species. Folia Parasitol (Praha). 2006;53(1):1-36.

[33] Okamura B, Hartigan A, Naldoni J. Extensive Uncharted Biodiversity: The parasite dimension. Integr Comp Biol. 2018;58(6):1132-1145. doi: 10.1093/icb/icy039.

[34] Genereux DP, Johnson WC, Burden AF, Stöger R, Laird CD. Errors in the bisulfite conversion of DNA: modulating inappropriate- and failed-conversion frequencies. Nucleic Acids Res. 2008;36(22):e150. doi: 10.1093/nar/gkn691

[35] Quince C, Walker AW, Simpson JT, Loman NJ, Segata N. Shotgun metagenomics, from sampling to analysis. Nat Biotechnol. 2017;35(9):833-844. doi: 10.1038/nbt.3935

[36] Houseman EA, Accomando WP, Koestler DC, Christensen BC, Marsit CJ, Nelson HH, et al. DNA methylation arrays as surrogate measures of cell mixture distribution. BMC Bioinformatics. 2012;13:86. doi: 10.1186/1471-2105-13-86

[37] Okamura B, Gruhl A, Bartholomew JL. An Introduction to myxozoan evolution, ecology and development. In: Okamura B, Gruhl A, Bartholomew JL, editors. Myxozoan Evolution, Ecology and Development. Springer International Publishing, Cham, 2015. p1–20.

[38] Atkinson SD, Bartholomew JL, Lotan T. Myxozoans: Ancient metazoan parasites find a home in phylum Cnidaria. Zoology (Jena);129:66-68. doi: 10.1016/j.zool.2018.06.005.

[39] Jiménez-Guri E, Philippe H, Okamura B, Holland PW. *Buddenbrockia* is a cnidarian worm. Science. 2007; 317(5834):116-8. doi: 10.1126/science.

[40] Collins AG (2009) Recent insights into cnidarian phylogeny. In: Lang, MA et al Proceedings of the smithsonian marine science symposium. Smithsonian Contributions to the Marine Sciences 38:139–149

[41] Holland JW, Okamura B, Hartikainen H, Secombes CJ. A novel minicollagen gene links cnidarians and myxozoans. Proc Biol Sci. 2011;278(1705):546-53. doi: 10.1098/rspb.2010.1301.

[42] Okamura, B., & Gruhl, A. Evolution, origins and diversification of parasitic cnidarians. In The Evolution and Fossil Record of Parasitism: Identification and Macroevolution of Parasites. Cham: Springer International Publishing, 2021. p.109-152.

[43] Okamura B, Gruhl A, Reft AI. Cnidarians origins of the Myxozoa. In: Okamura B, Gruhl A, Bartholomew JL, editors. Myxozoan Evolution, Ecology and Development. Springer International Publishing, Cham, 2015. p.45–68.

[44] Adriano EA, Zatti SA, Okamura B. How to build single-celled cnidarians with worm-like motility: Lessons from Myxozoa. J Anat. 2022;240(3):475-488. doi: 10.1111/joa.13566.

[45] Simpson JT, Workman RE, Zuzarte PC, David M, Dursi LJ, Timp W. Detecting DNA cytosine methylation using nanopore sequencing. Nat Methods. 2017;14(4):407-410. doi: 10.1038/nmeth.4184

[46] Li H. Minimap2: pairwise alignment for nucleotide sequences. Bioinformatics. 2018;34(18):3094-3100. doi: 10.1093/bioinformatics/bty191.

[47] Kolmogorov M, Yuan J, Lin Y, Pevzner PA. Assembly of long, error-prone reads using repeat graphs. Nat Biotechnol. 2019;37(5):540-46. doi: 10.1038/s41587-019-0072-8.

[48] Challis R, Richards E, Rajan J, Cochrane G, Blaxter M. BlobToolKit - Interactive Quality Assessment of Genome Assemblies. G3 (Bethesda). 2020;10(4):1361-1374. doi: 10.1534/g3.119.400908

[49] Altschul SF, Gish W, Miller W, Myers EW, Lipman DJ. Basic local alignment search tool. J Mol Biol. 1990;215(3):403-10. doi: 10.1016/S0022-2836(05)80360-2.

[50] Buchfink B, Reuter K, Drost HG. Sensitive protein alignments at tree-of-life scale using DIAMOND. Nat Methods. 2021;18(4):366-68. doi: 10.1038/s41592-021-01101-x.

[51] Marçais G, Kingsford C. A fast, lock-free approach for efficient parallel counting of occurrences of k-mers. Bioinformatics. 2011;27(6):764-70. doi: 10.1093/bioinformatics/btr011.

[52] Koren S, Walenz BP, Berlin K, Miller JR, Bergman NH, Phillippy AM. Canu: scalable and accurate long-read assembly via adaptive *k*-mer weighting and repeat separation. Genome Res. 2017;27(5):722-36. doi: 10.1101/gr.215087.116.

[53] Quast C, Pruesse E, Yilmaz P, Gerken J, Schweer T, Yarza P, Peplies J, Glöckner FO. The SILVA ribosomal RNA gene database project: improved data processing and web-based tools. Nucleic Acids Res. 2013;41(Database issue):D590-6. doi: 10.1093/nar/gks1219.

[54] Yilmaz P, Parfrey LW, Yarza P, Gerken J, Pruesse E, Quast C, Schweer T, Peplies J, Ludwig W, Glöckner FO. The SILVA and "All-species Living Tree Project (LTP)" taxonomic frameworks. Nucleic Acids Res. 2014;42(Database issue):D643-8. doi: 10.1093/nar/gkt1209.

[55] Cannone JJ, Subramanian S, Schnare MN, Collett JR, D'Souza LM, Du Y, Feng B, Lin N, Madabusi LV, Müller KM, Pande N, Shang Z, Yu N, Gutell RR. The comparative RNA web (CRW) site: an online database of comparative sequence and structure information for ribosomal, intron, and other RNAs. BMC Bioinformatics. 2002;3:2. doi: 10.1186/1471-2105-3-2.

[56] Minh BQ, Schmidt HA, Chernomor O, Schrempf D, Woodhams MD, von Haeseler A, Lanfear R. IQ-TREE 2: New Models and Efficient Methods for Phylogenetic Inference in the Genomic Era. Mol Biol Evol. 2020;37(5):1530-1534. doi: 10.1093/molbev/msaa015.

[57] Kalyaanamoorthy S, Minh BQ, Wong TKF, von Haeseler A, Jermiin LS. ModelFinder: fast model selection for accurate phylogenetic estimates. Nat Methods. 2017;14(6):587-589. doi: 10.1038/nmeth.4285.

[58] Letunic I, Bork P. Interactive Tree of Life (iTOL) v6: recent updates to the phylogenetic tree display and annotation tool. Nucleic Acids Res. 2024;52(W1):W78-W82. doi: 10.1093/nar/gkae268.

[59] Gurevich A, Saveliev V, Vyahhi N, Tesler G. QUAST: quality assessment tool for genome assemblies. Bioinformatics. 2013;29(8):1072-5. doi: 10.1093/bioinformatics/btt086.

[60] Seppey M, Manni M, Zdobnov EM. BUSCO: Assessing genome assembly and annotation completeness. Methods Mol Biol. 2019;1962:227-45. doi: 10.1007/978-1-4939-9173-0_14.

[61] Kriventseva EV, Tegenfeldt F, Petty TJ, Waterhouse RM, Simão FA, Pozdnyakov IA, et al. OrthoDB v8: update of the hierarchical catalog of orthologs and the underlying free software. Nucleic Acids Res. 2015;43(Database issue):D250-6. doi: 10.1093/nar/gku1220.

[62] Waterhouse RM, Tegenfeldt F, Li J, Zdobnov EM, Kriventseva EV. OrthoDB: a hierarchical catalog of animal, fungal and bacterial orthologs. Nucleic Acids Res. 2013;41(Database issue):D358-65. doi: 10.1093/nar/gks1116.

[63] Liu Y, Rosikiewicz W, Pan Z, Jillette N, Wang P, Taghbalout A, et al. DNA methylation-calling tools for Oxford Nanopore sequencing: a survey and human epigenome-wide evaluation. Genome Biol. 2021;22(1):295. doi: 10.1186/s13059-021-02510-z.

[64] Levy Karin E, Mirdita M, Söding J. MetaEuk-sensitive, high-throughput gene discovery, and annotation for large-scale eukaryotic metagenomics. Microbiome. 2020;8(1):48. doi: 10.1186/s40168-020-00808-x.

[65] Steinegger M, Söding J. MMseqs2 enables sensitive protein sequence searching for the analysis of massive data sets. Nat Biotechnol. 2017;35(11):1026-28. doi: 10.1038/nbt.3988.

[66] Mi H, Muruganujan A, Casagrande JT, Thomas PD. Large-scale gene function analysis with the PANTHER classification system. Nat Protoc. 2013;8(8):1551-66. doi: 10.1038/nprot.2013.092.

[67] Engelhardt J, Scheer O, Stadler PF, Prohaska SJ. Evolution of DNA methylation across Ecdysozoa. J Mol Evol. 2022;90(1):56-72. doi: 10.1007/s00239-021-10042-0.

[68] Kryukov K, Imanishi T, Nakagawa S. Nanopore sequencing data analysis of 16S rRNA genes using the GenomeSync-GSTK system. Methods Mol Biol. 2023;2632:215-26. doi: 10.1007/978-1-0716-2996-3_15.

[69] Cabanettes F, Klopp C. D-GENIES: dot plot large genomes in an interactive, efficient and simple way. PeerJ. 2018;6:e4958. doi: 10.7717/peerj.4958.

[70] Dunlap WC, Battershill CN, Liptrot CH, Cobb RE, Bourne DG, Jaspars M et al. Biomedicinals from the phytosymbionts of marine invertebrates: a molecular approach. Methods. 2007;42(4):358-76. doi: 10.1016/j.ymeth.2007.03.001.

[71] Runtuwene LR, Tuda JSB, Mongan AE, Makalowski W, Frith MC, Imwong M, et al. Nanopore sequencing of drug-resistance-associated genes in malaria parasites, *Plasmodium falciparum*. Sci Rep. 2018;8(1):8286. doi: 10.1038/s41598-018-26334-3.

[72] Maloney JG, Molokin A, Santin M. Use of Oxford Nanopore MinION to generate full-length sequences of the *Blastocystis* small subunit (SSU) rRNA gene. Parasit Vectors. 2020;13(1):595. doi: 10.1186/s13071-020-04484-6.

[73] Ying H, Hayward DC, Klimovich A, Bosch TCG, Baldassarre L, Neeman T, et al. The role of DNA methylation in genome defense in Cnidaria and other invertebrates. Mol Biol Evol. 2022;39(2):msac018. doi: 10.1093/molbev/msac018.

[74] Cacciò S, Jabbari K, Matassi G, Guermonprez F, Desgrès J, Bernardi G. Methylation patterns in the isochores of vertebrate genomes. Gene. 1997;205(1-2):119-24. doi: 10.1016/s0378-1119(97)00560-x.

[75] Upadhyay M, Samal J, Kandpal M, Vasaikar S, Biswas B, Gomes J, et al. CpG dinucleotide frequencies reveal the role of host methylation capabilities in parvovirus evolution. J Virol. 2013;87(24):13816-24. doi: 10.1128/JVI.02515-13.

[76] Lander ES, et al. Initial sequencing and analysis of the human genome. Nature. 2001;409(6822):860-921. doi: 10.1038/35057062.

[77] Feng S, Cokus SJ, Zhang X, Chen PY, Bostick M, Goll MG, et al. Conservation and divergence of methylation patterning in plants and animals. Proc Natl Acad Sci U S A. 2010;107(19):8689-94. doi: 10.1073/pnas.1002720107.

[78] de Mendoza A, Hatleberg WL, Pang K, Leininger S, Bogdanovic O, Pflueger J, et al. Convergent evolution of a vertebrate-like methylome in a marine sponge. Nat Ecol Evol. 2019;3(10):1464-73. doi: 10.1038/s41559-019-0983-2.

[79] Simpson VJ, Johnson TE, Hammen RF. *Caenorhabditis elegans* DNA does not contain 5-methylcytosine at any time during development or aging. Nucleic Acids Res. 1986;14(16):6711-9. doi: 10.1093/nar/14.16.6711.

[80] Capuano F, Mülleder M, Kok R, Blom HJ, Ralser M. Cytosine DNA methylation is found in *Drosophila melanogaster* but absent in *Saccharomyces cerevisiae*, *Schizosaccharomyces pombe*, and other yeast species. Anal Chem. 2014;86(8):3697-702. doi: 10.1021/ac500447w.

[81] Sarda S, Zeng J, Hunt BG, Yi SV. The evolution of invertebrate gene body methylation. Mol Biol Evol. 2012;29(8):1907-16. doi: 10.1093/molbev/mss062.

Table 1 Click here to access/download;Table;Table 1 Identification of Myxozoa.docx

Table 1: Putative identification of Myxozoa. Identification was made using sequencing read comparison against an 18S rDNA database (SILVA) and visual analysis of morphological features using light microscopy.

**Specimen**	**Host**	**Identification based on best hit to the SILVA database**	**Identification based on microscopic morphology**
57	*Plagioscion squamosissimus*	*Ceratomyxa*	*Ceratomyxa*
58	*Plagioscion squamosissimus*	*Ceratomyxa*	*Ceratomyxa*
70	*Curimata inornata*	*Ceratomyxa*	*Ellipsomyxa*
108	*Rhaphiodon vulpinus*	*Ellipsomyxa* and *Myxidium*	*Ceratomyxa*
115	*Hemiodus unimaculatus*	*Ellipsomyxa*	*Ellipsomyxa and Ceratomyxa*

Click here to access/download;Table;Table 2 Predicted protein function encoded by core genes.docx

Table 2: Predicted protein functions by GO process encoded by a core repertoire of genes shared amongst 11 myxozoan genomes. Use of Named Entity Recognition (NER) in terms of keyword recognition and GO term analysis visualized using WordCloud methods enabled initial identification of gene categories with further analyses as described in Methods section. The terms being shared are not necessarily the most abundant GO categories in each individual genome annotation and WordCloud depictions.

GO Process	Shared GO terms
Molecular Function	GTP binding, nucleic acid binding, RNA binding, metal ion binding, hydrolase activity, ATP binding, DNA binding, ATP hydrolysis activity, zinc ion binding
Biological Process	DNA repair, proteolysis, protein phosphorylation, mRNA splicing via spliceosome, regulation of DNA-templated transcription, DNA replication, translation, phosphorylation
Cellular Component	mitochondrial inner membrane, Golgi membrane, DNA-directed RNA polymerase complex, endoplasmic reticulum membrane, nucleolus, plasma membrane, ribosome, microtubule, nucleus, spliceosomal complex, ribonucleoprotein complex, cytoplasm, cytosol, membrane

Click here to access/download;Table;Table 3_Genome distribution of methylation machinery.docx

Table 3: Distribution of GO biological processes of base-excision repair (BER), DNA methylation (DNMT) and DNA demethylation (TET) across 13 myxozoan genome assemblies. The presence of any genes falling into these three categories is denoted with a different colour: red (DNMT), blue (BER) and green (TET). Exact protein family members identified are labeled with Panther IDs in the third column and taxonomic assignments of identified proteins based on homology are given in the fourth column. The fifth column provides the distribution of identified protein family members across 12 Myxozoa genome annotations (ranging from 1 = unique to a genome, to 12 = appearing in all genomes)

Genome	Methylation machinery	Panther protein family identification	Example with closest homology	Number of gene copies
Myxobolus honghuensis	BER	PTHR11264:SF0	Eumetazoa	10
	BER	PTHR43286:SF1	*Thelohanellus kitauei*	11
	DNMT	PTHR45875:SF1	*Myxobolus squamalis*	6
	DNMT	PTHR10629	Aeromonas	1
	DNMT	PTHR10629:SF52	Bacteria	3
	TET	PTHR16557:SF2	*Henneguya salminicola*	6
Sample 108	BER	PTHR43286:SF1	Metazoa	11
	BER	PTHR11264:SF0	root	10
	TET	PTHR16557:SF2	Eukaryota	6
Ceratonova shasta	BER	PTHR43286:SF1	Opisthokonta	11
	BER	PTHR11264:SF0	Cellular organisms	10
	DNMT	PTHR45875:SF1	Eukaryota	6
	TET	PTHR16557:SF2	Eumetazoa	6
Sphaeromyxa zaharoni	BER	PTHR11264:SF0	Eumetazoa	10

	BER	PTHR43286:SF1	Eumetazoa	11
	BER	PTHR43286	Eumetazoa	3
	DNMT	PTHR45875:SF1	Eukaryota	6
Sample 70	BER	PTHR11264	Opisthokonta	3
	BER	PTHR43286:SF1	Eukaryota	11
	BER	PTHR43286	Eukaryota	3
	DNMT	PTHR12855	Eukaryota	3
	DNMT	PTHR45875:SF1	Eukaryota	6
	TET	PTHR16557:SF2	Opisthokonta	6
Sample 57	DNMT	PTHR10629:SF52	root	3
Sample 115	BER	PTHR11264:SF0	Eumetazoa	10
	BER	PTHR43286:SF1	Metazoa	11
	DNMT	PTHR10629:SF52	root	3
	TET	PTHR16557:SF2	Eukaryota	6
Thelohanellus kitauei	BER	PTHR43286:SF1	*Thelohanellus kitauei*	11
	BER	PTHR11264	Cellular organisms	3
	BER	PTHR42944	*Thelohanellus kitauei*	4
	BER	PTHR11264:SF0	Candidatus Marinimicrobia bacterium	10
	DNMT	PTHR12855	*Thelohanellus kitauei*	3

	DNMT	PTHR45875:SF1	*Henneguya salminicola*	6
	DNMT	PTHR10815:SF5	*Thelohanellus kitauei*	1
Enteromyxium leei	BER	PTHR43286:SF1	Metazoa	11
	BER	PTHR11264:SF0	*Sparus aurata*	10
	BER	PTHR43286	Metazoa	3
	TET	PTHR16557	Cellular organisms	3
Myxobolus squamalis	BER	PTHR42944	*Myxobolus squamalis*	4
	BER	PTHR43286:SF1	Eumetazoa	11
	BER	PTHR11264:SF0	Metazoa	10
	TET	PTHR16557:SF2	*Myxobolus squamalis*	6
Henneguya salminicola	BER	PTHR42944	*Henneguya salminicola*	4
	BER	PTHR43286:SF1	*Henneguya salminicola*	11
	BER	PTHR11264	*Myxobolus squamalis*	3
	BER	PTHR11264:SF0	Metazoa	10
	DNMT	PTHR45875:SF1	*Henneguya salminicola*	6
	DNMT	PTHR12855	*Myxobolus squamalis*	3
	TET	PTHR16557	*Henneguya salminicola*	3
Kudoa iwatai	BER	PTHR43286:SF1	Eumetazoa	11
	BER	PTHR11264:SF0	cellular organisms	10
	BER	PTHR42944	Eukaryota	4

	TET	PTHR16557	cellular organisms	3

Click here to access/download;Table;Table 4_G+C and CpG sites.docx

Table 4: Average G+C content of genome, CDS and non-CDS regions together with CpG sites calculated in 13 Myxozoa genome assemblies. Final column contains the distance (d) between the component means obtained using the GMM of a CpG Observed/Expected value distribution. Values of d >= 0.25 can be used to infer the presence of DNA methylation (marked in red).

Species	genome GC	CDS GC	CDS CpG O/E	Non-CDS GC	Non-CDS CpG O/E	CpG O/E means distance (d)
Sample 58	42.17%	44.34%	0.36	42.01%	0.34	0.3116
Sample 57	42.16%	44.54%	0.35	41.94%	0.39	0.3424
Sample 70	39.39%	51.19%	1.04	35.86%	1.04	0.1359
Enteromyxium leei	33.51%	42.29%	0.58	30.92%	0.49	0.3298
Sample 108	29.95%	32.20%	0.63	29.64%	0.70	0.247
Sample 115	29.95%	32.21%	0.65	29.44%	0.70	0.2535
Henneguya salminicola	28.96%	33.25%	0.98	28.98%	1.07	0.2838
Sphaeromyxa zaharoni	28.02%	35.09%	0.78	28.41%	0.91	0.2316
Myxobolus squamalis	27.30%	34.21%	0.84	28.40%	0.88	0.2939
Thelohanellus kitauei	25.50%	35.06%	1.00	23.45%	1.34	0.2098
Ceratonova	23.79%	31.08%	0.75	23.44%	0.87	0.3271

shasta						
Kudoa iwatai	23.64%	29.60%	0.54	23.08%	0.68	0.3073
Myxobolus honghuensis	16.71%	31.49%	1.18	17.37%	1.68	0.4356

Figure 1 Click here to access/download;Figure;Figure 1_Bioinformatics workflow.docx

Figure 1: Overview of the bioinformatics pipeline. Steps used to assemble myxozoan genomes from Oxford nanopore long sequence reads, and to detect the exact location of methylated bases (5-methylcytosine in a CpG context) within each assembled genome.

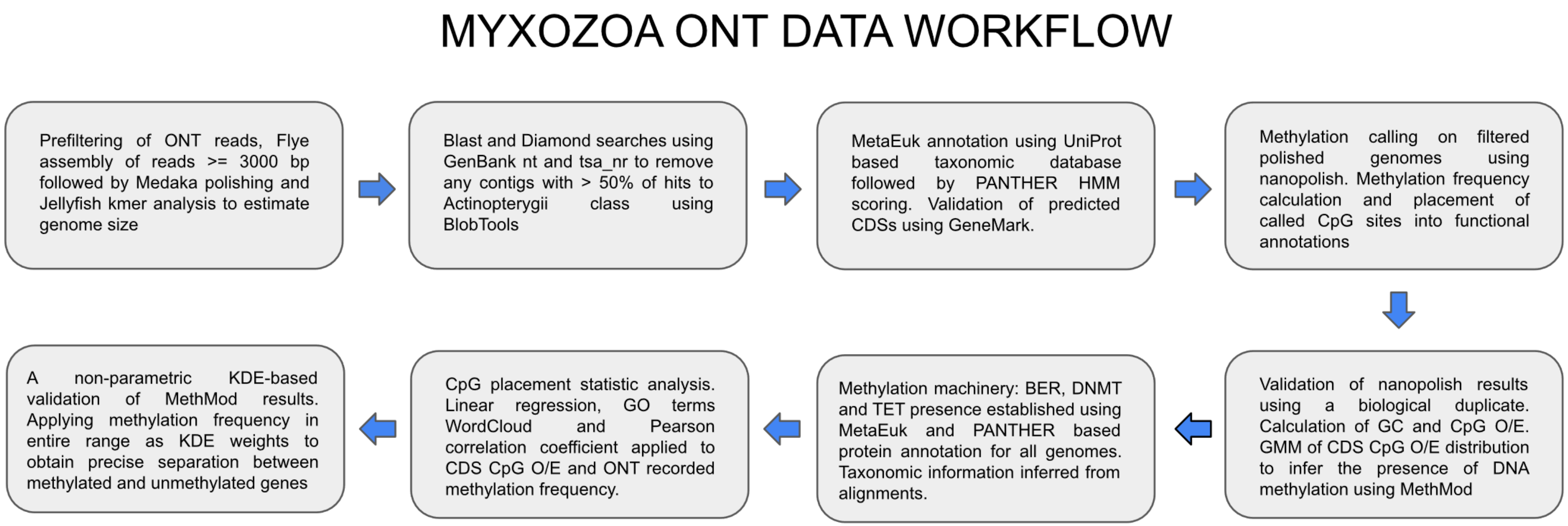

Click here to access/download;Figure;Figure 2_Genome synteny plots.docx

Figure 2: Whole genome dot-plot comparisons of (A) samples 57 and 58, (B) samples 108 and 115. Linearity implied a common ancestry with breaks in the plot indicative of genome fragmentation or deletions. More fragmented diagonal with higher density of lines off the main diagonal suggests that samples 108 and 115 (B) are less related in comparison with samples 57 and 58 (A) we believe to represent the same species.

(A)

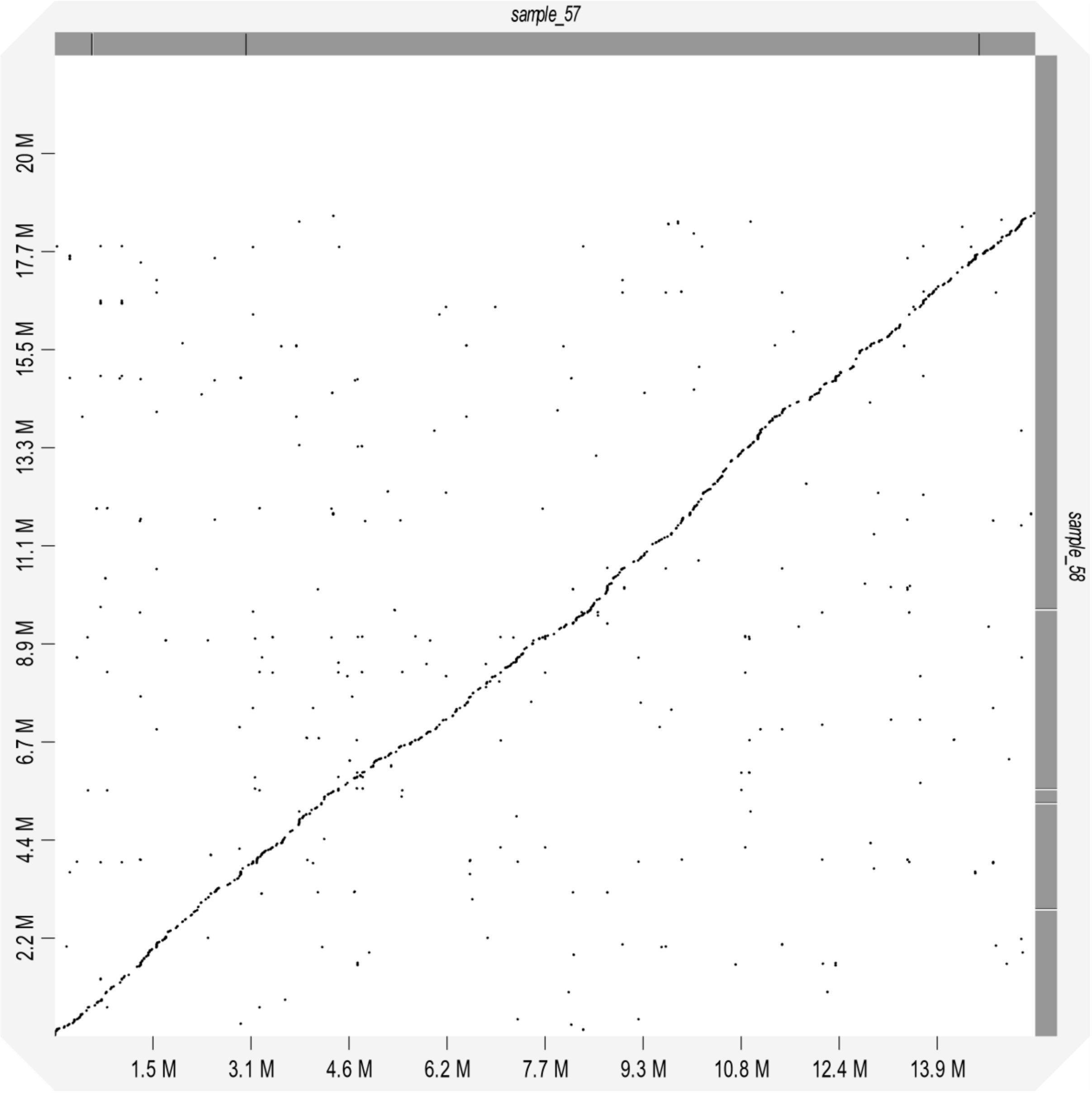

(B)

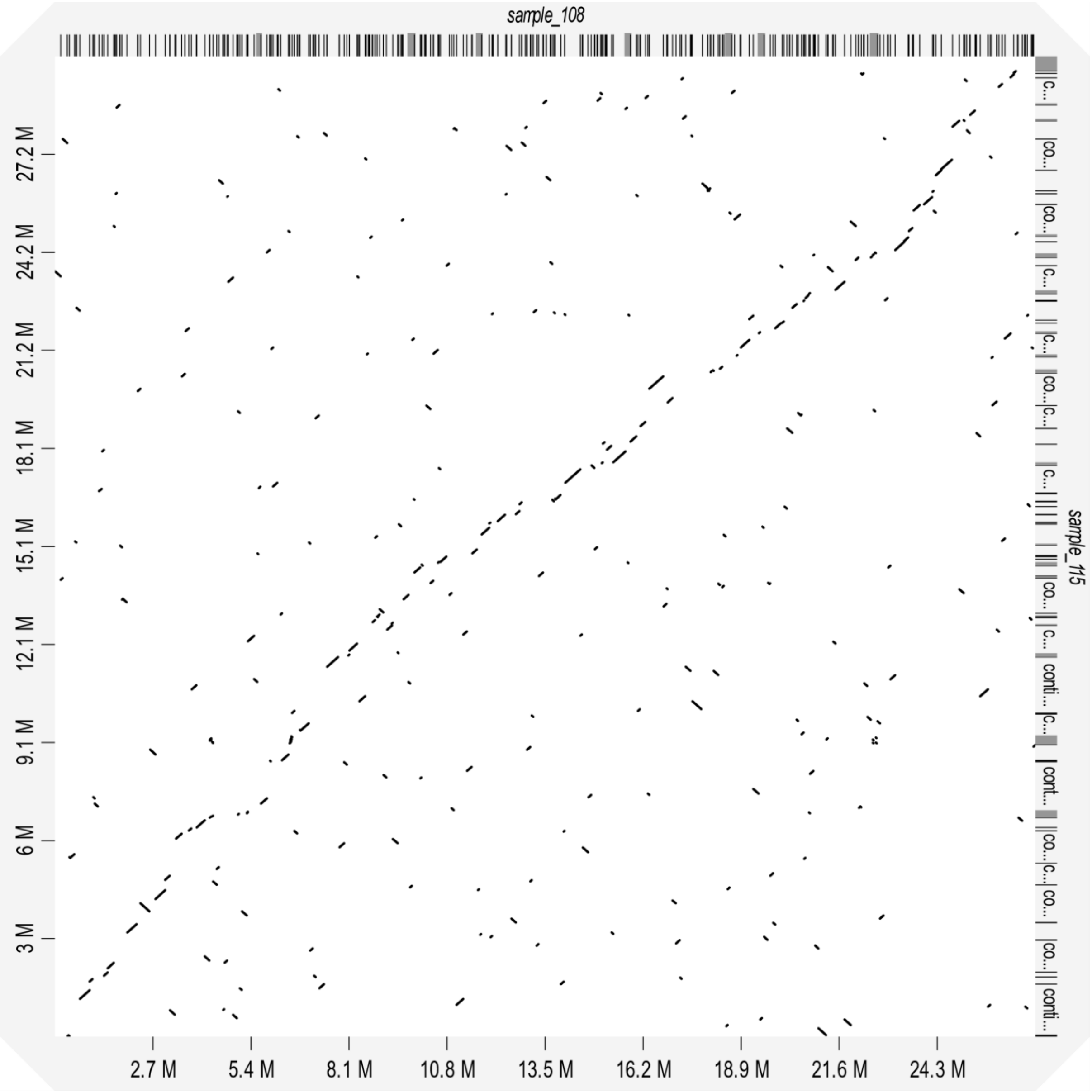
sample_108
sample_115
27.2 M
24.2 M
21.2 M
18.1 M
15.1 M
12.1 M
9.1 M
6 M
3 M
2.7 M
5.4 M
8.1 M
10.8 M
13.5 M
16.2 M
18.9 M
21.6 M
24.3 M

Figure 3

Click here to access/download;Figure;Figure 3 BUSCO analysis.docx

Figure 3: BUSCO assessment of genome assembly and annotation completeness for the 11 myxozoan genomes presently available. Despite a low level of shared BUSCOs overall, there was a small consensus of core proteins shared between the genomes.

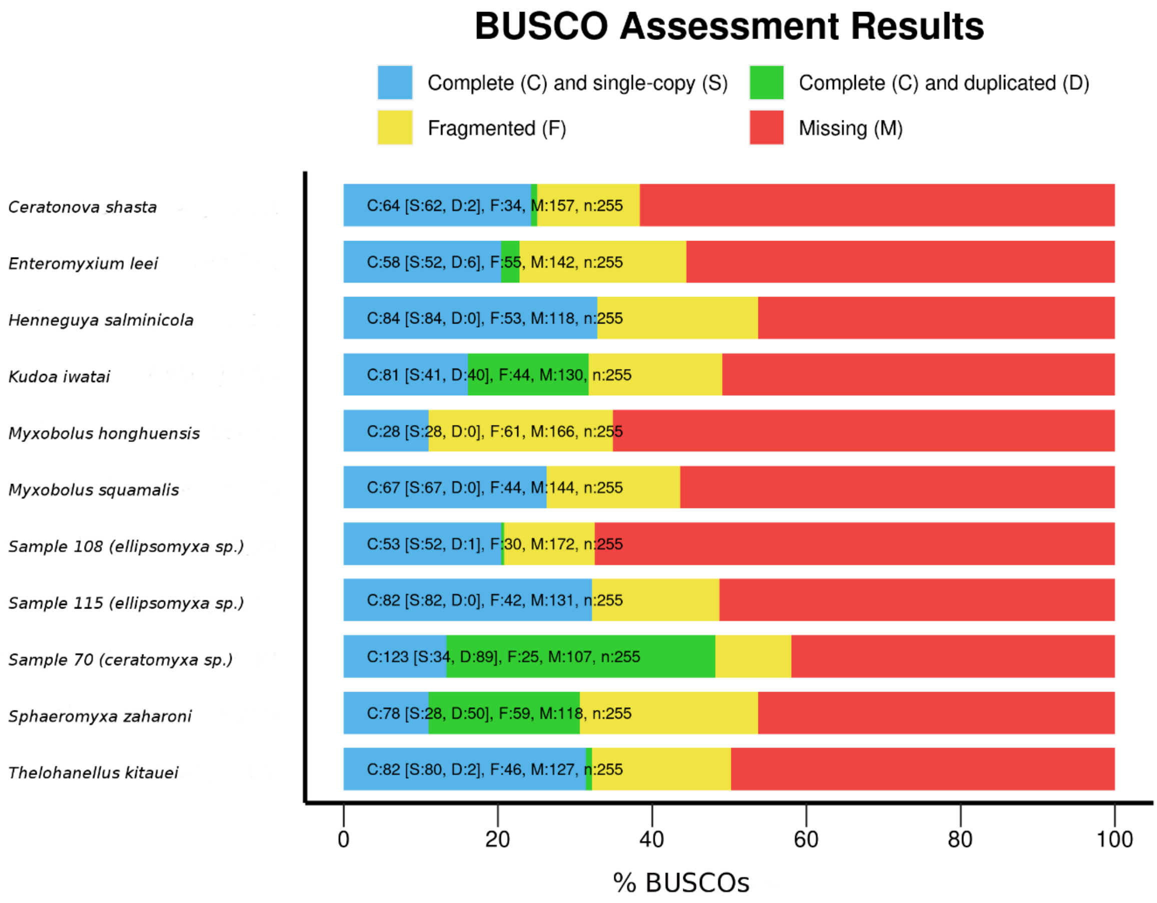

Figure 4

Click here to access/download;Figure;Figure 4 Venn diagram_new.docx

Figure 4: Comparison of the number of annotated genes shared between Samples 70, 108 and 115. Euler-Venn diagrams display the number of unique genes annotated by GO terms for A) Molecular function, B) Biological process and C) Cellular component.

A)

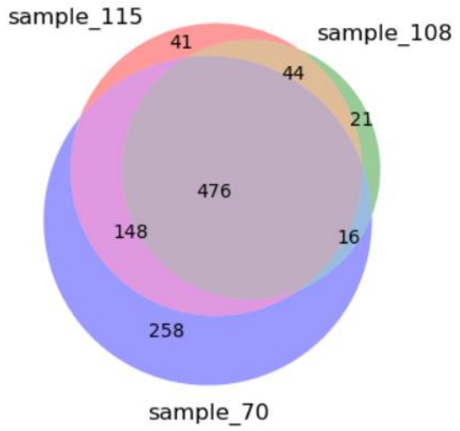

B)

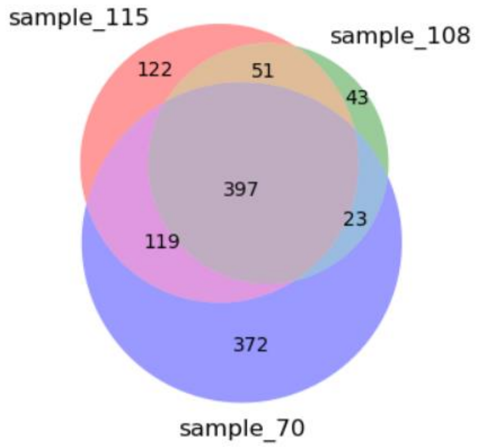

C)

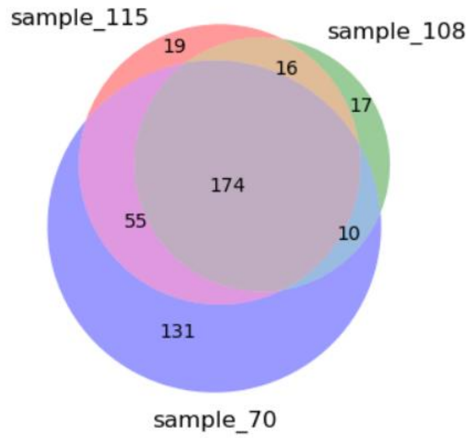

Figure 5 Click here to access/download;Figure;Figure 5_Comparative genome annotation.png

Figure 5: Pangenome annotation comparison between the eight Myxozoa genomes publicly available and the three assemblies produced in this study. This pangenome comparison shows that on the top level of GO annotations, Myxozoa do possess a conserved core gene repertoire (demarcated within the red lines).

A) Molecular function

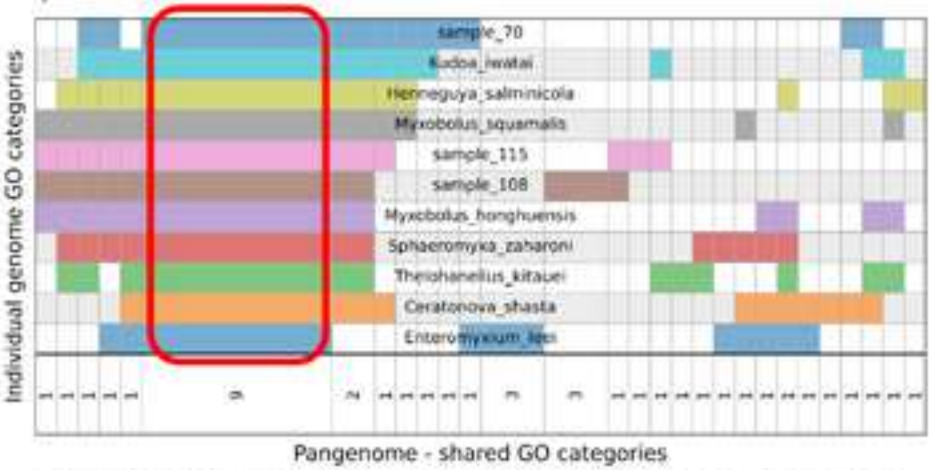

Shared GO: ATP binding, RNA binding, zinc ion binding, DNA binding, nucleic acid binding, hydrolase activity, metal ion binding, ATP hydrolysis activity, GTP binding

B) Biological process

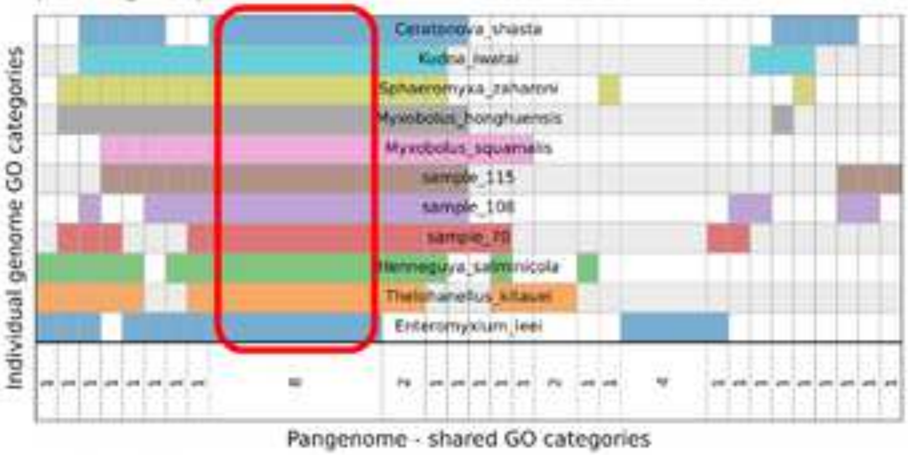

Shared GO: phosphorylation, DNA repair, regulation of DNA-templated transcription, proteolysis, mRNA splicing, via spliceosome, protein phosphorylation, DNA replication, translation

C) Cellular component

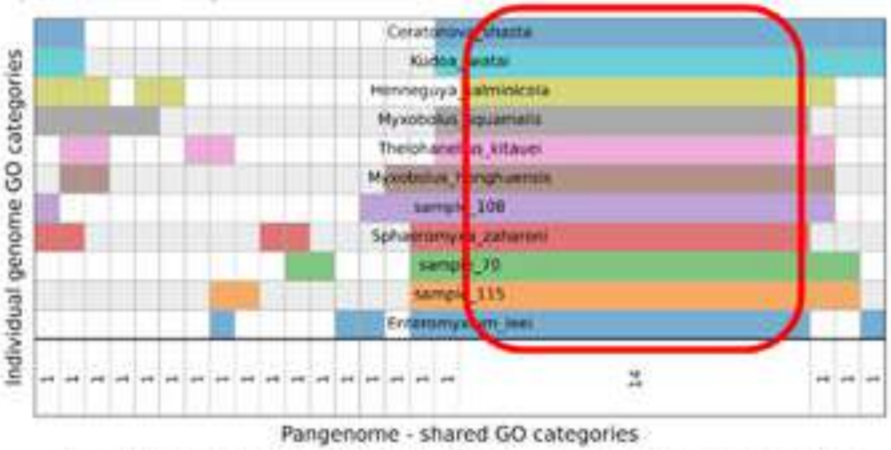

Shared GO: microtubule, DNA-directed RNA polymerase complex, ribosome, Golgi membrane, cytoplasm, plasma membrane, spliceosomal complex, membrane, endoplasmic reticulum membrane, mitochondrial inner membrane, nucleolus, ribonucleoprotein complex, cytosol, nucleus

Figure 6

Click here to access/download;Figure;Figure 6_Bi_directional methylation calling samples 57_58.docx

Figure 6: Bi-directional methylation calling comparing samples 57 and 58 to validate the ONT methylation calling. Heatmap (A) compares sample 57 as the signal-level data against sample 58 as the biological replicate, (B) is the reverse.

(A)

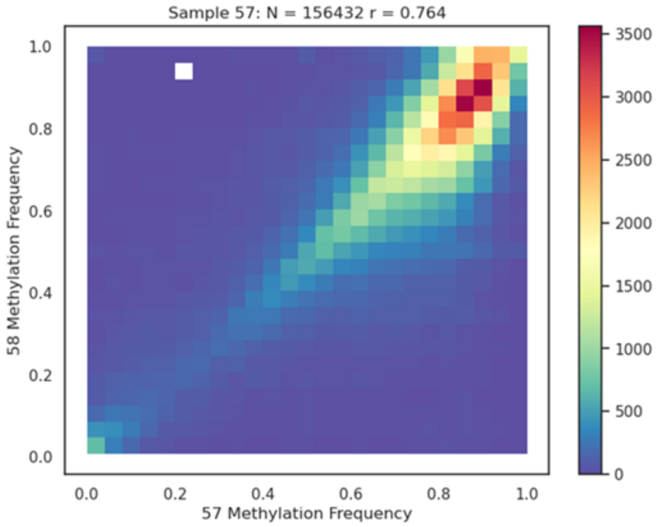

(B)

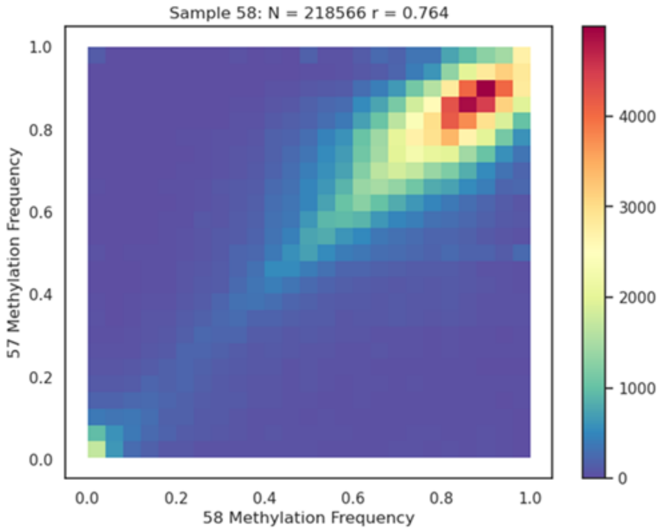

Click here to access/download;Figure;Figure 7_Methylation versus unmethylated gene frequencies.docx

Figure 7: Methylated versus unmethylated gene frequencies for the 5 assemblies relative to genome size (according to number of genes). Most genes in samples 57 and 58 were methylated and hence transcriptionally inactive. In samples 70, 108 and 115 the majority of the genes were not methylated (i.e., the unmethylated frequencies fell below 0.3) and hence being expressed.

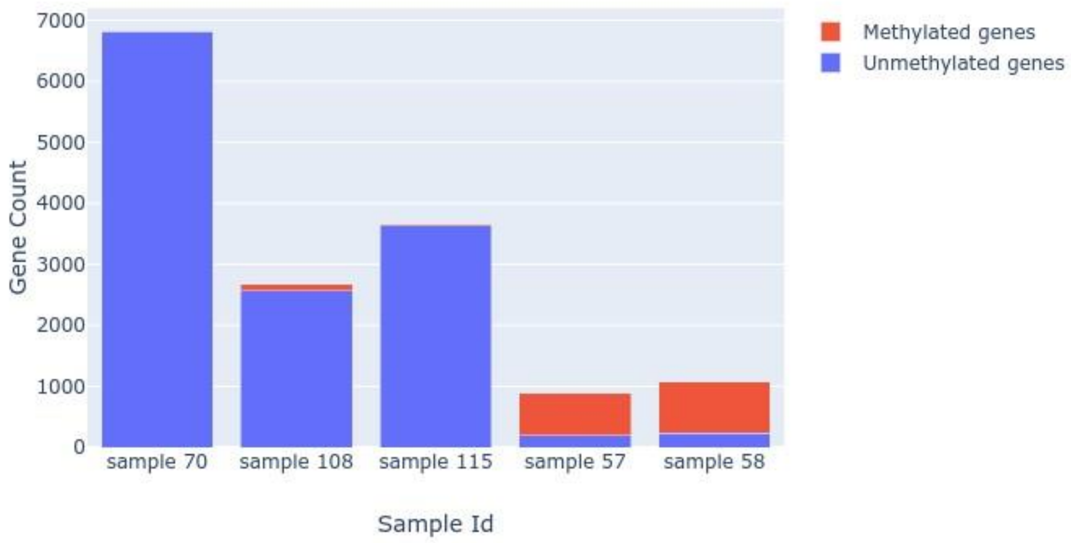

Figure 8

Click here to access/download;Figure;Figure 8 Kernel density of methylation.docx

Figure 8: Kernel density estimation of methylation of CpG sites in CDS regions. (A) The elbow method determines the optimal number of clusters in the data set. The inertia curve starts to flatten at a position indicating there are 2 clusters in this dataset. (B) Unweighted (blue) and methylation frequency weighted Gaussian KDE (orange) probability density function curves with distinct peaks denoting two clusters representing methylated and unmethylated portions of the genomes.

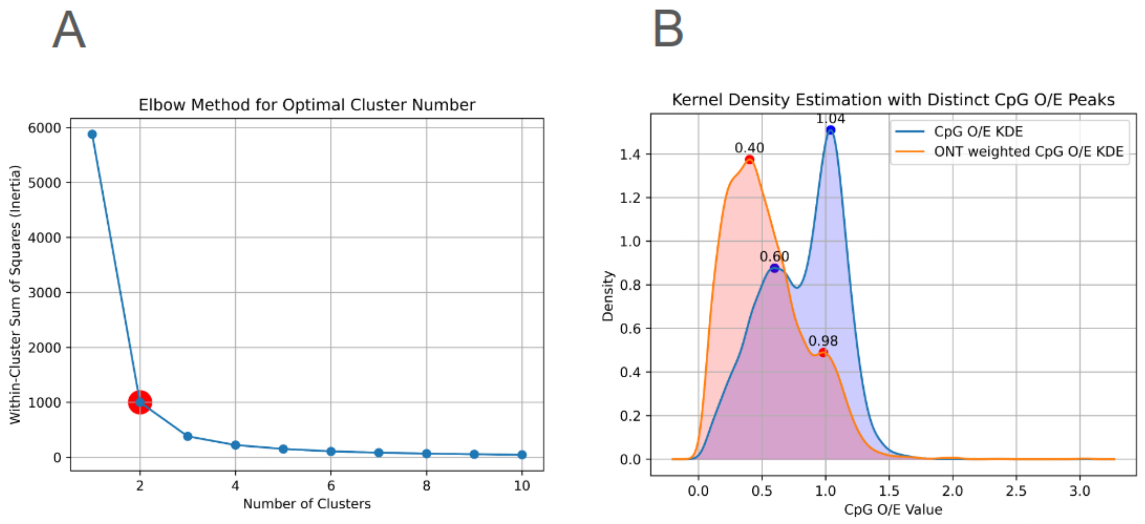

Click here to access/download;Figure;Figure 9 Savitzky-Golay plot of CpG ratio .docx

Figure 9: Correlation between CpG O/E values for CDSs as an indicator for CpG methylation. Savitzky-Golay filter smoothed plot displaying 1000 lowest CpG O/E CDSs cumulative CpG methylation frequencies (red) and 1000 highest calculated CpG O/E CDSs cumulative methylation frequencies (blue). Average values for each set methylation frequency is displayed with a dotted line with value reported in the plot legend.

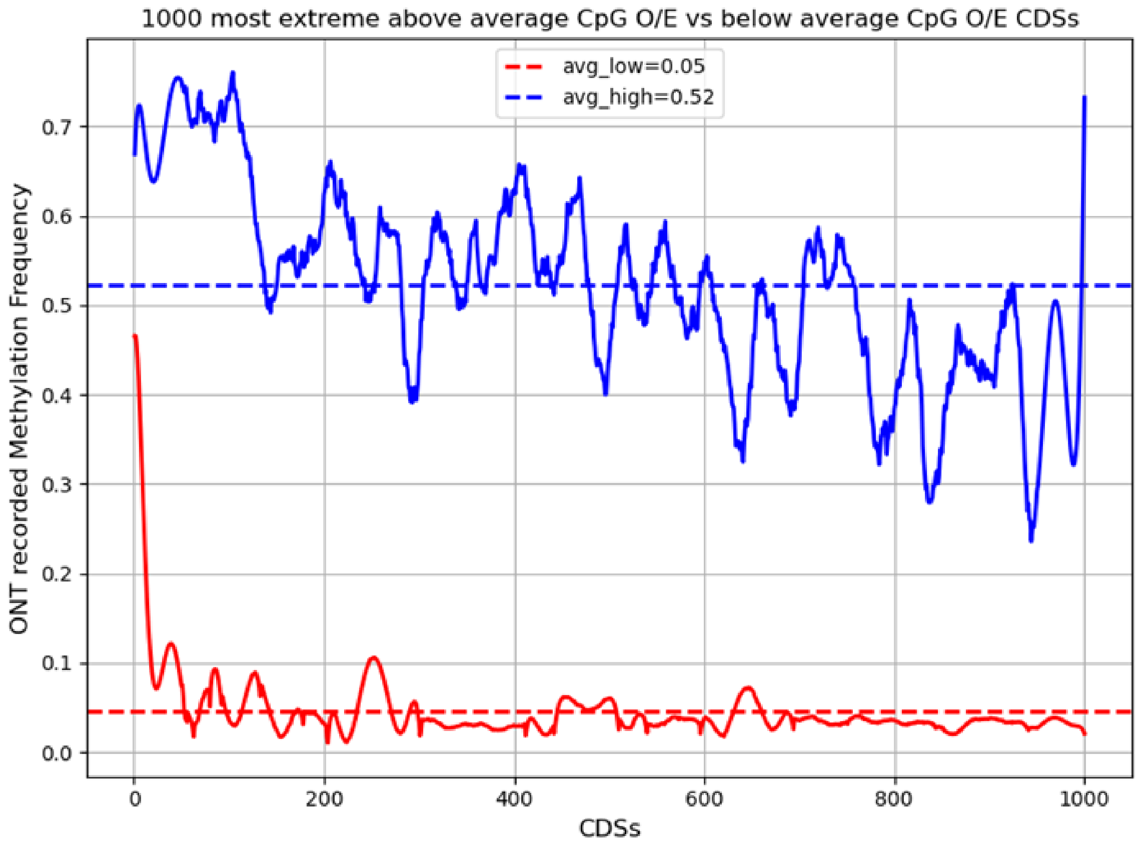

Click here to access/download;Figure;Figure 10 Annotation of methylated vs unmethylated CDSs.docx

Figure 10: Possible functions of methylated and unmethylated CDSs. (A) Linear relationship between gene/CDS size and number of available CpG sites as shown by linear regression analysis and measured using Pearson correlation coefficient r = 0.86 (B) KeyBERT keyword extraction set intersection between unmethylated and methylated genome portions of all complete Myxozoa genome assemblies. The relationship between these two portions is displayed as a Venn diagram, in which the unmethylated portion is the bigger part, with a small number of shared keywords related to protein handling being the only similarity to the methylated portion.

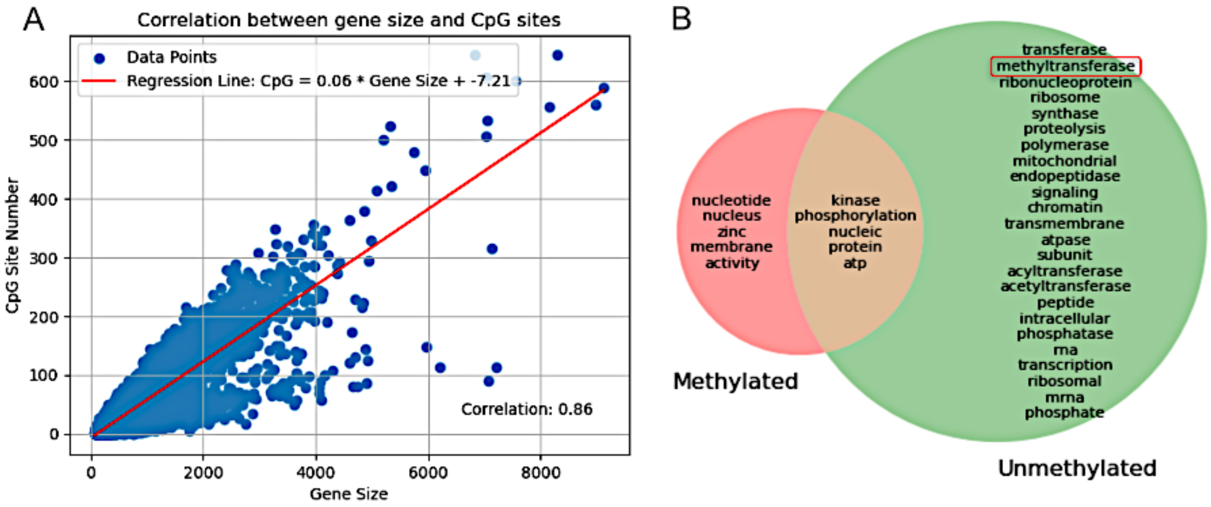

Click here to access/download
Supplementary Material
Supplementary File 1_Reference fish genomes.xlsx

Click here to access/download
Supplementary Material
Supplementary File 2_Available myxozoa genomes.txt

Supplementary Material 3A

Click here to access/download
Supplementary Material
sample_57_rRNA_seq.fasta

Supplementary Material 3B

Click here to access/download
Supplementary Material
sample_58_rRNA_seq.fasta

Supplementary Material 3C

Click here to access/download
Supplementary Material
sample70_rRNA_seq (3).fasta

Click here to access/download
Supplementary Material
sample108_rRNA_seq (1).fasta

Click here to access/download
Supplementary Material
sample115_rRNA_seq (1).fasta

Click here to access/download
Supplementary Material
Supplementary File 4_Photos of parasites and fish hosts.pptx

Click here to access/download
Supplementary Material
Supplementary File 5_Myxozoa_SSU_annotated.docx

Click here to access/download
Supplementary Material
Supplementary File 6A_Myxozoa phylogenetic tree.eps

Click here to access/download
Supplementary Material
Supplementary File 6B_Newick representation phylogeny.txt

Click here to access/download
Supplementary Material
Supplementary File 7_Assembly statistics.pdf

Click here to access/download
Supplementary Material
Supplementary File 8_Word cloud analysis.docx
