# Supplementary material for: Long-read metagenomic sequencing negates inferred loss of cytosine methylation in Myxosporea (Cnidaria: Myxozoa)
Source: Gigascience. 2025 Mar 13;14:giaf014. doi: 10.1093/gigascience/giaf014 (PMC11905887; doi:10.1093/gigascience/giaf014)
Supplement: giaf014_Response_to_Reviewer_Comments_Original_Submission [file giaf014_response_to_reviewer_comments_original_submission.pdf]

Author's Response To Reviewer Comments

Reviewer #1:

Comment 1. Does the selected sample sufficiently represent the genetic diversity of the target Myxozoa population? Could the authors elaborate on the criteria and rationale for sample selection?
We find this comment somewhat obscure. Our aim was to detect whether there is evidence for methylation in myxozoans, not to explore genetic diversity in populations. Our overall 'target population' was therefore the 5 genomes we sequenced plus the myxozoan genomes already available from other studies. In terms of the material we specifically collected, we have shown that genome sequences for samples 70, 108 and 115 are different from each other and from samples 57 and 58, which are biological replicates. Hence, the data we have generated for this study does represent genetic diversity in the population sampled from Amazonian fish hosts. We acknowledge that we did not provide information on how we 'selected' the samples, but we have now addressed this by adding the following text on page 8 of the Methods:
' Our aim was to sample opportunistically for myxozoans in Amazonian fish gall bladders, which have been commonly infected by these cnidarian parasites (44). After the dissection of each fish the gall bladder was removed and a sterile scalpel blade was used to open the gall bladder pouring the bile directly into a sterile 1.5 mL Eppendorf tube. With a sterile micro-pipette, a drop of the bile fluid was immediately placed on a microscope slide and examined using a light microscope (Carl Zeiss model Primo Star) at 20x or 40x to ascertain infection. When infection was confirmed, the bile fluid content of the Eppendorf tube was immediately preserved in DNA/RNA Shield reagent (Zymo Research Corp., Irvine, CA, USA).
And to page 19 of the Results where we discuss sampling fish hosts:
'We detected myxozoan infections in 102 out of 189 fish gall bladders (54%). The DNA of myxozoans collected from the bile of five fish specimens was isolated in sufficient concentration and purity required for ONT sequencing. Just over 9 terabytes of sequencing data were generated.'

Comment 2. Based on genomic synteny analysis, how do the researchers ascertain the homology relationships between samples? Is there a possibility of mixed infections or other complex scenarios that could influence this determination?
In addition to genomic synteny analysis, we did extract 18S rRNA genes and used these for both taxonomic placement of our samples, and to assess the overall genomic homology between samples. Contamination with host DNA is inevitable, and to assess the extent of this in our samples, we developed a stringent quality criterion in which we screened both our assembled genomes and the ONT long reads to "fish out" 18S rRNA genes that we could then analyze using the latest Silva (SILVA Release 138.2) SSU NR portion of the database. This analysis confirmed that our genome assemblies indeed represented single myxozoan species in various degrees of completeness, with the only other detectable SSUs coming from bacteria, fish hosts and human contamination. Since submission of our manuscript, the genomes assemblies have also passed the NCBI host contamination filtering process and are now deposited with their own individual accession numbers, which have been included in the data availability statement on page 41.

Comment 3. The manuscript mentions the generation of over 9TB of sequencing data using Oxford Nanopore PromethION technology and steps taken to mitigate host DNA contamination during data analysis. However, the original proportion of host to myxozoan DNA in the samples is not detailed. Clarification on this proportion is essential for understanding the complexity of the samples and the accuracy of subsequent analyses.
Host DNA contamination is inevitable in all myxozoan sequencing attempts reported to date due to the endoparasite lifestyle of these animals. It is accepted that Myxozoa have undergone quite drastic genome reduction whereas fish hosts, on other hand, have likely had whole-genome duplication events during their long evolutionary histories. This inevitably leads to imbalanced genomic datasets. For our data, parasites are the minority class, while the fish hosts represent the majority. Acknowledging this, we have used well established imbalance dataset training reasoning and applied this during genome assembly. In particular, we have used under-sampling of all host related reads when preparing our assembly datasets. Furthermore, after resolving the assembly contigs, we used BlobTools and, based on several comprehensive BLAST searches, judiciously removed all suspicious contigs.
We have included the following text to page 21 of the Results:

Overall, this produced satisfactory results despite the initial imbalance ranging from 10 to 1 in favor of host DNA in Ellipsomyxa sp. sample 115, up to 4 to 1 in Ceratomyxa sp. sample 58. The only sample that had relatively more parasite reads compared to the host was Ellipsomyxa sp. sample 70, with a 2 to 1 ratio in favor of parasite DNA. These calculations were based on ratios between excluded reads (i.e., ones that mapped onto reference fish genomes) and reads that mapped onto contamination pruned assemblies.
We have included the following text to page 32 of the Discussion:
Although the ratio of host to parasite reads is important to mitigate host contamination, in our experience when handling ONT sequencing datasets the actual quantity of DNA sequencing template has a much greater impact on the final completeness of the genome assemblies. compared to Illumina based NGS. This is because the read quality and read length impacts subsequent analyses, and we found these to be variable amongst the samples. For example, Ellipsomyxa sp. sample 70 had significantly longer reads of better quality on average, when compared to the rest of the samples.

Comment 4.The potential for genomic contamination is acknowledged in the study, but its impact on the results is not thoroughly discussed. How did the researchers assess the extent of contamination, and how might it affect the interpretation of the myxozoan genomes?
We thank the referee for this comment and address this by adding the following text to page 32 of the Discussion:
Host DNA contamination is almost inevitable in all Myxozoa sequencing attempts due to the endoparasite lifestyle of the animals. This will lead to false interpretation of Myxozoa genome sizes and introduce confusion in understanding of evolutionary relationships due to very diverse host ranges. To reduce the extent to which this contamination might affect interpretation of myxozoan genomes was reduced herein by using minimap2 to map all reads onto a large set of reference fish genomes, and then assembling the non-mapping reads. These assembled contigs were then screened by BLAST comparison against comprehensive databases such as NT and tsa_NR in order to remove even the slightest remaining homologies to fish hosts.

Comment 5. While Nanopolish was utilized for DNA methylation detection, considering the limitations mentioned for non-targeted WGBS in myxozoan genomic studies, could the authors consider targeted capture WGBS as a validation method? Given the low methylation levels observed in sample 70, have additional experiments been conducted to ensure these signals are not false positives from CpG site identification by Nanopore?
The referee is correct, WGBS is used as a validation method for ONT based methylation calling. There are even readily available scripts provided by ONT for this task:
https://nanopolish.readthedocs.io/en/latest/quickstart_call_methylation.html. However, we were unable to perform such a validation test in this study. This was because sample collection was in a very remote location, and we could not perform additional sampling nor could we preserve sufficient quantities for repeated studies and avoid DNA degradation. Therefore, we have used a biological duplicate in case of samples 57 and 58, which contain the same species of Myxozoa, from the same fish host, sampled independently and processed separately. Using these samples, we performed the same validation as would have done using WGBS as a control. In a cross-reference manner, akin to a bi-directional blast approach, we mapped reads from sample 57 onto sample 58 assembly and vice-versa, whilst performing methylation calling in both cases. The resulting methylation frequencies per site were compared and a correlation statistic calculated. The results obtained were comparable to most studies using WGBS (as a control), so we are confident in their accuracy. Sample 70 is our best genomic sample, based on multiple criteria (assembly completeness, read quality, coverage). Due to very high read coverage (>300x), methylation frequencies inferred from this sample data are as reliable as one can get. Each methylated/unmethylated CpG location was assessed based on at least 300 individual ONT read signals encoded in fast5 files, making the probability of false positives/negatives virtually non-existent. Regarding the low methylation level in this sample, this is comparable to methylation levels in samples 108 and 115, and is in line with previous work by Kyger et al. We believe the stark differences in levels of genome methylation might be explained by the stage of the life cycle when the DNA was extracted. We have mentioned in the discussion that further work needs to be done to prove this experimentally, but it certainly opens new and challenging avenues of research in Myxozoa.

Comment 6. The manuscript refers to the CpG Observed/Expected (O/E) ratio as an indicator of DNA methylation, but the specific biological implications and influencing factors of this ratio in different genomic

regions, such as CDS and non-CDS, are not fully explored. Could the authors further elucidate the biological significance of the CpG O/E ratio and its role in gene regulation?
CpG O/E values were used as the input for MethMod - a Gaussian Mixture Model (https://github.com/JanLeipzig/MethMod/) to either prove or disprove the existence of epigenetic capabilities of various invertebrates based solely upon genome data. In this regard, our samples displayed a methylation associated pattern of CDS CpG O/E values consistent with other invertebrates known to methylate their DNA.
We have added the following text to page 36 of the Discussion to explain the biological significance of CpG O/E patterns:
Determining the overall genomic CDS CpG O/E ratio is a very significant indicator of DNA methylation and in essence is quite a simple concept. Any CDSs that are often methylated will inevitably display markedly lower CpG O/E values than sequences that do not. This is due to an increased rate of mutation in methylated CpG that leads to GC content loss, because C-to-T transitions are the most common type of mutation at these sites. In terms of resultant distribution, this GC loss will lead to a characteristic two-peak bimodal distribution which is a characteristic that has been observed in all invertebrate species known to methylate DNA (Figure 8). Additionally, when the distribution of the ratio between CDS and non-CDS regions of the genomes were compared, most suspected methylation events occurred in CDS regions as expected, since this is a tell-tale mark of invertebrate methylation (unlike in vertebrates which skip non-CDS regions). The GC content has significant biological consequences reflected in gene inactivation and genome instability that could explain reported variation in Myxozoa genome sizes, and overall high fragmentation and difficulties in assembly and annotation of genomes.

Comment 7. The use of Kernel Density Estimation (KDE) analysis to reveal a bimodal methylation pattern is intriguing, yet the biological significance of this pattern is not clearly explained. Could the authors provide more detail on how the KDE analysis supports the existence of two distinct methylation states in CDS populations and the potential impact of this finding on understanding gene regulatory mechanisms in myxozoans?
KDE was used as a fully non-parametric method to prove the existence of two distinct gene sub-populations typical of methylation patterns observed in invertebrates, without having to assume a particular distribution is present. The KDE results clearly indicated the presence of a bimodal distribution composed of one cluster of CDSs with lower CpG O/E values, and second with higher CpG O/E values - which indicates presence of preferably methylated and preferably unmethylated CDSs within the same genome (Figure 8). In addition, KDE provides a more flexible and robust method for estimating density functions, especially when the underlying distribution is not strictly Gaussian, which is a possibility we needed to consider given that Myxozoa are known to display genome reduction. By confirming the bimodal pattern using KDE, we strengthened the evidence for the existence of two distinct methylation states by analyzing CDS populations, and validated results obtained using MethMod. However, we must say that this does not reveal anything on the underlying methylation mechanisms except confirming that they exist. One additional thing we did was to implement weighted KDE, where we used epigenetic data and ONT nanopore based methylation frequency results in its full reported range as weights, and genomic based CpG O/E values as data points. Adding weights did not change the distribution, which was still bimodal, nor did it significantly shift the low CpG O/E cluster peak. However, it did highlight the low CpG O/E cluster that is being methylated and shifted its peak to a lower value. Using epigenetic data as weight has also inverted the peak heights that represent probability density of observing a data point at that specific value. This allowed us to make more precise CpG O/E boundaries that can be used to pinpoint which CDSs are being actively methylated, and which are not and it also confirmed our hypothesis that large number of gene bodies are being methylated/demethylated in an unison manner, hence such an inversion of peak heights with a methylation frequency weighted KDE compared to unweighted one based solely on genome level CpG O/E data.
We have added the following text to page 37 of the Discussion:
The combination of epigenetic data with the CpG O/E value calculated from the genome assemblies in the form of a weighted KDE, more precisely defined CpG O/E boundaries and additionally more accurately pinpointed which CDSs were being actively methylated. We believe this is the first study to use epigenetic methylation frequency results in combination with genomic derived CpG O/E values to assess the frequency of methylation at specific genetic loci. This approach has potential as a preliminary screening method to identify genes of interest for targeted experimental approaches to resolve epigenetic processes, including regulatory mechanisms.

Comment 8. The manuscript proposes a "transcription switch" mechanism for gene expression regulation in myxozoan genomes. To substantiate this hypothesis, has consideration been given to quantitative PCR (qPCR) experiments to compare gene expression levels under varying methylation conditions? If feasible, could experiments be designed to measure transcription levels of CDSs with high and low CpG O/E values to determine if methylation status correlates with gene expression repression or activation? Such experiments would provide functional evidence for the "transcription switch" model and contribute to understanding the role of DNA methylation in gene regulation in myxozoans.
We thank the referee for their comment and fully agree that additional work needs to be done to substantiate our hypothesis. However, this is outside of the scope of the work described herein, which was to determine if DNA methylation exists in Myxozoa. Therefore, we have now omitted this “transcription switch” hypothesis from the text, including the title and have rewritten the abstract. This hypothesis has been fueled by stark difference between samples 57/58 and the rest of the samples methylation frequency findings we report herein. The weighted KDE method as explained above has further strengthened our initial suggestion that the most plausible explanation of this experimentally confirmed difference would be a most rudimentary mechanism in which a large number of gene bodies are being methylated/demethylated in unison, hence the “switch” parallel. Using experimental evidence we obtained (methylation frequencies) and “theoretical” data - CpG O/E values of CDSs, we were able to provide additional evidence for this, but as you noted further work is a proposition we shall certainly undertake in near future.

Reviewer #2:
Comment 1. 'transcription switch' mechanism: The main assertion of the paper states that “experimental and in silico analyses revealed variable methylation levels genomes, suggesting a 'transcription switch' mechanism regulating gene expression,” as noted in the abstract. However, there appears to be a significant gap between sequence analysis and the proposed mechanism. Could the authors provide additional validation for this finding through cross-platforms sequencing, such PacBio HiFi and WGBS? Although the authors mention associated with WGBS, incorporating data from multiple sequencing technologies could strengthen their conclusions. Furthermore, given that most assembled genome CpG sites are methylated, suggesting that many genes may be transcriptionally silenced, transcriptomic analyses could serve as compelling evidence for this finding. Additionally, the identification of DNA methyltransferase (DNMT), ten-eleven translocation methylcytosine dioxygenase (TET), and base excision repair (BER) enzymes warrants further investigation. A comparative analysis of these proteins with their homologys, particularly focusing on their functional domains, would be meaningful to support the authors’ conclusions.
We fully agree with the referee that our findings need to be further validated, including cross-platform sequencing and comparative analysis of proteins related to methylation mechanisms. However, as we have provided in response to Referee 1 Comment 8, these experiments are outside of the scope of the current manuscript, which aimed to determine if DNA methylation occurs in Myxozoa. Also, as far as we are aware, it is only ONT nanopore that has the capability of addressing both base-calling and methylation calling tasks in a single run, and this is the reason why it was used here. Cross-platform sequencing could be used to improve existing genome assemblies and indeed we are already planning to use it for continuation of the work described here. Regarding your comment on CpG sites, where you stated that “most assembled genome CpG sites are methylated”, in our results section we in fact reported the opposite of this. As we clearly stated and provided sufficient experimental evidence (ONT recorded methylation calls, additionally validated through one biological replicate), only sample 57 and 58 (the biological replicate) provided this outlook where most CpG sites within genome bodies has been extensively methylated, while the remainder of samples - 108, 115 and sample 70 were total opposites, with only a minor fraction of CDSs showing signs of CpG methylation. This has led us to name this putative mechanism 'transcription switch'. Now when looking at this expression which we came up with, in the light of your very insightful comments, we believe “epigenetic switch” might have been a more proper phrase. Nevertheless, concurrent with your comments and Referee 1 Comment 8, we have removed the concept of a transcriptional switch from the manuscript, including the title and have re-written the abstract.
Comment 2. Pangenome Analysis: The utilization of pangenome analysis is generally intended to assess genetic diversity within populations of a single species or closely related species. However, as this study encompasses several different myxozoan species, the relevance of a pangenome approach is questionable. The authors should consider reframing this section as “genome comparison” if no specific pangenome graphs or structural variants are presented.

We completely with the referee’s comment and have rephrased throughout the manuscript and figures.
Comment 3. Word Cloud Analysis for GO Terms: While I understand the rationale behind using a word cloud analysis to highlight overrepresented protein functions within the core gene repertoire, this may not be necessary because no strong relationship with your propose. GO term comparisons can often be too vague to provide specific insights into study objectives, particularly for this research.
After carefully assessing your comment, we have come to the conclusion that our explanation of MethMod and KDE related analyses might have been too vague, and we sincerely apologize for this. We believe this might have obscured the actual intent behind our GO work presented. The major reason why we performed the word cloud analysis was not to highlight overrepresented protein functions, since we performed no transcriptomics or proteomics in this research. The reasoning behind the GO based word cloud analysis was to display the most prominent functions accumulated in what we believe are preferably methylated gene bodies and preferable non-methylated ones. These groups (or clusters) have been determined based on extensive modeling effort, first using MethMod parametric Gaussian mixture modeling and followed by non-parametric KDE, in order to validate and confirm our findings and grouping of the gene bodies in preferably methylated/unmethylated clusters. As described above, we went one step further, by combining epigenetic and genetic markers - the methylation frequencies of CpG sites within gene bodies, with the gene bodies probabilistic based CpG O/E values, that we used to assess long-term exposure of these gene bodies to methylation/demethylation events. You are right to state that this might be inconclusive, since obviously no proteomics or transcriptomics data has been presented, however as you also noted, there is a strong rationale behind these word clouds, they were based on strong statistics and our intention was to suggest future transcriptomics and proteomics based work.
Comment 4. Claims Regarding Database Matches: In the abstract, the authors state, “These genomes did not match any existing sequences in public databases, suggesting a need for updated sequence data for these elusive parasites.” This claim appears exaggerated potentially misleading, as I could not find where this assertion is supported in the manuscript. I recommend rephrasing this statement and including additional discussion to substantiate it.
We agree with the referee’s comment. This sentence has been removed from the abstract.
Comment 5. Genome Completeness: The authors performed assembly quality assessments, including N50, N90, BUSCO analyses. However, the BUSCO results alone cannot substantiate the claim of the genome being “complete” (sample 70), as it only covered half of the complete BUSCOs (123 out of 255), and the estimated genome size is considerably smaller that of other previously sequenced myxozoans, which range from 61.44 to 234.48 Mbp. I suggest the authors directly map the ONT reads to their assembly to measure genome quality and provide a quality value (QV). Furthermore, I caution in the use of the term “complete” when discussing genome assembly.
We have changed “complete” into “best quality” throughout the text in order to make this statement more acceptable. We do agree that no available metrics is sufficiently good enough to be able to claim genome completeness. It was not our intent to claim we have a “complete” genome, it was our intent was to state that we have “the most complete” genome when compared to previously assembled myxozoan genomes. Based on these metrics we have presented; we believe this is accurate.
Regarding the BUSCO completeness, this is especially difficult to address since these parasites are known to undergo genome reduction, so we did not expect to obtain a full set of BUSCOs, or to assess how many might be retained. Regarding your suggestions on mapping, we performed multiple rounds of ONT read mappings onto each of our genomes. This was extensive work, and it is included in our genome assembly pipeline as well as species confirmation.
To clarify, here is what was exactly done in this regard: In the read filtering stage, as part of the host contamination removal, we have mapped all fastq reads onto reference fish genomes and used only the unmapped ones for the subsequent genome assemblies. The reasoning behind this being the fact that unmapped reads are obviously enriched with the parasite DNA. After performing the Flye based assembly, we have evaluated obtained genome sizes using kmer counts, as described in the manuscript, but we have also remapped all reads back onto the alignment in order to assess coverage (whether all assembled contigs are evenly covered) and also as part of the BlobTools removal of suspicious contigs that might harbor some contamination that we failed to remove in previous step. BlobTools provides interactive quality assessment based exactly on the mapping you are mentioning, but also based on additional blast search reports for which we used two comprehensive databases (euk_nt and tsa_nr).
Comment 6. Genome contamination: According to Supplementary File 5, sample 70 appears to exhibit two peaks in GC content. Does this indicate possible contamination? The authors should provide more regarding this observation and assess how it may affect the interpretation of the myxozoan genomes.

This was also raised in several comments by Referee 1 for which we have also offered an explanation and additional text. Please see our responses to Referee 1 comments 3, 4, 6 and 7.
Briefly, two GC content peaks were expected based on our findings using MethMod and weighted KDE to analyze sample 70. One peak (with greater GC content) represented CDSs that were likely methylated, and a second peak (lower GC content) which represented CDSs which were not. This is characteristic for invertebrate genome methylation rather than contamination. To further rule out contamination, we used exhaustive filtering plus BlobTools post assembly. Additionally, we performed 18S rRNA analysis by analysis of the entire SSU molecule for variable and hypervariable regions (which we now include in the revised manuscript as Supplementary File 5) and by performing a phylogenetic analysis that confirmed this sample represented a single distinct species of Myxozoa. This phylogeny is uploaded to the GigaDB for review by the referees and we have included a new Supplementary File 6 into the Results section together with supporting text in the Methods section, thus:
Phylogenetic analysis encompassing 678 Myxozoa 18S rRNA sequences, where 673 have been obtained from Silva database (ref) with the addition of 5 more extracted from our genomes. The analysis was based on multiple sequence alignment where all insert columns have been removed, including any consensus column for which fewer than 95% of the sequences that are nongaps and have a posterior probability of less than 0.95. This data preparatory process, which assumes all aligned nucleotides are homologous, ensures that the resulting phylogeny is based on robust data. The alignment and variable region masking have been performed using SSU-ALIGN (PMID: 11869452, and the phylogenomic inference including tree reconstruction has been accomplished using IQ-TREE (PMID: 32011700) installed on a Linux server. The built-in "ModelFinder" feature (PMID: 28481363) has been used to determine the best-fit substitution model for 18S DNA multiple sequence alignment input. For the visualization of the resulting unrooted tree, iTol v6 has been used (PMID: 38613393) has been utilized.
Additional Suggestions:
Comment i: Ensure consistent sample descriptions and avoid using sample IDs when describing species.
Consistent species descriptions have now been used throughout the manuscript, tables and figures.
Comment ii: Figure quality should be improved; the workflow should be clearly presented, and legends should be complete.
This has been corrected.
Comment iii: The BUSCO figure appears to be incorrect, as sample 70 is indicated to have the most complete BUSCOs but with a short blue bar.
The referee is mistaken, there is no error in the BUSCO figure. BUSCO reports complete and single copy, complete and duplicated, fragmented and missing BUSCOs. These four categories are represented by different colours which are included in the legend. Confusion may relate to the first two categories that overlap on "complete" BUSCOs. Since these overlap, we ask the referee to look at where the green bar stops and not the blue bar. It should then be clear that sample 70 is indeed the most complete genome and, therefore, that the figure is correct. Other genomes that also exhibit duplicated complete BUSCOs display similar "anomaly" (Kudoa iwatai and Sphaeromyxa zaharoni), which is not an error but simply the fact that one needs to account for overlapping sets of BUSCOs. Extending the blue bar representing complete BUSCOs and then repeating this for the portion of complete BUSCOs that are also being duplicated would not be correct.
Comment iv: CDS stands for coding sequences, not coding regions.
This has been corrected throughout the text.
