# Supplementary material for: Long-read metagenomic sequencing negates inferred loss of cytosine methylation in Myxosporea (Cnidaria: Myxozoa)
Source: Gigascience. 2025 Mar 13;14:giaf014. doi: 10.1093/gigascience/giaf014 (PMC11905887; doi:10.1093/gigascience/giaf014)
Supplement: giaf014_Response_to_Reviewer_Comments_Revision_2 [file giaf014_response_to_reviewer_comments_revision_2.pdf]

Author's Response To Reviewer Comments

Please see the Personal Letter uploaded
