# Supplementary material for: Long-read metagenomic sequencing negates inferred loss of cytosine methylation in Myxosporea (Cnidaria: Myxozoa)
Source: Gigascience. 2025 Mar 13;14:giaf014. doi: 10.1093/gigascience/giaf014 (PMC11905887; doi:10.1093/gigascience/giaf014)
Supplement: giaf014_Reviewer_1_Report_Original_Submission — Jiayong Zhong -- 7/16/2024 [file giaf014_reviewer_1_report_original_submission.pdf]

Reviewer Report

Title: Long read metagenomic sequencing negates inferred loss of cytosine methylation in Myxosporea (Cnidaria: Myxozoa)

Version: Original Submission **Date:** 7/16/2024

Reviewer name: Jiayong Zhong

Reviewer Comments to Author:

This study utilized Oxford Nanopore sequencing to successfully assemble five myxozoan genomes, surpassing previous studies in both quality and completeness. Genome assembly revealed DNA methylation patterns within myxozoan genomes, particularly in GC-rich regions within gene bodies. The findings not only refute the notion of myxozoans lacking DNA methylation capability but also propose a potential "transcription switch" mechanism, offering a new perspective on gene regulation in these parasites. Furthermore, the high-quality genome assemblies lay a solid foundation for further biological research on myxozoans.

The article mainly used Oxford-Nanopore to study the myxozoans genome and its methylation, suitable for research purposes. The methodology is described in relative detail, including the collection of samples, DNA extraction, sequencing, and the subsequent process of bioinformatics analysis. The details of the statistical analysis and the data in the article are sufficient, and the conclusions are still supported by the data, but the proposed mechanism of "transcriptional switch" should be supported by additional experiments.

The research presented in this manuscript is indeed significant and contributes meaningfully to the field. However, there are several queries that necessitate further response and additional detailed descriptions:

1.Does the selected sample sufficiently represent the genetic diversity of the target Myxozoa population? Could the authors elaborate on the criteria and rationale for sample selection?

2.Based on genomic synteny analysis, how do the researchers ascertain the homology relationships between samples? Is there a possibility of mixed infections or other complex scenarios that could influence this determination?

3.The manuscript mentions the generation of over 9TB of sequencing data using Oxford Nanopore PromethION technology and steps taken to mitigate host DNA contamination during data analysis. However, the original proportion of host to myxozoan DNA in the samples is not detailed. Clarification on this proportion is essential for understanding the complexity of the samples and the accuracy of subsequent analyses.

4.The potential for genomic contamination is acknowledged in the study, but its impact on the results is not thoroughly discussed. How did the researchers assess the extent of contamination, and how might it affect the interpretation of the myxozoan genomes?

5. While Nanopolish was utilized for DNA methylation detection, considering the limitations mentioned for non-targeted WGBS in myxozoan genomic studies, could the authors consider targeted capture WGBS as a validation method? Given the low methylation levels observed in sample 70, have additional

experiments been conducted to ensure these signals are not false positives from CpG site identification by Nanopore?
6.The manuscript refers to the CpG Observed/Expected (O/E) ratio as an indicator of DNA methylation, but the specific biological implications and influencing factors of this ratio in different genomic regions, such as CDS and non-CDS, are not fully explored. Could the authors further elucidate the biological significance of the CpG O/E ratio and its role in gene regulation?
7. The use of Kernel Density Estimation (KDE) analysis to reveal a bimodal methylation pattern is intriguing, yet the biological significance of this pattern is not clearly explained. Could the authors provide more detail on how the KDE analysis supports the existence of two distinct methylation states in CDS populations and the potential impact of this finding on understanding gene regulatory mechanisms in myxozoans?
8. The manuscript proposes a "transcription switch" mechanism for gene expression regulation in myxozoan genomes. To substantiate this hypothesis, has consideration been given to quantitative PCR (qPCR) experiments to compare gene expression levels under varying methylation conditions? If feasible, could experiments be designed to measure transcription levels of CDSs with high and low CpG O/E values to determine if methylation status correlates with gene expression repression or activation? Such experiments would provide functional evidence for the "transcription switch" model and contribute to understanding the role of DNA methylation in gene regulation in myxozoans.

Methods

Are the methods appropriate to the aims of the study, are they well described, and are necessary controls included? Choose an item.

Conclusions

Are the conclusions adequately supported by the data shown? Choose an item.

Reporting Standards

Does the manuscript adhere to the journal's guidelines on minimum standards of reporting? Choose an item.

Choose an item.

Statistics

Are you able to assess all statistics in the manuscript, including the appropriateness of statistical tests used? Choose an item.

Quality of Written English

Please indicate the quality of language in the manuscript: Choose an item.

Declaration of Competing Interests

Please complete a declaration of competing interests, considering the following questions:

- Have you in the past five years received reimbursements, fees, funding, or salary from an organisation that may in any way gain or lose financially from the publication of this manuscript, either now or in the future?
- Do you hold any stocks or shares in an organisation that may in any way gain or lose financially from the publication of this manuscript, either now or in the future?
- Do you hold or are you currently applying for any patents relating to the content of the manuscript?
- Have you received reimbursements, fees, funding, or salary from an organization that holds or has applied for patents relating to the content of the manuscript?
- Do you have any other financial competing interests?
- Do you have any non-financial competing interests in relation to this paper?

If you can answer no to all of the above, write 'I declare that I have no competing interests' below. If your reply is yes to any, please give details below.

I declare that I have no competing interests.

I agree to the open peer review policy of the journal. I understand that my name will be included on my report to the authors and, if the manuscript is accepted for publication, my named report including any attachments I upload will be posted on the website along with the authors' responses. I agree for my report to be made available under an Open Access Creative Commons CC-BY license (http://creativecommons.org/licenses/by/4.0/). I understand that any comments which I do not wish to be included in my named report can be included as confidential comments to the editors, which will not be published.

Choose an item.

To further support our reviewers, we have joined with Publons, where you can gain additional credit to further highlight your hard work (see: https://publons.com/journal/530/gigascience). On publication of this paper, your review will be automatically added to Publons, you can then choose whether or not to claim your Publons credit. I understand this statement.

Yes Choose an item.
