# Supplementary material for: Long-read metagenomic sequencing negates inferred loss of cytosine methylation in Myxosporea (Cnidaria: Myxozoa)
Source: Gigascience. 2025 Mar 13;14:giaf014. doi: 10.1093/gigascience/giaf014 (PMC11905887; doi:10.1093/gigascience/giaf014)
Supplement: giaf014_Reviewer_1_Report_Revision_1 — Jiayong Zhong -- 10/17/2024 [file giaf014_reviewer_1_report_revision_1.pdf]

Reviewer Report

Title: Long read metagenomic sequencing negates inferred loss of cytosine methylation in Myxosporea (Cnidaria: Myxozoa)

Version: Revision 1 **Date:** 10/17/2024

Reviewer name: Jiayong Zhong

Reviewer Comments to Author:

Reviewer Response:
Thank you to the authors for addressing the majority of the comments and for providing detailed explanations and clarifications throughout the manuscript revisions. I find the authors' responses to most issues, such as sample selection, genomic homology, contamination, and data analysis methods, to be well-reasoned and satisfactory.
However, in **Comment 5**, while I understand the challenges the authors faced in performing whole-genome bisulfite sequencing (WGBS) validation due to the remote sample collection and the inability to preserve sufficient quantities of DNA, the key concern regarding the reliability of low methylation levels observed in sample 70 remains unaddressed in full. Although the use of biological duplicates and the cross-validation approach strengthens confidence in the accuracy of the methylation calling, the question of ensuring that the low methylation signals are not false positives still warrants further investigation.
Given the current limitations on obtaining new samples or performing additional WGBS validation, I propose an alternative approach: leveraging the abundant host DNA in the samples to indirectly assess the reliability of the Nanopore methylation analysis. By analyzing the methylation profile of the host genome (which constitutes a significant proportion of the data), the authors could use the well-characterized methylation patterns of the host species to verify the consistency and reliability of the methylation detection pipeline. If the host genes exhibit expected methylation patterns, this would provide strong indirect evidence supporting the accuracy of the findings in the myxozoan genomes.
If the authors are able to address this point by analyzing the host DNA methylation profile or offering other data to support the robustness of the methylation detection, I believe the manuscript would be ready for publication. Thank you again for the thorough responses, and I look forward to seeing the updated results.

Methods

Are the methods appropriate to the aims of the study, are they well described, and are necessary controls included? Choose an item.

Conclusions

Are the conclusions adequately supported by the data shown? Choose an item.

Reporting Standards

Does the manuscript adhere to the journal's guidelines on [minimum standards of reporting?] Choose an item.

Choose an item.

Statistics

Are you able to assess all statistics in the manuscript, including the appropriateness of statistical tests used? Choose an item.

Quality of Written English

Please indicate the quality of language in the manuscript: Choose an item.

Declaration of Competing Interests

Please complete a declaration of competing interests, considering the following questions:

- Have you in the past five years received reimbursements, fees, funding, or salary from an organisation that may in any way gain or lose financially from the publication of this manuscript, either now or in the future?
- Do you hold any stocks or shares in an organisation that may in any way gain or lose financially from the publication of this manuscript, either now or in the future?
- Do you hold or are you currently applying for any patents relating to the content of the manuscript?
- Have you received reimbursements, fees, funding, or salary from an organization that holds or has applied for patents relating to the content of the manuscript?
- Do you have any other financial competing interests?
- Do you have any non-financial competing interests in relation to this paper?

If you can answer no to all of the above, write 'I declare that I have no competing interests' below. If your reply is yes to any, please give details below.

I declare that I have no competing interests.

I agree to the open peer review policy of the journal. I understand that my name will be included on my report to the authors and, if the manuscript is accepted for publication, my named report including any attachments I upload will be posted on the website along with the authors' responses. I agree for my report to be made available under an Open Access Creative Commons CC-BY license (http://creativecommons.org/licenses/by/4.0/). I understand that any comments which I do not wish to be included in my named report can be included as confidential comments to the editors, which will not be published.

Choose an item.

To further support our reviewers, we have joined with Publons, where you can gain additional credit to further highlight your hard work (see: https://publons.com/journal/530/gigascience). On publication of this paper, your review will be automatically added to Publons, you can then choose whether or not to claim your Publons credit. I understand this statement.

Yes Choose an item.
